# Factors associated with the effectiveness of interventions to prevent obesity in children: a synthesis of evidence from 204 randomised trials

**DOI:** 10.1136/bmjph-2024-001707

**Published:** 2025-05-12

**Authors:** Annabel L Davies, Francesca Spiga, Deborah M Caldwell, Jelena Savović, Jennifer C Palmer, Eve Tomlinson, Theresa H M Moore, Carolyn D Summerbell, Julian P T Higgins

**Affiliations:** 1 Population Health Sciences, University of Bristol, Bristol, UK; 2 NIHR Applied Research Collaboration West, University Hospitals Bristol and Weston NHS Foundation Trust, Bristol, UK; 3 Department of Sport and Exercise Sciences, Durham University, Durham, UK; 4 Fuse - Centre for Translational Research in Public Health, Newcastle upon Tyne, UK

**Keywords:** Public Health, Body Mass Index, Systematic Review

## Abstract

**Introduction:**

We aim to identify effective characteristics of behavioural change (physical activity and diet) interventions that prevent obesity in children aged 5 to 18 years.

**Methods:**

We re-analysed data from two Cochrane systematic reviews published in 2024 using a Bayesian multi-level meta-regression analysis with intervention and trial characteristics coded according to an analytic framework co-developed with stakeholders.

**Results:**

We included 204 trials (250 intervention arms) reporting data on body mass index (BMI), either unstandardised or measured as a z-score (zBMI) or percentile. Interventions were effective on average (MD in zBMI −0.037, 95% credible interval −0.053 to −0.022). The greatest effects were associated with medium-term follow-up (nine to <15 months) and older children (12 to 18 years). We found evidence of small beneficial effects for interventions targeting physical activity alone compared with diet alone (difference in MDs −0.227,–0.362 to −0.090) and small unfavourable effects for interventions that involved a change to the structural environment (typically the school food environment) (difference in MDs 0.05, 0.017 to 0.085). Accounting for interactions between covariates, the most effective combination of intervention characteristics was to intervene in the school setting, with an individualised element to delivery, targeting physical activity, using multiple strategies of short duration and high intensity and involving modification of behaviour through participation in activities.

**Conclusions:**

The most effective characteristic to include in a behavioural change intervention to prevent obesity in children aged 5–18 years was targeting of physical activity. This should not be interpreted as evidence that attempts to modify diet are not beneficial. Being physically active and consuming a healthy diet during childhood offer many important benefits beyond contributing to healthy weight and growth. Our findings suggest that interventions to prevent obesity in children should consider increasing their focus on the promotion of physical activity and consider other effective characteristics we identify here.

**WHAT IS ALREADY KNOWN ON THIS TOPIC**

- Rising population levels of childhood overweight and obesity present a global challenge.
- Many behavioural change interventions (physical activity and/or diet) to try and prevent obesity in children and young people have been developed and evaluated, but the most effective characteristics of these interventions are not well understood.

**WHAT THIS STUDY ADDS**

- This re-analysis of the results of 204 randomised trials of diverse interventions found that the most effective characteristic to include may be targeting physical activity.
- Other useful characteristics of interventions appear to be individualised delivery, using multiple strategies, being intense and of short duration, and involving participation in activities.

**HOW THIS STUDY MIGHT AFFECT RESEARCH, PRACTICE OR POLICY**

- Our findings suggest that health policies targeting childhood obesity could benefit from focusing as comprehensively on physical activity as they already do on diet; health policies in schools could benefit from the inclusion of active participation in physical activity and, at least for young children (of primary school years) living in high-income countries, this being integrated within the school day, involving an electronic component and being perceived by children as ‘fun’.

## Introduction

 Rising population levels of childhood overweight and obesity present a global challenge,^1^ with profound implications for public health and health services.^2^ Children and adolescents living with obesity are more likely to experience reduced health-related quality of life and, for adolescents, a number of comorbidities.^3^ It is, therefore, important to prevent childhood obesity, both to ensure good long-term physical and mental health and to help children realise their full potential.^4^ Prevention requires effective interventions to change behaviour in relation to dietary habits and physical activity. Such behavioural change interventions typically contain multiple techniques or components,^5^ which may act individually or in combination to determine the effectiveness of the intervention.

We recently conducted two Cochrane systematic reviews of 244 randomised controlled trials of behavioural change interventions aimed at preventing obesity in children (aged 5 to 11 years and 12 to 18 years, respectively).^6,7^ Interventions were grouped according to whether they targeted physical activity, diet or both. While we found some modest beneficial effects, on average, of physical activity interventions, either alone or in combination with diet, there remained substantial between-trial variability in results across trials within each of the broad comparisons. This heterogeneity is most likely caused by variation in the characteristics of interventions included in each category. The characteristics varied in many different dimensions, including whether they targeted diet or physical activity or both, the degree of home (family) engagement, the degree of active participation by the children, the number of different strategies employed concurrently, the mode of delivery, the intensity and the duration.

In this paper, we report results of a re-analysis of these trials using all types of BMI outcomes reported, age groups (5–18 years) and follow-up times in a single comprehensive analysis. Our aim is to identify characteristics of these behavioural change interventions that are most strongly associated with their effectiveness in preventing obesity in children. We employ an analytic framework co-produced with stakeholders,^8^ statistical methods for mapping different outcomes onto a single measurement scale^9^ and a bespoke complex meta-regression model.^10^

## Methods

### Data and previous analyses

We used data from our two recently published Cochrane systematic reviews and meta-analyses, reported in detail elsewhere.^6,7^ In brief, the reviews included randomised trials (either individually randomised or cluster randomised) of behavioural change interventions that targeted dietary and/or physical activity in any setting that aimed to prevent obesity in children aged 5 to 18 years. Trials were excluded if they were restricted to children living with overweight and/or obesity as we were interested in prevention rather than treatment. Our outcomes of interest were unstandardised body mass index (BMI) and age- and sex-standardised BMI measured as z-scores (zBMI) or percentiles. For the Cochrane reviews, we calculated mean differences in change from baseline between the intervention groups in each trial. We calculated associated standard errors and, where appropriate, adjusted these standard errors for clustering.^6,7^ We use these mean differences and standard errors as the observations in our model. The dataset includes multi-arm trials as well as trials with multiple time points.

### Intervention-level coding

We developed an analytic framework to inform the synthesis. First, we sought to include intervention-level characteristics considered most likely to be associated with effectiveness. To compile these, we reviewed the international literature for relevant theories and frameworks. We engaged extensively with children and young people, teachers and public health professionals. We presented these groups with potential items and asked them to suggest characteristics they deemed most likely to be effective. Development of the framework is described in detail elsewhere.^8^

The finalised analytic framework comprises 12 main intervention characteristics (A to L in box 1). We coded each intervention in each trial according to these characteristics as described previously.^8^ Control arms, which we define as the absence of any intervention, were not coded. To reduce the number of variables, we applied dichotomisations to categorical variables aiming for divisions that resulted in the most even split of the data. In addition, we dichotomised intervention duration into ‘long’ and ‘short’ at the median duration across the trials. These dichotomous variables, which we call intervention-level indicators are also provided in box 1, with results of the coding presented in Section A of the online supplemental material.

Box 1 **12 intervention characteristics (and 25 resulting intervention-level indicators) included in our analytic framework.**

- The setting of the intervention (four sub-questions): whether the intervention…
  - was delivered in a school.
  - was delivered in the home.
  - was delivered in the community.
  - included a home activity.
- The mode of delivery (three sub-questions): whether the intervention was delivered to the child…
  - as part of a group of children.
  - individually.
  - electronically.
- The change of behaviour targeted by the intervention (two sub-questions):
  - dietary behaviours.
  - physical activity behaviours.
- The multi-factor nature of the intervention and its delivery (three sub-questions): whether the intervention was applied…
  - using multiple (three or more) different strategies.
  - in a single phase.
  - continuously.
- Intensity and duration (three sub-questions): whether the intervention…
  - was long (vs short) in total duration.
  - was long (vs short) in duration at its peak intensity.
  - involved a high (vs low) level of engagement at its peak intensity.
- Whether the intervention was integrated into usual activities.
- Whether there was flexibility in how the intervention can be implemented.
- Whether there was a level of choice available to children experiencing the intervention.
- Whether the intervention was considered to be enjoyable for the recipients (the ‘fun factor’).
- Whether the person/people delivering the intervention were likely to resonate with (inspire) the children.
- The mechanism(s) of action employed (four sub-questions): whether the intervention had an explicit component…
  - requiring the child to participate.
  - providing education/information to the child.
  - aiming to change the social environment of the child.
  - aiming to change the physical environment of the child.
- Whether there were commercial interests involved.

### Trial-level coding

In addition to intervention-level characteristics, it is likely that the effectiveness of interventions depends on characteristics of the participants. To investigate the possible impact of some broad societal inequities between populations, we defined trial-level indicators capturing the income status of the country (high vs non-high) and whether the trial specifically targeted participants (or communities) with low socio-economic status (SES). Details of how these trial-level indicators were categorised can be found in our Cochrane reviews.^6,7^

Rather than conducting separate analyses for the two age groups (as set out in our initial protocol^11^), we analysed the combined data set and included age group (5–11 vs 12–18) as another trial-level indicator. This allowed us to examine the differential effectiveness by age group, as well as to investigate how other factors interact with age, and provided additional power when examining factors across age groups.

### Time-point-level coding

We categorise follow-up times into short- (12 weeks to <9 months), medium- (9 months to <15 months) and long-term (15 months or more). We assume that the effect of follow-up time is common across trials and interventions. For studies that reported outcomes at more than one follow-up time within a particular category (short-, medium- or long-term follow-up), we selected the observation closest to the mid-point of the short- and medium-term intervals, and closest to 24 months for long-term observations.

In our Cochrane reviews,^6,7^ we conducted risk-of-bias (RoB) assessments for each trial result using the RoB 2 tool. Here, we coded these assessments, defining a dichotomous time-point-level indicator for whether each was judged to be at high risk of bias. Separate assessments were available for each time point, so the RoB indicator depends on both the trial and the follow-up time.

### Data analysis

#### Mapping of outcome to common measurement scale

We chose zBMI as our primary outcome because, unlike BMI, it accounts for the age and sex of the child and, unlike percentile, it was reported in a large number of trials. For trials that only reported results in terms of unstandardised BMI or BMI percentiles, we mapped these values onto the zBMI scale using methods we developed previously.^9^ To map from BMI to zBMI, we used a sampling method, implemented with 10 000 samples. The relationship between BMI and zBMI depends on an individual’s age and sex. Assuming a lognormal distribution for BMI, a normal distribution for age and a binomial distribution for sex, we set parameters of these distributions according to information reported from the trial. We then sampled 10 000 individuals from these distributions, calculated their zBMI and used these values to determine mean zBMI. To obtain zBMI from BMI percentile, we employed an analytic method that, assuming a normal distribution for zBMI, uses standard integral results to evaluate the expectation and variance of BMI percentile. For details of these mapping methods, we refer readers to our paper.^9^ In addition, nine trials reported results as the proportion of overweight or obese individuals. As described in our Cochrane reviews, we used normality assumptions to estimate mean zBMI from these values.^6,7^

#### Statistical model

To analyse our data, we used a bespoke multi-level meta-regression model described in detail previously.^10^ The model includes indicator variables (as covariates) defined on three levels: trial, intervention arm and time point. It assumes additive effects of all indicators while allowing for interactions between or within any level. An intercept term is included to capture the effect, relative to control, of an intervention whose indicators are all equal to zero. The mathematical details of our model are provided in Section B of the online supplemental material.

Each observation in the data is a mean difference between an intervention and a reference arm. The model takes a different form depending on whether the reference arm is a control arm or another active intervention. Comparisons between active interventions provide information about the effect of characteristics that differ between the arms of the trial. Therefore, these trials do not inform estimates of the intercept, trial-level indicators or time-point-level indicators. This is discussed further in our methodological paper.^10^

To account for correlations due to multi-arm and multi-follow-up trials, we specify a within-trial covariance matrix that depends on the correlation coefficient between observations at different time points. Based on observations in the data, we chose a correlation of 0.8. For further details, refer to the online supplemental material (Section C).

In our primary model, we included random effects (RE) to capture variation in intervention effects between trials. We assumed equal between-trial heterogeneity variances across interventions and follow-up times. That is, we made the usual assumption from network meta-analysis that the variation in relative intervention effects between trials is the same for different intervention comparisons.^12^ We made the additional assumption that the variation in intervention effects between trials is the same at different follow-up times, essentially treating multi-follow-up trials in the same way as multi-arm trials.

We fitted our model in a Bayesian framework using Markov chain Monte Carlo (MCMC) methods implemented in JAGS.^13^ Unless otherwise stated, we assigned uninformative prior distributions to all parameters. For the heterogeneity parameter, this deviated from our protocol in which we proposed to use an informative prior. However, we were able to include more data than expected in our final analysis and had sufficient information to estimate heterogeneity from the data. To aid convergence, we centred all indicators (including interactions) about their mean. While this does not affect the interpretation of any estimated coefficients, it means the intercept represents the effect of indicators at their mean value. Details of the implementation of our analyses can be found in Section D of the online supplemental material.

In presenting the results, we provide the estimated coefficient and 95% credible interval (CI) associated with each indicator and interaction term, along with the probability that each coefficient is less than zero, P(< 0), and the probability that it is greater than zero, P(> 0). These probabilities were calculated as the proportion of parameter samples falling either side of zero during the MCMC (after convergence of the chain).

#### Selection of characteristics and interactions

To ensure maximum statistical power, while retaining the detail of our analytic framework, we aimed to reduce the number of indicators in our model through a three-step selection process. The first two steps involved the selection of indicators, while the latter focused on selecting interactions. First, we assessed the collinearity between the different indicators and inspected pairs with absolute correlations of at least 0.5. We either discarded one of the indicators or combined them into a new indicator encapsulating information from both. In a second step, we identified indicators that received identical responses (ones or zeros) more than 80% of the time. We discarded or redefined these indicators, ensuring that we retained any deemed to be particularly important by our stakeholders (see our Patient and public involvement statement).

To select interaction terms, we used a Bayesian stochastic search variable selection (SSVS) approach based on work by Efthimiou et al.^14^ At each iteration of the MCMC, the SSVS model uses information from the data to select interactions from a prespecified set. Essentially, the model ‘shrinks’ parameter estimates in order to avoid overfitting. Interactions for which there is the most evidence of an effect are selected most often. Given the number of indicators in our model, there were almost 300 possible pairwise interaction terms. As it was infeasible to investigate all these interactions, even within the SSVS framework, we instead focused on interactions with age and behaviour targeted (diet and/or physical activity) as these were the interactions deemed most interesting by our stakeholders (see below). We applied the SSVS model in a stepwise process, fitting models with the following interaction terms: (i) no interactions, (ii) interactions between age and all other indicators and (iii) interactions with the behaviour targeted and all other indicators. In our final model, we included interactions from (ii) and (iii) that were selected more than 50% of the time. We describe details of the SSVS models in Section E of the online supplemental material.

#### Secondary and sensitivity analyses

As a secondary analysis, we performed a fixed effects (FE) model by setting the heterogeneity to zero. Although this was not pre-specified in our analysis plan, FE meta-regression analyses have a valid interpretation in the presence of unexplained heterogeneity.^15^

In addition, we performed sensitivity analyses on the different outcome scales. We fitted the model for (non-mapped) BMI and zBMI observations separately (but not for BMI percentile due to the small number of data points). Because the conversion from BMI percentile to zBMI involves fewer assumptions than the conversion from BMI, we also performed our analysis with zBMI and (mapped) BMI percentile measurements only. In our analysis protocol, we specified that we would include an indicator for whether the outcome had been mapped. Since the purpose of this is fulfilled by the sensitivity analyses and to keep the number of variables to a minimum, we chose not to include this indicator in our model.

Finally, we performed sensitivity analyses using different values for the correlation coefficient between observations at different time points. Based on observations in the data (see Section C of the online supplemental material), we investigated correlations of 0.5 and 0.95.

### Combinations of indicator values

We evaluated the predicted outcome for every possible plausible combination of values for the indicators selected for inclusion in the model. We excluded unrealistic combinations such as a time point being both medium and long term, or an intervention targeting ‘physical activity alone’ and ‘both diet and physical activity’. We also ensured that at least one intervention-level indicator for a mechanism of action (participation, education, social environment or physical environment) was non-zero. The outcome of the model is in units of mean difference in change from baseline in zBMI (intervention relative to control). Therefore, the smaller (more negative) the model outcome, the more beneficial the intervention. To identify which combination of indicator values predicts the ‘best’ results (greatest effectiveness), we searched for the instance associated with the minimum value of the outcome. In addition, we identified the indicators associated with the least beneficial (‘worst’) effect by evaluating the maximum value of the outcome. It is likely that various combinations of indicator values lead to similar predicted effects. Therefore, we also investigated the typical indicator values found in the best and worst 1% of combinations (ie, combinations that lead to the bottom and top 1% of predicted outcomes). To interpret the magnitude of effect at each combination, we converted the intercept to its equivalent value for non-centred indicators using the mean value of each indicator (see Section F of the online supplemental material for details).

### Patient and public involvement

We involved members of the public in multiple stages of the work. Two school attenders were members of the project advisory group. In the development of the analytic framework, we held two workshops involving 11 children and young people up to age 18 and two workshops involving eight schoolteachers. At these workshops, we generated ideas for inclusion in the analytic framework. The full analytic framework was later discussed in a larger meeting including one young person and one schoolteacher from these workshops. We additionally involved 35 children and young people (ages 6 to 18) in the coding of the interventions, relying on them for all coding decisions of the ‘fun factor’ item.^8^

## Results

Among the 244 trials included in the Cochrane reviews, only 208 provided data that allowed their inclusion in meta-analyses, and four of these provided data in a form that could not be converted to zBMI. Our analysis included 295 time-point observations from 250 intervention arms in the remaining 204 trials. The trials either reported zBMI data directly (171 observations from 110 trials) or provided data that could be mapped to zBMI from BMI (88 observations from 67 trials), percentiles (25 observations from 18 trials) or proportions of the intervention arm in different weight categories (11 observations from nine trials). The observations correspond to 233 comparisons between an intervention and a reference arm, some of which were observed at multiple time points. Brief characteristics of the trials included in our analysis are summarised in online supplemental table S1 (section G); further details can be found in our two Cochrane reviews.^6,7^

### Selection of indicator variables

The final indicators included in the model are listed in table 1 with a brief description of how they were coded and the results of the coding. We included 18 intervention-level, three trial-level and three time-point-level indicators. The coded characteristic ‘change of behaviour targeted’ encapsulated whether the intervention targets diet alone, physical activity alone or a combination of both; we coded this as two indicators, treating ‘diet alone’ as a reference. In Section H of the online supplemental material, we describe the results of our indicator selection process, including the correlation matrix between the intervention-level indicators (online supplemental figure S1), the proportion of indicators with identical responses (online supplemental figure S2) and which indicators were removed or redefined at each step.

**Table 1:** Description of indicators included in the final analysis

Indicator variable	Description	Coding	N (%)
**Intervention-level indicators (n=250 included arms)**			
School	Was the intervention delivered in a school (in full or in part)?	1 if Yes 0 if No	177 (70.8%) 73 (29.2%)
Home	Was the intervention delivered in the home (in full or in part) OR did it include any home activity for the child?	1 if Yes 0 if No	106 (42.4%) 144 (57.6%)
Community	Was the intervention delivered in the community or other setting (in full or in part)?	1 if Yes 0 if No	70 (28.0%) 180 (72.0%)
Individual	Was the intervention delivered to the child individually (either exclusively OR both individually and as part of a group)?	1 if Yes 0 if No	119 (47.6%) 131 (52.4%)
Electronic	Did the intervention involve any electronic component (exclusively/significantly/as a minor component)?	1 if Yes 0 if No	53 (21.2%) 197 (78.8%)
Diet and physical activity	Did the intervention aim to change both diet and activity?	1 if Yes 0 if No	137 (54.8%) 113 (45.2%)
Physical activity	Did the intervention aim to change physical activity alone?	1 if Yes 0 if No	68 (27.2%) 182 (72.8%)
Multi-strategy	Did the intervention use multiple strategies (three or more)?	1 if Yes 0 if No	161 (64.4%) 89 (35.6%)
Duration	Was the intervention long (≥30.33 weeks) or short (<30.33 weeks)?*	1 if Long 0 if Short	125 (50.0%) 125 (50.0%)
Intensity	What was the level of engagement with the children during the peak engagement period?†	1 if High 0 if Low	152 (60.8%) 98 (39.2%)
Integration	Was the intervention integrated into the normal curriculum/habits?	1 if Yes 0 if No/Partially	118 (47.2%) 132 (52.8%)
Flexibility/ choice	Was the intervention designed to be implemented in a flexible manner OR to include choice for the child?	1 if Yes 0 if No	116 (46.4%) 134 (53.6%)
Fun factor	Was the intervention considered fun?	1 if Fun 0 if Boring/ Neutral	153 (61.2%) 97 (38.8%)
Resonance	Was the intervention experienced by children via someone external or unusual?	1 if Yes 0 if No	131 (52.4%) 119 (47.6%)
Participation	Did the intervention have an explicit component of modifying the child’s behaviour?	1 if Yes 0 if No	168 (67.2%) 82 (32.8%)
Education	Did the intervention have an explicit component of education/information provision for the child?	1 if Yes 0 if No	185 (74.0%) 65 (26.0%)
Social environment	Did the intervention have an explicit component aiming to change the social environment of the child?	1 if Yes 0 if No	174 (69.6%) 76 (30.4%)
Physical environment	Did the intervention have an explicit component aiming to change the physical environment of the child?	1 if Yes 0 if No	79 (31.6%) 171 (68.4%)
**Trial-level indicators (n=204 trials)**			
Age	What was the targeted age of children in the trial (based on mean age)?	1 if 12–18 years 0 if 5–11 years	54 (26.5%) 150 (73.5%)
Income country	What is the income status of the country in which the trial was conducted according to World Bank criteria?	1 if High 0 if Non-high	175 (85.8%) 29 (14.2%)
SES	What was the socio-economic status of the participants (based on categorizations described by the trial authors)?	1 if Mixed 0 if Low	157 (77.0%) 47 (23.0%)
**Time-point-level indicators (n=261 observed time points)**			
Medium	Was the follow-up time medium-term (9 months to <15 months)?	1 if Yes 0 if No	88 (33.7%) 173 (66.3%)
Long	Was the follow-up time long-term (>15 months)?	1 if Yes 0 if No	63 (24.1%) 198 (75.9%)
ROB	Was the result at high risk of bias?	1 if High 0 if Low/Some concerns	102 (39.1%) 159 (60.9%)

* 30.33 weeks is the median duration across all interventions (including studies excluded from the primary analysis).

† As described in Reference high intensity refers to the child engaging with the intervention at least once a week, low intensity reflects engagement of less than once a week.

In table 2, we list the interactions selected by the stepwise SSVS procedure for the primary (RE) and secondary (FE) analyses. For the results of the SSVS model at each step, see online supplemental table S2 and figures S3–S8 (Section I). We summarise the results of the model without interactions further below (Section "Model without interactions").

**Table 2:** Interactions selected by the stochastic search variable selection procedure for the primary (random effects) and secondary (fixed effects) analyses

RE and FE model	RE model only	FE model only
**Interactions with age**		
Electronic	Education	Fun factor
Diet and physical activity		
Multi-strategy		
Integration		
Resonance		
Income status of country		
**Interactions with diet and physical activity**		
Electronic	Multi-strategy	School
Fun factor	Resonance	Home
Age (as above)	Risk of bias	Social
		Income status of country
**Interactions with physical activity only**		
Electronic	Fun factor	Community
Duration		Individual
Income status of country		Multi-strategy
Risk of bias		

We group the interactions into whether they were selected for both the RE and FE model, the RE model only or the FE model only.

FE, fixed effects; RE, random effects.

### Main results

#### Primary analysis (RE)

**Figure 1:**
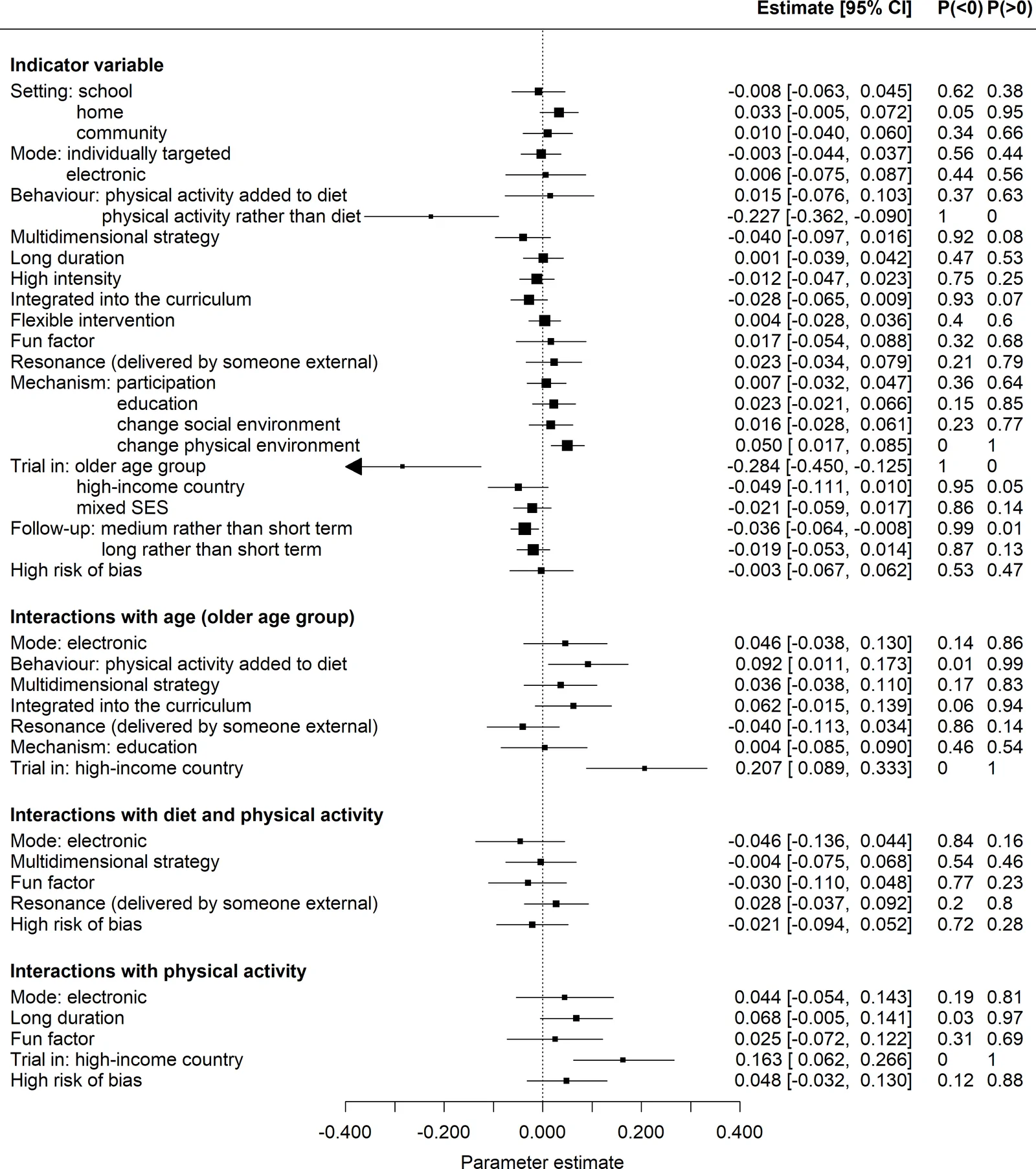
Parameter estimates from the primary (random effects) analysis. On the right, we list the estimate of each regression coefficient and its 95% credible interval (CI) along with the probability that the coefficient is less than or greater than zero, P (< 0) and P (> 0). Intervention effects were measured as mean differences in change from baseline in zBMI; therefore, coefficients of indicators that are less than zero indicate greater effectiveness.

Figure 1 shows a forest plot of the results of our primary analysis (RE model). Intervention effects were measured as mean differences (MD) in change from baseline in zBMI. Since effective interventions lead to smaller increases (or larger decreases) in zBMI, coefficients of indicators that are less than zero indicate greater effectiveness. The estimate of the model intercept and its 95% CI was –0.037 (–0.053, –0.022). This represents the effect of an intervention with all indicators set to their mean value, indicating that the interventions were beneficial on average. The heterogeneity standard deviation (and its 95% CI) was estimated to be 0.080 (0.067, 0.093), which is relatively large compared with the typical values of the estimated coefficients. In the following, we present estimates of coefficients as differences in mean differences (DMD) and their 95% CI. For a particular indicator, the DMD represents the additional effect of an intervention with indicator value 1 compared with an intervention with indicator value 0, conditional on the two interventions sharing the same values of all other indicators. Therefore, a negative DMD indicates that an intervention with that indicator value equal to one is more beneficial than an otherwise identical intervention with that indicator equal to 0. Conversely, a positive DMD implies that setting that indicator value to 1 rather than 0 makes the intervention less beneficial with respect to an otherwise identical intervention.

Our results provide strong evidence for a greater beneficial effect of interventions targeting physical activity alone compared with diet alone (–0.227 (–0.362,–0.090), P(< 0) = 1). Conversely, we observe no evidence of differing effects between diet and physical activity compared with diet alone. There is some indication of (small) greater effects for interventions that use multiple strategies (–0.040 (–0.097, 0.016), P(< 0) = 0.92) and are fully integrated within the curriculum/every day habits (–0.028 (–0.065, 0.009), P(< 0) = 0.93). On the other hand, the results indicate that interventions are less beneficial if they involve a change to the physical environment (most commonly the food environment) (0.050 (0.017, 0.085), P(> 0) = 1) and possibly less beneficial if they have some home-based component (0.033 (–0.005, 0.072), P(> 0) = 0.95).

There is strong evidence that, overall, interventions are more effective for children aged 12–18 years than for those aged 5–11 years (–0.284 (–0.450,–0.125), P(< 0) = 1). The analysis also suggests that the average intervention may work better in higher-income countries (–0.049 (–0.111, 0.010), P(< 0) = 0.95). However, both age and income status of the country appear in multiple interactions, so the interpretation of these results is more complicated. We return to this below (Section "Combinations of indicators"). The results for follow-up time indicate that, compared with short-term, larger beneficial effects are seen at medium-term (–0.036 (–0.064,–0.008), P(< 0) = 0.99), potentially followed by long-term (–0.019 (–0.23, 0.014), P(< 0) = 0.87).

Interactions are described as synergistic if the combined effect of the two indicators is greater than the sum of each independently or as antagonistic if the combined effect is less than the sum of each independently. In figure 1, interactions less than zero indicate a synergistic effect of the two indicators, whereas interactions greater than zero indicate an antagonistic effect. We find antagonistic interactions between the older age group and (i) interventions targeting both diet and physical activity and (ii) interventions conducted in high-income countries. We also observe antagonistic interactions between interventions targeting physical activity alone and (i) those conducted in high-income countries and (ii) interventions with long duration. We find no evidence of other interactions with interventions that target both diet and physical activity. We discuss the interpretation of these interactions in Section "Combinations of indicators".

#### Secondary analysis (FE)

**Figure 2:**
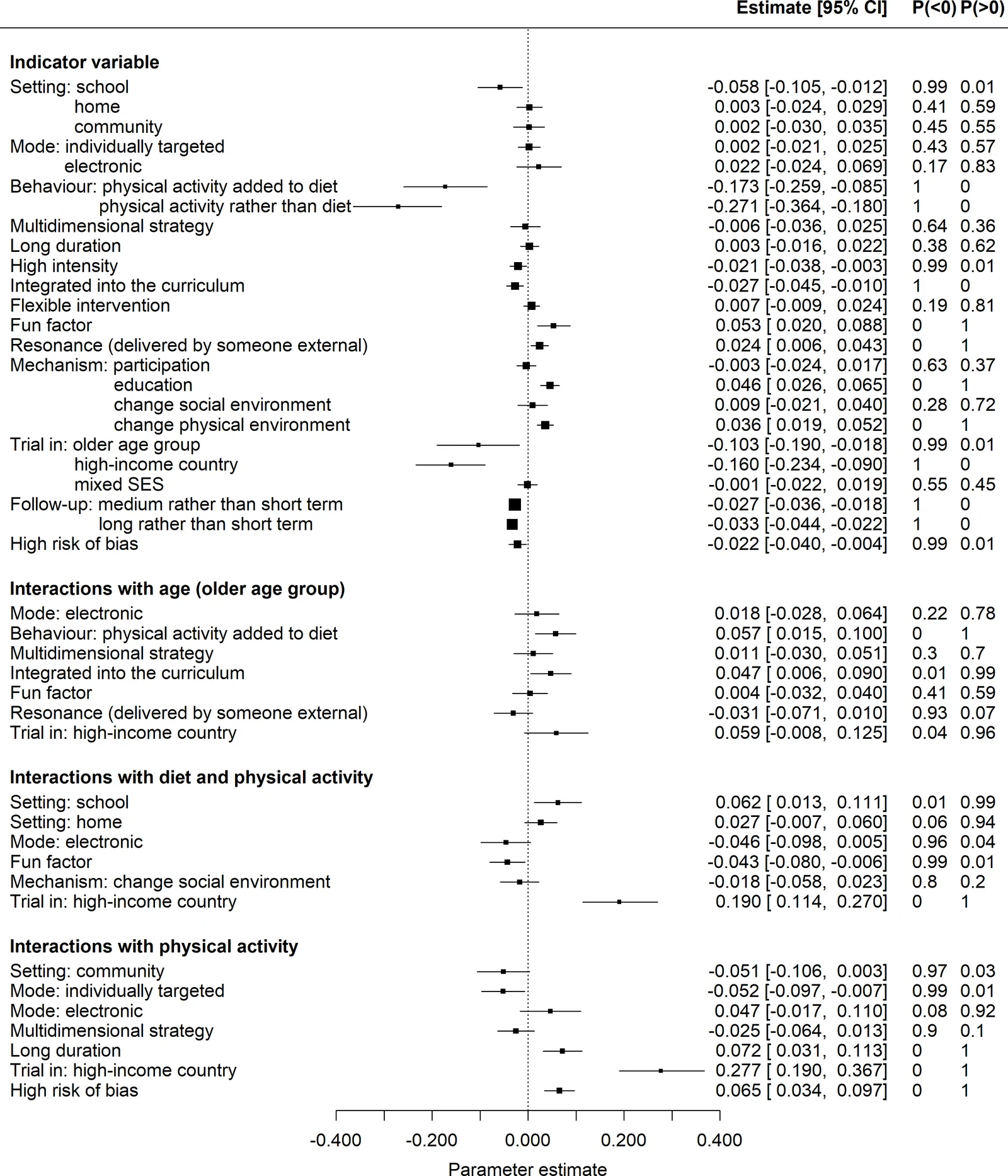
Parameter estimates from the secondary (fixed effects) analysis. On the right, we list the estimate of each regression coefficient and its 95% CI along with the probability that the coefficient is less than or greater than zero, *P* (< 0) and *P* (> 0). Intervention effects were measured as mean differences in change from baseline in zBMI; therefore, coefficients of indicators that are less than zero indicate greater effectiveness.

The results of our secondary analysis (FE model) are shown in figure 2. As expected, this analysis leads to more precise parameter estimates. In addition to the effects identified in the primary analysis, the FE model suggests beneficial effects for interventions that are based in a school, target diet and physical activity (compared with diet alone) and are high intensity. It also identifies additional, less beneficial effects for interventions that are considered fun, resonant and involve an educational component. The FE analysis provides stronger evidence (compared with RE) that the average effect of interventions is more beneficial in higher-income countries and that greater benefits are seen at long-term follow-up compared with short term. In addition, this model finds that high risk of bias is associated with larger beneficial effects.

In addition to the interactions identified by the RE analysis, the FE model identifies several other effects. For the older age group, we observe antagonistic interactions with interventions that are integrated into the curriculum, and, with lower certainty, synergistic effects with interventions delivered by someone resonant. For interventions targeting both diet and physical activity, the FE model finds strong evidence of antagonistic effects with school-based interventions and high-income countries, and weaker evidence for antagonistic effects with interventions that involve home-based activities or delivery. Conversely, we observe synergistic effects between dietary and physical activity interventions and those that are considered fun and those that involve an electronic component. For interventions that target physical activity alone, we find reasonable evidence of all interactions (probabilities ≥ 0.9). The strongest signals are for antagonistic effects with long duration, high-income countries and high risk of bias, and synergistic effects with interventions delivered in a community setting and those involving an individual-level component. We summarise differences between the results of the FE and RE models in online supplemental tables S3 and S4 (section J).

#### Combinations of indicators

By evaluating the model at every combination of indicator values, we found that it predicts the greatest effectiveness for interventions that are set in a school, have an element that is delivered individually, target physical activity alone, contain multiple strategies, are high intensity, of short duration, are delivered by someone resonant and aim to modify behaviour through participation. These findings are associated with the older age group, low-income countries, mixed (rather than low) SES and medium-term follow-up.

Since we found evidence of various interactions with age and income status, we re-evaluated the ‘best’ combination of indicator values specific to each age group in both high and non-high-income countries. Online supplemental table S5 (section K) summarises these results. For the younger age group, resonance is not associated with the largest beneficial effect, but instead, the model identifies interventions that are integrated into the curriculum or daily habits. The indicator combination which differs most from all others is for the younger age group in high-income countries. This is the subset associated with the smallest most beneficial effect. In contrast to the older age group and the younger age group in low-income countries, the results indicate that beneficial effects in this group are associated with interventions involving an electronic component, that target both diet and physical activity and that are fun.

For each subset (age and income status), the largest beneficial effect predicted by the model is for medium-term follow-up and mixed SES (rather than targeted at low SES). We re-evaluated the most beneficial indicator combination specific to low SES, long-term follow-up and short-term follow-up, respectively. In each subset, the model identified the same set of intervention indicators associated with the largest beneficial effect. In online supplemental table S5, we list the value of the model obtained in each scenario.

The results for the worst (least beneficial) combination of indicators are presented in online supplemental table S6. As expected, these indicators mostly take the opposite value to those in the best (most beneficial) combination. Indicator values that take the same value in both the best and worst combinations are highlighted; they reflect the presence of interactions involving this indicator.

As expected, many other combinations of indicators predict outcomes that are similar to the best and worst. This can be deduced from the effect estimates for individual indicators, many of which are very close to zero and are not involved in any interactions (eg, school, community, individually targeted, flexible, participation, social). Therefore, changing the value of these indicators has a minimal effect on the predicted mean difference.

Across all possible combinations, some predict outcomes that are less beneficial than a control (ie, positive mean differences). Consistent with the results for the best combination, the greatest benefits are associated with the older age group in non-high-income countries where over 99.9% of possible combinations predict beneficial effects. However, across the younger age group and the older age group in high-income countries, this is only 61%.

The investigation of the best and worst 1% of combinations provides some further insights. In non-high-income countries (regardless of age group), 100% of the best combinations involve physical activity alone. In the older age group (regardless of income status of the country), this is 94%. The best combinations in these groups also tend to be of short duration (96%). However, for the younger age group in high-income countries, we find different signals. For example, the best combinations in this group tend to favour diet and physical activity (75%), while physical activity alone appears in 99.9% of the *worst* combinations. Across all age groups and countries, the best combinations rarely involved changes to the physical environment (11%) or any home-based activity (18%), whereas both these indicators were prevalent in the worst combinations (96% and 87%, respectively).

#### Model without interactions

The parameter estimates for the RE model without interactions (fitted in the first stage of the SSVS procedure) are shown in online supplemental figure S3 (section I.1.1). Most estimates are similar to those in the primary analysis. However, without interactions, the strong beneficial effects of physical activity alone, high-income status of country and the older age group disappear. As expected, these are the variables involved in the strongest interactions found in the primary model. This highlights that the effects of these variables depend on the values of the others. For example, the model without interactions provides no evidence for an effect of physical activity (compared with diet) on average. However, the primary analysis and the tests in the previous section show that this differs depending on the age and income status of the trial populations. Online supplemental table S5 shows that physical activity alone is more beneficial in low-income countries (regardless of age) and in the older age group (regardless of income status) but is less beneficial for younger children in high-income countries. Similarly, the effect of age group depends on the income status of the country. In online supplemental table S5, we observe that the difference in predicted outcome between age groups is larger in non-high-income countries compared with high income countries.

#### Sensitivity analyses

Sensitivity analyses with different outcome scales analysed separately, that is, excluding any mapped data, are reported in full in online supplemental figures S9–S11 (section L). Results for BMI only are similar to those of the primary analysis, while the zBMI only results are largely uncertain, with wider credible intervals. We summarise the main differences between the results on different scales in online supplemental tables S7 and S8. For BMI alone, effects are greater in high-income countries, while for zBMI alone there is a greater beneficial effect in low-income countries; this could be due in part to the small amount of data available from low-income countries. For the interaction terms, we observe no conflicting evidence between the analyses on separate outcome scales. The only interaction that appears with strong evidence in one of the separate outcome analyses that does not appear in the primary analysis is a synergistic interaction between interventions with an electronic component and interventions targeting both diet and physical activity in the analysis of BMI only. In sensitivity analyses of the main RE analysis assuming different correlations between observations at different time points, we observed negligible differences from the primary analysis across all estimated effects (online supplemental figures S12 and S13 (Section M)).

## Discussion

In our re-analysis of the results of 204 randomised trials using an analytic framework co-developed with stakeholders, we found that the most effective characteristic to include in a behavioural change intervention to prevent obesity in children was physical activity. Physical activity interventions delivered in a school setting, which included active participation, were of high intensity and short duration and were delivered through multiple strategies appeared the most effective. For young children (of primary school years) living in high-income countries, greater effectiveness appeared to be possible where these interventions were also integrated into the normal school day, included a healthy diet, involved an electronic component and were ‘fun’. Although these beneficial effects are small, when delivered at scale, the effects of these preventive interventions have the potential to contribute meaningfully to a reduction in the prevalence of childhood obesity.^16^

Strengths of our investigation include our use of a large, comprehensive, updated systematic review of randomised trials; selection of indicator variables derived from a co-produced analytic framework that benefitted from the involvement of children, young people, teachers and public health professionals; careful coding of intervention and trial characteristics by a mixture of researchers and children/young people themselves; and sophisticated statistical methods. The study is not without limitations. These include our dependence on the nature of interventions that have been investigated in randomised trials, which were mainly school-based and include many that would be delivered in different ways now (eligible trials were published between 1990 and 2023). For example, the role of electronic or digital implementation of interventions could not be examined in detail. Trial reports also provided very little information on how well interventions were implemented; we did not include implementation issues in our analytic framework for this reason. We did not explore in detail the impact of participant characteristics on intervention effectiveness. Average results for whole populations can hide differences in effects between subgroups of the population, and these differences may lead to, or widen, health inequalities. It is important that attempts to prevent obesity in children ensure, as best they can, that they minimise inequity. In a parallel project, we have examined this question in detail for the trials included here.^17^

Many papers have reported the benefits of one or more of the behavioural change characteristics considered here, although few have employed a systematic approach using controlled studies. Our main finding concurs with that of a previous study, which demonstrated the effectiveness of physical activity interventions in the school setting, particularly when included in the school curriculum and emphasising participants’ enjoyment.^18^ Another study examined interventions that included diet combined with physical activity and found that effective strategies included changes in the schoolyard, in the recess rules and in the physical education classes.^19^ In an upcoming paper, we compare the findings in the current manuscript with those in non-randomised studies. This comparison is useful for understanding the impact of obesity prevention interventions in real-world practice.

We were surprised by our finding that modification of the physical environment was associated with an unfavourable impact on prevention strategies, given the general understanding that this should be useful. Most of the modifications used in the interventions in our study related to the food environment, either alone or alongside changes to the physical activity environment. This is, however, consistent with the findings of a previous study which found only two (of nine) studies employing interventions aiming to modify the food and built environments within and around schools were effective.^20^ We also observed, unexpectedly, a suggestion that the inclusion of a home activity is not useful.^21^ However, we did not assess the degree of active parental involvement, and in most of the trials, this only extended to newsletters and other educational information sent to the home of the child. In many trial reports, it was difficult to assess the level of parental involvement from the description of interventions. Therefore, we decided a priori to use our home environment and home activity codes as a proxy. A more targeted investigation into the impact of the degree of parental involvement would be a valuable topic for future research.

Much effort is invested by governments globally in childhood obesity prevention policies that address food and beverages. For example, in England, current headline actions include a soft drinks industry levy (‘sugar tax’), calorie labelling, town planning restrictions for hot food take-aways and partial banning of advertisements for less healthy products on television, with much less focus on the promotion of physical activity (a notable exception being funding for schools to support efforts in promoting physical education and engagement with sport). We suggest that even greater gains might be achievable if actions were also focused on promoting physical activity. In a similar vein, most schools have either separate policies (or programmes) around food and physical activity/sport or an overarching policy for both, and these have been found to afford relatively more attention to food compared with physical activity.^22^ Our findings are particularly relevant to those providing guidance for schools, and we encourage those responsible to ensure strategies relating to physical activity are as comprehensive as those for food.

There is increasing enthusiasm for applying ‘whole-systems’ approaches to communities, societies and schools to address childhood obesity.^23^ These highlight the importance of upstream interventions and those requiring lower individual agency as the key to success. A whole-systems approach involves multiple strategies and levels of intervention interconnected via a programme theory and logic model. Included in these are specific strategies, often school-based, which are of the type included in the two Cochrane reviews feeding into this work. We believe the findings of this work are therefore relevant to those providing guidance on, or implementing, whole-systems approaches to preventing obesity in children and young people.

Being physically active and consuming a healthy diet during childhood offer many important benefits beyond contributing to healthy weight and development, including well-being and mental health, dental health, the ability to learn and educational attainment, and realisation of full life-time potential.^4^ The findings presented in this paper should not be misinterpreted as ‘diet doesn’t matter’; it does. Our findings suggest that behavioural change interventions to prevent obesity in children should increase their focus on the promotion of physical activity and should consider the other effective characteristics we identify here.

### Dissemination to participants and related patient and public communities

The results of the study were discussed with public contributors at two meetings dedicated to this in January 2024 to help us disseminate the findings and interpret the results. The first meeting involved six children and young people. The second meeting involved four current and former teachers. In addition, one public contributor from each of these workshops participated in an expert meeting with public health professionals in February 2024, where we presented the results and invited comment.

## Supplementary material

10.1136/bmjph-2024-001707 online supplemental file 1

## Data Availability

Data are available in a public, open access repository.
