# Supplementary material for: Factors associated with the effectiveness of interventions to prevent obesity in children: a synthesis of evidence from 204 randomised trials
Source: BMJ Public Health. 2025 May 12;3(1):e001707. doi: 10.1136/bmjph-2024-001707 (PMC12086980; doi:10.1136/bmjph-2024-001707)
Supplement: online supplemental file 1 [file bmjph-3-1-s001.docx]

Supplementary material for “Factors associated with effectiveness of interventions to prevent obesity in children: a synthesis of evidence synthesis from 204 randomized trials”

Contents

[A Coding of binary intervention indicators 3](#_Toc186457514)

[B Mathematical details of the complex synthesis model 5](#_Toc186457515)

[B.1 Random effects model 5](#_Toc186457516)

[B.2 Fixed effects model 6](#_Toc186457517)

[C Imputing correlation coefficients to specify the within study covariance matrix 7](#_Toc186457518)

[D Implementation of model in JAGS 7](#_Toc186457519)

[E Selection of interactions via stepwise stochastic search variable selection (SSVS) models 8](#_Toc186457520)

[E.1 SSVS model 8](#_Toc186457521)

[E.2 Step-wise SSVS procedure 8](#_Toc186457522)

[E.3 Deviations from our analysis plan (indicator selection) 9](#_Toc186457523)

[F Adjusting the intercept 9](#_Toc186457524)

[G Study characteristics 11](#_Toc186457525)

[H Selecting indicator variables 27](#_Toc186457526)

[I Selecting interactions via SSVS 29](#_Toc186457527)

[I.1 Primary analysis (random effects) 30](#_Toc186457528)

[I.1.1 Step 1: No interactions 30](#_Toc186457529)

[I.1.2 Step 2: Interactions with age 31](#_Toc186457530)

[I.1.3 Step 3: Interactions with change in behaviour targeted 32](#_Toc186457531)

[I.2 Secondary analysis (fixed effects) 33](#_Toc186457532)

[I.2.1 Step 1: No interactions 33](#_Toc186457533)

[I.2.2 Step 2: Interactions with age 34](#_Toc186457534)

[I.2.3 Step 3: interactions with change in behaviour targeted 35](#_Toc186457535)

[J Comparing random and fixed effects models 36](#_Toc186457536)

[K Combination of indicator values 38](#_Toc186457537)

[L Sensitivity analysis: separate analyses of different outcome scales 40](#_Toc186457538)

[L.1 zBMI only 41](#_Toc186457539)

[L.2 BMI only 42](#_Toc186457540)

[L.3 zBMI and percentile only 43](#_Toc186457541)

[L.4 Comparison of different outcome scales 44](#_Toc186457542)

[M Sensitivity analysis: assuming different correlations between repeated measures over time 45](#_Toc186457543)

[M.1 Correlation of 0.5 45](#_Toc186457544)

[M.2 Correlation of 0.95 46](#_Toc186457545)

[References for supplementary materials 47](#_Toc186457546)

# Coding of binary intervention indicators

Below we list the (dichotomous) intervention indicators defined by the analytic framework and how they were coded. For details of how the questions were answered, we refer to [1]. We describe any dichotomizations applied to continuous or categorical variables and give the resulting split of the data (where we write $n$ for the number of interventions that fall into that category). The numbers refer to the full set of coded interventions ($n=255$) as reported in [1] rather than the final set of interventions included in the analysis ($n=250$, see Table 1 in the main paper).

1. School: was the intervention delivered in a school (in full or in part)?
   - = 1 if Yes $(n=180)$
   - = 0 if No $(n=75)$
2. Home: was the intervention delivered in the home (in full or in part)?
   - = 1 if Yes $(n=47)$
   - = 0 if No $(n=208)$
3. Community: was the intervention delivered in the community or other non-school/non-home setting (in full or in part)?
   - = 1 if Yes $(n=72)$
   - = 0 if No $(n=183)$
4. Home activity: did the intervention include a home activity for the child?
   - = 1 if Yes $(n=91)$
   - = 0 if No $(n=164)$
5. Group: Was the intervention delivered to the child as part of a group (in full or in part)?
   - = 1 if Yes $(n=211)$
   - = 0 if No $(n=44)$
6. Individual: Was the intervention delivered to the child individually (in full or in part)?
   - = 1 if Yes $(n=122)$
   - = 0 if No $(n=133)$
7. Electronic: was the intervention delivered to the child electronically?
   - = 1 if Yes exclusively/Yes significantly/Yes as a minor component $(n=54)$
   - = 0 if No $(n=201)$
8. Diet: Did the intervention aim to change diet?
   - = 1 if Yes exclusively/Yes substantially $(n=187)$
   - = 0 if No/Yes minimally $(n=68)$
9. Physical activity: Did the intervention aim to change physical activity?
   - = 1 if Yes exclusively/Yes substantially $(n=207)$
   - = 0 if No/Yes minimally $(n=48)$
10. Multi-strategy: Did the intervention use multiple strategies (three or more)?
    - = 1 if Yes $(n=161)$
    - = 0 if No $(n=94)$
11. Single phase: was the intervention applied in a single phase?
    - = 1 if Yes $(n=207)$
    - = 0 if No $(n=48)$
12. Continuous: was the intervention applied continuously?
    - = 1 if Yes $(n=246)$
    - = 0 if No $(n=9)$
13. Total duration: what was the total duration of the intervention?
    - = 1 if Long (longer than median total duration, $\geq$ 30.33 weeks) $(n=128)$
    - = 0 if Short (shorter than median total duration, $<$ 30.33 weeks) $(n=127)$
14. Peak duration: what was the duration of the period of peak engagement with the intervention?
    - = 1 if Long (longer than median peak duration, $\geq$ 25.98 weeks) $(n=131)$
    - = 0 if Short (shorter than median peak duration, $<$ 25.98 weeks) $(n=124)$
15. Intensity: what was the level of engagement with the children during the peak period?
    - = 1 if High $(n=152)$
    - = 0 if Low $(n=103)$
16. Integration: was the intervention integrated into the normal curriculum/ habits?
    - = 1 if Yes $(n=121)$
    - = 0 if No/Partially $(n=134)$
17. Flexibility: was the intervention designed to be implemented in a flexible manner/tailored to specific participants?
    - = 1 if Yes $(n=86)$
    - = 0 if No $(n=169)$
18. Choice: was choice for the child of physical activity/diet designed into the intervention?
    - = 1 if Yes $(n=66)$
    - = 0 if No $(n=189)$
19. Fun factor: was the intervention considered fun?
    - = 1 if Fun $(n=154)$
    - = 0 if Neutral/Not fun $(n=101)$
20. Resonance: was the intervention experienced by children via someone external or unusual?
    - = 1 if Yes $(n=134)$
    - = 0 if No $(n=121)$
21. Participation: did the intervention have an explicit component that requires the child to participate?
    - = 1 if Yes $(n=170)$
    - = 0 if No $(n=85)$
22. Education: did the intervention have an explicit component of education/information provision for the child?
    - = 1 if Yes $(n=190)$
    - = 0 if No $(n=65)$
23. Social: did the intervention have an explicit component aiming to change the social environment of the child (e.g., at school or home)?
    - = 1 if Yes $(n=175)$
    - = 0 if No $(n=80)$
24. Environment: did the intervention have an explicit component aiming to change the physical environment of the child (e.g., at school or home)?
    - = 1 if Yes $(n=79)$
    - = 0 if No $(n=176)$
25. Commercial: were commercial interests involved in the intervention/trial?
    - = 1 if Yes $(n=27)$
    - = 0 if No $(n=228)$

# Mathematical details of the complex synthesis model

## Random effects model

Each trial $i$ with $A_{i}$ arms and $T_{i}$ follow-up times provides a $T_{i}\left( A_{i}-1 \right)\times1$ vector of observations $y_{i}=\left( y_{i,1},y_{i,2},...,y_{i,T_{i}} \right)^{\top}=\left( y_{i,1}^{\left( 1 \right)},...,y_{i,1}^{\left( A_{i}-1 \right)},...,y_{i,T_{i}}^{\left( 1 \right)},...,y_{i,T_{i}}^{\left( A_{i}-1 \right)} \right)^{\top}$. Each $y_{i,t}^{\left( k \right)}$ is a mean difference in change from baseline to time $t$ between arm $k$ and some trial-specific reference arm $r$. The reference arm is either a control arm, $r=C$, or another active intervention, $r=A_{i}.$ As described in our Cochrane reviews [2, 3], we calculated mean differences from arm-level data selected in the following order of preference: (i) follow-up means adjusted for baseline values, (ii) mean change from baseline (change scores), (iii) unadjusted baseline and follow-up means, (iv) unadjusted follow-up means without baseline data. For scenario (iii) we used the baseline and follow-up values to first calculate change from baseline. To obtain standard errors on these change scores we assumed a correlation coefficient of 0.9 between baseline and follow-up based on observed correlations in other trials. This is different from the value of 0.95 we used in our Cochrane reviews which was informed by initial observations of a subset of trials.

We define a meta-regression style model assuming additive effects of indicator variables (covariates) on three levels: study ($z_{i}$), intervention ($x_{i}^{\left( k \right)}$), and follow-up time ($w_{i,t}$). We also include pairwise interaction terms ($\mathcal{J}_{i,t}^{\left( k \right)}$*)* between indicators on any level. The form of the model depends on the reference arm of the trial and is summarized as follows,

$$\boldsymbol{y}_{\boldsymbol{i}}\sim N\left( \boldsymbol{\delta}_{\boldsymbol{i}},\boldsymbol{V}_{\boldsymbol{i}} \right)$$

$$\boldsymbol{\delta}_{\boldsymbol{i}}\sim N\left( \boldsymbol{\theta}_{\boldsymbol{i}},\boldsymbol{\Sigma}_{\boldsymbol{i}} \right)$$

$$\theta_{i,t}^{\left( k \right)}=\left\{ \begin{aligned} \alpha+\sum_{j=1}^{n} \beta_{j}x_{i,j}^{\left( k \right)}+\sum_{j=1}^{p} \gamma_{j}z_{i,j}+\sum_{j=1}^{q-1} \phi_{j}w_{i,jt}+\sum_{j=1}^{l} \eta_{j}\mathcal{J}_{i,jt}^{\left( k \right)} \mathrm{for} r=C \\ \sum_{j=1}^{n} \beta_{j}{(x}_{i,j}^{\left( k \right)}-x_{i,j}^{\left( r \right)}) + \sum_{j=1}^{l} \eta_{j}\left( \mathcal{J}_{i,jt}^{\left( k \right)}-\mathcal{J}_{i,jt}^{\left( k \right)} \right) \mathrm{for} r=A_{i} \end{aligned} \right.$$

$$\boldsymbol{V}_{\boldsymbol{i}}=\left( \begin{matrix} \boldsymbol{V}_{\boldsymbol{i,1}} & \boldsymbol{V}_{\boldsymbol{i,12}} & \boldsymbol{\cdots} & \boldsymbol{V}_{\boldsymbol{i,1}\boldsymbol{T}_{\boldsymbol{i}}} \\ \boldsymbol{V}_{\boldsymbol{i,21}} & \boldsymbol{V}_{\boldsymbol{i,2}} & \boldsymbol{\cdots} & \boldsymbol{V}_{\boldsymbol{i,2}\boldsymbol{T}_{\boldsymbol{i}}} \\ \boldsymbol{\vdots} & \boldsymbol{\vdots} & \boldsymbol{\ddots} & \boldsymbol{\vdots} \\ \boldsymbol{V}_{\boldsymbol{i,}\boldsymbol{T}_{\boldsymbol{i}}\boldsymbol{1}} & \boldsymbol{V}_{\boldsymbol{i,}\boldsymbol{T}_{\boldsymbol{i}}\boldsymbol{2}} & \boldsymbol{\cdots} & \boldsymbol{V}_{\boldsymbol{i,}\boldsymbol{T}_{\boldsymbol{i}}} \end{matrix} \right), \boldsymbol{\Sigma}_{\boldsymbol{i}}=\left( \begin{matrix} \tau^{2} & \tau^{2}/2 & \cdots& \tau^{2}/2 \\ \tau^{2}/2 & \tau^{2} & \cdots& \tau^{2}/2 \\ \vdots& \vdots& \ddots& \vdots\\ \tau^{2}/2 & \tau^{2}/2 & \cdots& \tau^{2} \end{matrix} \right)$$

( 1 )

where the parameters $\beta=\left( \beta_{1},...,\beta_{n} \right)^{\top}$, $\gamma=\left( \gamma_{1},...,\gamma_{p} \right)^{\top}$*,* and $\phi=\left( \phi_{1},...,\phi_{q-1} \right)^{\top}$ are the regression coefficients for the intervention, study-level, and follow-up time indicators respectively. The parameters $\eta=\left( \eta_{1},...,\eta_{l} \right)^{\top}$ are the regression coefficients for the interaction parameters.

The $T_{i}\left( A_{i}-1 \right)\times T_{i}\left( A_{i}-1 \right)$ covariance matrix $\boldsymbol{V}_{\boldsymbol{i}}$ captures correlations between measurements made in the same trial (due to multiple arms and/or follow-up times) and is assumed to be known. The diagonal elements of the covariance matrix are equal to the observed variance (squared standard error) of the measurements $y_{i,t}^{\left( k \right)}$. The off-diagonal elements capture correlations between multiple observations in the same trial and depend on two (imputed) correlation coefficients $\rho_{y,tt^{'}}$ and $\rho_{d,tt^{'}}$ (described in Section C). We refer to our development paper [4] for details of this matrix.

For the random effects model, we assume the trial-specific effects $\delta_{i,t}^{\left( k \right)}$ are normally distributed with covariance matrix $\boldsymbol{\Sigma}_{\boldsymbol{i}}$ (dimensions $T_{i}\left( A_{i}-1 \right)\times T_{i}\left( A_{i}-1 \right)$). This matrix depends on the heterogeneity variance, $\tau^{2}$, which we assume captures the between-trial variance for all arms and all follow-up times.

## Fixed effects model

As our secondary analysis we define a fixed effects model by setting $\tau=0$in Equation ( 1 ). This yields

$$\boldsymbol{y}_{\boldsymbol{i}}\sim N\left( \boldsymbol{\theta}_{\boldsymbol{i}},\boldsymbol{V}_{\boldsymbol{i}} \right)$$

$$\theta_{i,t}^{\left( k \right)}=\left\{ \begin{aligned} \alpha+\sum_{j=1}^{n} \beta_{j}x_{i,j}^{\left( k \right)}+\sum_{j=1}^{p} \gamma_{j}z_{i,j}+\sum_{j=1}^{q-1} \phi_{j}w_{i,jt}+\sum_{j=1}^{l} \eta_{j}\mathcal{J}_{i,jt}^{\left( k \right)} \mathrm{for} r=C \\ \sum_{j=1}^{n} \beta_{j}{(x}_{i,j}^{\left( k \right)}-x_{i,j}^{\left( r \right)}) + \sum_{j=1}^{l} \eta_{j}\left( \mathcal{J}_{i,jt}^{\left( k \right)}-\mathcal{J}_{i,jt}^{\left( k \right)} \right) \mathrm{for} r=A_{i} \end{aligned} \right.$$

$$\boldsymbol{V}_{\boldsymbol{i}}=\left( \begin{matrix} \boldsymbol{V}_{\boldsymbol{i,1}} & \boldsymbol{V}_{\boldsymbol{i,12}} & \boldsymbol{\cdots} & \boldsymbol{V}_{\boldsymbol{i,1}\boldsymbol{T}_{\boldsymbol{i}}} \\ \boldsymbol{V}_{\boldsymbol{i,21}} & \boldsymbol{V}_{\boldsymbol{i,2}} & \boldsymbol{\cdots} & \boldsymbol{V}_{\boldsymbol{i,2}\boldsymbol{T}_{\boldsymbol{i}}} \\ \boldsymbol{\vdots} & \boldsymbol{\vdots} & \boldsymbol{\ddots} & \boldsymbol{\vdots} \\ \boldsymbol{V}_{\boldsymbol{i,}\boldsymbol{T}_{\boldsymbol{i}}\boldsymbol{1}} & \boldsymbol{V}_{\boldsymbol{i,}\boldsymbol{T}_{\boldsymbol{i}}\boldsymbol{2}} & \boldsymbol{\cdots} & \boldsymbol{V}_{\boldsymbol{i,}\boldsymbol{T}_{\boldsymbol{i}}} \end{matrix} \right).$$

( 2 )

# Imputing correlation coefficients to specify the within study covariance matrix

As described in Section B, the within-study covariance matrix in our model depends on two correlation coefficients $\rho_{y,tt^{'}}$ and $\rho_{d,tt^{'}}$. The former is the correlation between observations of mean difference for a given intervention (relative to the reference arm) at time points$t$ and $t'$, assumed to be common to all arms. The latter is the correlation between observations of the change score in the reference arm between $t$ and $t'$.

To specify the within-study covariance matrix, we imputed sensible values for the correlations $\rho_{y,tt'}$ and $\rho_{d,tt'}$ based on observed correlations in the data. Using multi-follow-up trials, we plotted mean differences and change scores in control arms for different combinations of time points: (i) short vs medium term, (ii) short vs long term, and (iii) medium vs long term. We then calculated correlation coefficients for each plot.

To simplify the imputation we assumed that time points with one degree of separation, (i) and (iii), have the same correlation, $\rho_{1}$. We also assumed that correlations act multiplicatively across time such that time points with two degrees of separation, (ii), have the square of this correlation, $\rho_{2}=\rho_{1}^{2}$.

For mean differences, $\rho_{y,tt'}$, we found a correlation of $\rho_{1}=0.77$ for short vs medium term (based on $m=31$ data points), $\rho_{2}$ $=0.83$ ( $\rho_{1}=\sqrt{\rho_{2}}=0.91$) for short vs long term ($m=13$), and $\rho_{1}=0.86$ for medium vs long term ($m=45$). This gives an overall weighted mean of $\rho_{1}= 0.82$.

For change scores, $\rho_{d,tt'}$, we found a correlation of $\rho_{1}=0.69$ for short vs medium term ($m=24$), $\rho_{2}=0.77$ ( $\rho_{1}=\sqrt{\rho_{2}}=0.88$) for short vs long term ($m=9$), and $\rho_{1}=0.84$ for medium vs long term ($m=34$). This gives an overall weighted mean of $\rho_{1}= 0.77$.

Therefore, we chose to use $\rho_{1}=0.8$ for both mean differences and change scores with one degree of separation. To cover the range of correlations observed, we chose to perform sensitivity analyses with $\rho_{1}=0.5$ and $\rho_{1}=0.95$.

# Implementation of model in JAGS

We implemented our models in a Bayesian framework using JAGS [5]. We used four chains and assessed convergence by inspecting MCMC trace plots and using the Brooks-Gelman-Rubin $\hat{R}$ statistic [6, 7]. For all non SSVS models (see Section E) we assigned non-informative prior distributions to all parameters. For heterogeneity, we specified $\tau\sim\mathrm{Unif}(0,5)$. For the intercept and all regression coefficients we used $N(0,{100}^{2})$. An upper limit of 5 for heterogeneity and a standard deviation of 100 is large on the zBMI scale (which follows a standard normal in the general population). We used an adaptive phase of 10,000 iterations, a burn in of an additional 40,000, and a further 30,000 iterations from which we drew our posterior samples.

All code required to implement our analysis is provided in the GitHub repository here: <https://github.com/AnnieDavies/Obesity_Synthesis>

# Selection of interactions via stepwise stochastic search variable selection (SSVS) models

## SSVS model

To select interaction terms $\mathcal{J}_{i,jt}^{\left( k \right)}$, we use a Bayesian stochastic search variable selection (SSVS) model from Efthimiou et al [8]. While fitting the model, interactions are selected at each iteration of the MCMC using indicator variables $I_{j}\in\{0,1\}$ in the prior distributions of the interaction coefficients $\eta_{j}$. The priors are defined as the sum of two normal distributions. The value of the indicator variable at each iteration specifies which of these distributions to use. The first is very narrowly distributed around zero and is used when $I_{j}=0$ (the $j^{th}$ interaction term is not selected). The second is wider, allowing for non-zero values of the coefficient and is used when $I_{j}=1$ (the $j^{th}$ interaction is selected). By inspecting the posterior distribution of the indicator parameter for each interaction term $j$ we can assess the proportion of times the interaction was selected. The more important an interaction, the more often it will be selected.

The SSVS version of our model is defined in the same way as in Section B but with the following prior distributions on the interaction coefficients,

$$\pi\left( \eta_{j} | I_{j} \right)=\left( 1-I_{j} \right)N\left( 0,\xi^{2} \right)+I_{j}N\left( 0,g^{2}\xi^{2} \right)$$

$$I_{j}\sim\mathrm{Bernoulli}\left( p_{j} \right), \xi\sim N\left( 0,v_{\xi} \right),$$

$$v_{\xi}=small variance, g = large number,$$

( 3 )

where $j=1,...,l$and $l$ is the number of interactions from which to select. For all other model parameters we use the non-informative prior distributions described in Section D. The variance $v_{\xi}$, which specifies the width of the narrow distribution, should be such that it only allows for effects that are `practically zero' relative to the scale of the data. The parameter $g$, which specifies the width of the wider distribution, should be large enough to allow for non-zero (but sensible) values of the parameter. Informed by [8], the scale of zBMI, and preliminary tests on our data, we chose $v_{\xi}={10}^{-3}$ and $g^{2}=100$.

In Equation ( 3 ), the indicator variables are assigned Bernoulli prior distributions with probabilities $p_{j}$. These probabilities can be used to define informative priors based on our beliefs about the importance of interaction terms. Otherwise, setting $p_{j}=0.5 \forall j$ defines non-informative (equi-probable) priors which choose the interactions based on information from the data alone. In our step-wise procedure described in Section E.2 we use the latter.

## Step-wise SSVS procedure

As described in the main paper, we selected interaction terms for our model by applying the SSVS method in a step-wise process. Each step involved a different set of interaction terms from which to select. In the following we list the model specified at each step and give the implementation details used to fit it in JAGS [5].

1. Step 1: Model with no interactions
   - We used an adaptive phase of $n_{\text{adapt}}=10,000$ iterations, an additional burn in of $n_{\text{burn}}=20,000$, followed by a further $n_{\text{samp}}=30,000$ iterations from which we drew our posterior samples.
2. Step 2: Model with interactions between age and all other indicator variables
   - Implementation details: $n_{\text{adapt}}=10,000$, $n_{\text{burn}}=20,000$, $n_{\text{samp}}=30,000$.
3. Step 3: Model with interactions between ‘change of behaviour targeted’ (diet and/or physical activity) and all other indicators
   - As described in Section 3.1 of the main paper, behaviour targeted was coded as two binary variables indicating whether the intervention targeted physical activity alone or a combination of diet and physical activity. An intervention targeting diet alone is then indicated by setting both these variables to zero.
   - This model included interactions between each of these variables (physical activity alone and diet & physical activity) and every other indicator.
   - Implementation details: $n_{\text{adapt}}=10,000$, $n_{\text{burn}}=40,000$, $n_{\text{samp}}=30,000$.

To define our final model, we chose interaction terms that were selected more than 50% of the time in steps 2 and 3. We performed steps 1-3 separately for the random effects and fixed effect models.

## Deviations from our analysis plan (indicator selection)

In our analysis plan we set out to use the SSVS method to select intervention indicators as well as interactions. However, the first two steps of our selection process (assessing collinearity and the proportion of identical responses) led to a manageable number of indicators (see Table 1 in the main paper) all of which were deemed important by our stakeholders. Therefore, we did not require the SSVS method to reduce the number of indicators further.

Our analysis plan also states that we would to assign informative probabilities to the SSVS prior distributions, describing the interactions that our stakeholders considered most and least probable. However, beyond expressing interest in interactions between age and behaviour targeted, our stakeholders encountered challenges in pinpointing particular interactions of interest. Additionally, preliminary analyses revealed that the use of informative priors led to the dominance of predetermined interactions, undermining our objective to select interactions based on the data.

# Adjusting the intercept

In Section 3.2.3 of the main manuscript we evaluated our primary model for every possible combination of indicator variables. Each combination involved inserting values of 0 or 1 for each indicator. However, the model intercept was calculated using indicators centred at their mean value. Therefore, to interpret the outcome of the model as a mean difference in change from baseline in zBMI we need to convert the intercept from the centred model to the intercept for the non-centred model.

Using the notation from Section B, the fixed effects of the non-centred ($nc$) model (for control comparison studies) is

$$\theta_{i,t}^{\left( k \right)}=\alpha_{nc}+\sum_{j=1}^{n} \beta_{j}x_{i,j}^{\left( k \right)}+\sum_{j=1}^{p} \gamma_{j}z_{i,j}+\sum_{j=1}^{q-1} \phi_{j}w_{i,jt}+\sum_{j=1}^{l} \eta_{j}\mathcal{J}_{i,jt}^{\left( k \right)}.$$

( 4 )

The equivalent model with centred ($c$) indicators is

$$\theta_{i,t}^{\left( k \right)}=\alpha_{c}+\sum_{j=1}^{n} \beta_{j}(x_{i,j}^{\left( k \right)}-\left\langle x_{j} \right\rangle)+\sum_{j=1}^{p} \gamma_{j}(z_{i,j}-\left\langle z_{j} \right\rangle)+\sum_{j=1}^{q-1} \phi_{j}{(w}_{i,jt}-\left\langle w_{j} \right\rangle)+\sum_{j=1}^{l} \eta_{j}(\mathcal{J}_{i,jt}^{\left( k \right)}-\left\langle\mathcal{J}_{j} \right\rangle),$$

( 5 )

where the angular brackets $\left\langle. \right\rangle$ denote the mean value of that indicator (or interaction) over all observations in the data set. The intercept reported in the main paper is an estimate of the centred intercept, $\alpha_{c}$. To convert this to the non-centred intercep*t* $\alpha_{nc}$ we equate right hand side of Equations ( 4 ) and ( 5 ), which leads to

$$\alpha_{nc}=\alpha_{c}-\left( \sum_{j=1}^{n} \beta_{j}\left\langle x_{j} \right\rangle+\sum_{j=1}^{p} \gamma_{j}\left\langle z_{j} \right\rangle+\sum_{j=1}^{q-1} \phi_{j}\left\langle w_{j} \right\rangle+\sum_{j=1}^{l} \eta_{j}\left\langle\mathcal{J}_{j} \right\rangle\right).$$

( 6 )

To obtain the predicted mean differences in Table 2 in the main paper, we calculated the intercept from Equation ( 6 ) using the mean values of each indicator variable used to centre the data, and the estimated intercept ($\alpha_{c}$) and regression coefficients from our primary analysis. This gave $\alpha_{nc}=-0.0003$, which represents the effect of an intervention whose indicators are all equal to zero.

# Study characteristics

**Table S1.** Summary of the characteristics of the trials included in our analyses. For studies that provide data on multiple outcomes, we selected data in the following order of preference: (i) zBMI, (ii) percentile (mapped), (iii) BMI (mapped). The BMI-only sensitivity analysis included all studies that provided data on BMI.
*Studies that only provide contrast-level data on BMI and could not be mapped to zBMI. These studies were excluded from the main analysis but were included in the BMI-only sensitivity analysis.
**Study that only provides contrast-level data on percentile and could not be mapped to zBMI. This study was excluded from all analyses.

| **Study** | **Age group** | **Income status of country** | **Socio-economic status** | **Comparison(s)** | **Participants at baseline** | **Outcome** | **Follow-up time(s)** | **Risk of bias judgement(s)** |
| --- | --- | --- | --- | --- | --- | --- | --- | --- |
| Adab 2018 | 5-11 | High | Low | Diet and physical activity vs control | Intervention(s): 660 Control: 732 | zBMI | Long term | Some concerns |
| Amaro 2006 | 12-18 | High | Mixed | Diet vs control | Intervention(s): 153 Control: 88 | zBMI | Short term | Some concerns |
| Andrade 2014 | 12-18 | Non-High | Mixed | Diet and physical activity vs control | Intervention(s): 687 Control: 692 | zBMI BMI | Long term | Some concerns |
| Annesi 2016 | 5-11 | High | Mixed | Diet and physical activity vs control | Intervention(s): 72 Control: 42 | BMI Percentile | Short term (BMI only) Medium term | High risk |
| Annesi 2017 | 5-11 | High | Mixed | Diet and physical activity vs control | Intervention(s): 86 Control: 55 | BMI | Short term Medium term | High risk |
| Arlinghaus 2021 | 12-18 | High | Low | Physical activity vs control | Intervention(s): 87 Control: 45 | zBMI | Short term | High risk |
| Baranowski 2003 | 5-11 | High | Mixed | Diet and physical activity vs control | Intervention(s): 19 Control: 16 | BMI | Short term | Some concerns |
| Baranowski 2011 | 5-11 | High | Mixed | Diet and physical activity vs control | Intervention(s): 103 Control: 50 | zBMI Percentile | Short term | High risk |
| Barbeau 2007 | 5-11 | High | Mixed | Physical activity vs control | Intervention(s): 118 Control: 83 | BMI | Medium term | High risk |
| Barnes 2015 | 5-11 | High | Mixed | Physical activity vs control | Intervention(s): 25 Control: 23 | zBMI BMI | Short term | Some concerns |
| Barnes 2021 | 5-11 | High | Low | Diet vs Physical activity vs Diet and physical activity vs control | Intervention(s): SWAP IT: 283; Physically Active children in Education (PACE): 163; SWAP IT + PACE combined: 202 Control: 167 | zBMI BMI | Medium term | Some concerns |
| Bayne-Smith 2004 | 12-18 | High | Mixed | Diet and physical activity vs control | Intervention(s): 310 Control: 132 | BMI | Short term | High risk |
| Beech 2003 | 5-11 | High | Mixed | Diet and physical activity vs control | Intervention(s): 21 Control: 18 | BMI | Short term | Some concerns |
| Black 2010 | 12-18 | High | Low | Diet and physical activity vs control | Intervention(s): 121 Control: 114 | zBMI | Medium term Long term | Some concerns |
| Bogart 2016 | 12-18 | High | Low | Diet and physical activity vs control | Intervention(s): 829 Control: 539 | Percentile | Long term | High risk |
| Bohnert 2013 | 5-11 | High | Low | Diet and physical activity vs control | Intervention(s): 52 Control: 24 | zBMI | Short term | High risk |
| Bonsergent 2013 | 12-18 | High | Mixed | Diet and physical activity (three groups) vs control | Intervention(s): Education: 1949; Environment: 1728; Screening and care: 1687 Control: 1589 | zBMI BMI | Long term | Some concerns |
| Brandstetter 2012 | 5-11 | High | Mixed | Diet and physical activity vs control | Intervention(s): 450 Control: 495 | BMI | Long term | Some concerns |
| Branscum 2013 | 5-11 | High | Mixed | Diet and physical activity vs Diet and physical activity | Intervention(s): Comics for Health: 36; Knowledge based intervention: 34 | Percentile | Short term | High risk |
| Breheny 2020 | 5-11 | High | Low | Physical activity vs control | Intervention(s): 1153 Control: 1127 | zBMI | Short term Medium term | Some concerns |
| Brito Beck da Silva 2019 | 12-18 | Non-High | Mixed | Diet and physical activity vs control | Intervention(s): 425 Control: 457 | BMI | Medium term | Some concerns |
| Brown 2013 | 5-11 | High | Low | Diet and physical activity vs control | Intervention(s): 31 Control: 32 | zBMI BMI Percentile | Short term | Some concerns |
| Caballero 2003 | 5-11 | High | Low | Diet and physical activity vs control | Intervention(s): 879 Control: 825 | BMI | Long term | Some concerns |
| Cao 2015 | 5-11 | Non-High | Mixed | Diet and physical activity vs control | Intervention(s): 965 Control: 889 | zBMI | Medium term Long term | Some concerns |
| Chai 2019 | 5-11 | High | Mixed | Diet (two groups) vs control | Intervention(s): Telehealth: 16; Telehealth + SMS: 15  Control: 15 | zBMI BMI | Short term | High risk |
| Chen 2010 | 5-11 | High | Mixed | Diet and physical activity vs control | Intervention(s): 35 Control: 32 | BMI | Short term | High risk |
| Chen 2011 | 12-18 | High | Mixed | Diet and physical activity vs control | Intervention(s): 27 Control: 27 | BMI | Short term | Some concerns |
| Choo 2020 | 5-11 | High | Mixed | Diet and physical activity vs control | Intervention(s): 49 Control: 55 | zBMI | Short term | High risk |
| Clemes 2020 | 5-11 | High | Low | Physical activity vs control | Intervention(s): 84 Control: 90 | BMI | Short term | Low risk |
| Coleman 2012 | 5-11 | High | Low | Diet vs control | Intervention(s): 279 Control: 300 | Proportion | Medium term Long term | High risk |
| Crespo 2012 | 5-11 | High | Mixed | Diet and physical activity (three groups) vs control | Intervention(s): Family/Home + School/Community: 163; Family/Home: 195; School/Community: 216 Control: 223 | zBMI Percentile | Medium term Long term | Some concerns |
| Cunha 2013* | 5-11 | Non-High | Low | Diet vs control | Intervention(s): 242 Control: 236 | BMI | Medium term | Some concerns |
| Damsgaard 2014 | 5-11 | High | Mixed | Diet vs control | Intervention(s): 412 Control: 411 | zBMI | Short term | Some concerns |
| Davis 2021 | 5-11 | High | Low | Diet vs control | Intervention(s): 1412 Control: 1723 | zBMI BMI Percentile | Medium term | High risk |
| De Bock 2013 | 5-11 | High | Mixed | Physical activity vs control | Intervention(s): 363 Control: 328 | BMI | Short term Medium term | High risk |
| de Greeff 2016 | 5-11 | High | Mixed | Physical activity vs control | Intervention(s): 181 Control: 195 | BMI | Short term | Some concerns |
| De Heer 2011 | 5-11 | High | Low | Diet and physical activity vs control | Intervention(s): 242 Control: 326 | BMI Percentile | Short term | Some concerns |
| de Ruyter 2012 | 5-11 | High | Mixed | Diet vs control | Intervention(s): 319 Control: 322 | zBMI | Short term Medium term Long term | Low risk (short term) Some concerns (medium and long term) |
| Dewar 2013 | 12-18 | High | Low | Diet and physical activity vs control | Intervention(s): 178 Control: 179 | zBMI BMI | Medium term Long term | Some concerns |
| Diaz-Castro 2021 | 5-11 | High | Mixed | Physical activity vs control | Intervention(s): 52 Control: 51 | zBMI BMI | Short term | Some concerns |
| Donnelly 2009 | 5-11 | High | Mixed | Physical activity vs control | Intervention(s): 814 Control: 713 | BMI | Long term | High risk |
| Drummy 2016 | 5-11 | High | Mixed | Physical activity vs control | Intervention(s): 54 Control: 53 | BMI | Short term | High risk |
| Duncan 2019 | 5-11 | High | Mixed | Diet and physical activity vs control | Intervention(s): 346 Control: 329 | BMI | Short term | Some concerns |
| Dunker 2018 | 12-18 | Non-High | Mixed | Diet and physical activity vs control | Intervention(s): 131 Control: 139 | BMI | Short term | Some concerns |
| Ebbeling 2006 | 12-18 | High | Mixed | Diet vs control | Intervention(s): 53 Control: 50 | BMI | Short term | Some concerns |
| El Ansari 2010 | 12-18 | Non-High | Mixed | Physical activity vs control | Intervention(s): 80 Control: 80 | BMI | Short term | Some concerns |
| Elder 2014 | 5-11 | High | Mixed | Diet and physical activity vs control | Intervention(s): 271 Control: 267 | zBMI BMI Percentile | Medium term Long term | Some concerns |
| Ezendam 2012 | 12-18 | High | Mixed | Diet and physical activity vs control | Intervention(s): 440 Control: 376 | BMI | Long term | Some concerns |
| Fairclough 2013 | 5-11 | High | Low | Diet and physical activity vs control | Intervention(s): 138 Control: 127 | zBMI BMI | Short term | High risk |
| Farmer 2017 | 5-11 | High | Mixed | Physical activity vs control | Intervention(s): 374 Control: 369 | zBMI BMI | Medium term Long term | Some concerns |
| Ford 2013 | 5-11 | High | Mixed | Physical activity vs control | Intervention(s): 77 Control: 75 | BMI | Short term | High risk |
| Foster 2008 | 5-11 | High | Low | Diet and physical activity vs control | Intervention(s): 479 Control: 364 | zBMI BMI | Long term | High risk |
| French 2011 | 12-18 | High | Mixed | Diet and physical activity vs control | Intervention(s): 38 Control: 38 | zBMI | Medium term | Some concerns |
| Fulkerson 2010 | 5-11 | High | Mixed | Diet vs control | Intervention(s): 22 Control: 22 | zBMI Percentile | Short term | Some concerns |
| Fulkerson 2015 | 5-11 | High | Mixed | Diet vs control | Intervention(s): 74 Control: 75 | zBMI | Medium term Long term | Some concerns |
| Fulkerson 2022 | 5-11 | High | Mixed | Diet and physical activity vs control | Intervention(s): 56 Control: 46 | zBMI | Medium term | High risk |
| Gentile 2009 | 5-11 | High | Mixed | Diet and physical activity vs control | Intervention(s): 642 Control: 640 | BMI | Short term Medium term | High risk |
| Greve 2015* | 5-11 | High | Mixed | Diet and physical activity vs control | Intervention(s): 6460 Control: 6459 | BMI | Long term | High risk |
| Griffin 2019 | 5-11 | High | Mixed | Diet and physical activity vs control | Intervention(s): 42 Control: 19 | zBMI | Short term | High risk |
| Grydeland 2014 | 5-11 | High | Mixed | Diet and physical activity vs control | Intervention(s): 465 Control: 859 | zBMI BMI | Long term | High risk |
| Gustafson 2019 | 12-18 | High | Mixed | Diet vs control | Intervention(s): 277 Control: 134 | Percentile | Short term | High risk |
| Ha 2021 | 5-11 | Non-High | Mixed | Physical activity vs control | Intervention(s): 83 Control: 77 | BMI | Short term Medium term | Some concerns |
| Habib-Mourad 2014 | 5-11 | Non-High | Mixed | Diet and physical activity vs control | Intervention(s): 193 Control: 181 | BMI | Short term | Some concerns |
| Habib-Mourad 2020 | 5-11 | Non-High | Mixed | Diet and physical activity vs control | Intervention(s): 698 Control: 541 | zBMI | Long term | High risk |
| Haerens 2006 | 12-18 | High | Mixed | Diet and physical activity vs control | Intervention(s): 1116 Control: 671 | zBMI BMI | Medium term Long term | Some concerns (medium term) High risk (long term) |
| Haire-Joshu 2010 | 5-11 | High | Mixed | Diet and physical activity vs control | Intervention(s): 154 Control: 69 | zBMI | Short term | High risk |
| Han 2006 | 5-11 | Non-High | Mixed | Diet vs control | Intervention(s): 1333 Control: 1345 | Proportion | Long term | High risk |
| Hannon 2018 | 5-11 | High | Mixed | Diet and physical activity vs Diet and physical activity | Intervention(s): Intervention 1: 72; Intervention 2: 82 | Percentile | Short term Medium term | High risk (short term) Some concerns (medium term) |
| Harrington 2018 | 12-18 | High | Mixed | Physical activity vs control | Intervention(s): 867 Control: 885 | zBMI | Short term Medium term | Some concerns |
| HEALTHY Study Group 2010 | 5-11 | High | Low | Diet and physical activity vs control | Intervention(s): 2307 Control: 2296 | zBMI | Long term | Some concerns |
| Hendrie 2011 | 5-11 | High | Mixed | Diet vs control | Intervention(s): 76 Control: 69 | zBMI BMI | Short term | Some concerns |
| Hendy 2011 | 5-11 | High | Mixed | Diet and physical activity vs control | Intervention(s): 102 Control: 98 | Percentile | Short term | Some concerns |
| Hollis 2016 | 12-18 | High | Low | Physical activity vs control | Intervention(s): 645 Control: 505 | zBMI BMI | Medium term Long term | Low risk |
| Hopper 2005 | 5-11 | High | Mixed | Diet and physical activity vs control | Intervention(s): 142 Control: 96 | BMI | Short term | High risk |
| Hovell 2018 | 12-18 | High | Mixed | Diet and physical activity vs control | Intervention(s): 332 Control: 361 | zBMI | Long term | High risk |
| Howe 2011 | 5-11 | High | Mixed | Physical activity vs control | Intervention(s): 62 Control: 44 | BMI | Medium term | Some concerns |
| Hull 2018 | 5-11 | High | Mixed | Diet and physical activity vs control | Intervention(s): 162 Control: 157 | zBMI BMI | Short term Long term | High risk |
| Ickovics 2019 | 5-11 | High | Low | Diet vs Physical activity vs Diet and physical activity vs control | Intervention(s): Nutrition: 152; Physical activity: 178; Nutrition and physical activity: 152 Control: 113 | Percentile | Long term | High risk |
| Isensee 2018 | 12-18 | High | Mixed | Physical activity vs control | Intervention(s): 649 Control: 371 | Percentile | Medium term | High risk |
| Jago 2006 | 12-18 | High | Mixed | Physical activity vs Diet | Intervention(s): Physical activity: 227 Diet: 224 | BMI Percentile | Short term | High risk |
| James 2004 | 5-11 | High | Mixed | Diet vs control | Intervention(s): 311 Control: 304 | zBMI BMI | Medium term Long term | Some concerns |
| Jansen 2011 | 5-11 | High | Low | Diet and physical activity vs control | Intervention(s): 1240 Control: 1382 | BMI | Short term | High risk |
| Jones 2015 | 5-11 | High | Mixed | Physical activity vs control | Intervention(s): 19 Control: 18 | zBMI BMI | Short term Medium term | Some concerns |
| Kain 2014 | 5-11 | High | Low | Diet and physical activity vs control | Intervention(s): 651 Control: 823 | zBMI BMI | Medium term | High risk |
| Keller 2009 | 5-11 | High | Mixed | Diet and physical activity vs control | Intervention(s): 180 Control: 185 | zBMI | Medium term | High risk |
| Kennedy 2018 | 12-18 | High | Mixed | Physical activity vs control | Intervention(s): 348 Control: 252 | zBMI BMI | Short term Medium term | Low risk |
| Keshani 2016 | 5-11 | Non-High | Mixed | Diet vs control | Intervention(s): 83 Control: 88 | BMI | Medium term | High risk |
| Ketelhut 2022 | 5-11 | High | Low | Physical activity vs control | Intervention(s): 18 Control: 16 | BMI | Short term | High risk |
| Khan 2014 | 5-11 | High | Mixed | Physical activity vs control | Intervention(s): 64 Control: 68 | zBMI BMI | Medium term | High risk |
| Kipping 2008* | 5-11 | High | Mixed | Diet and physical activity vs control | Intervention(s): 249 Control: 223 | BMI | Short term | High risk |
| Kipping 2014 | 5-11 | High | Mixed | Diet and physical activity vs control | Intervention(s): 889 Control: 953 | zBMI | Short term Long term | Some concerns |
| Klesges 2010 | 5-11 | High | Mixed | Diet and physical activity vs control | Intervention(s): 153 Control: 150 | BMI | Medium term Long term | Some concerns |
| Kobel 2017 | 5-11 | High | Mixed | Diet and physical activity vs control | Intervention(s): 307 Control: 200 | BMI Percentile | Medium term | High risk |
| Kocken 2016 | 5-11 | High | Mixed | Diet and physical activity vs control | Intervention(s): 615 Control: 497 | zBMI | Short term Long term | High risk |
| Kovalskys 2016 | 5-11 | Non-High | Mixed | Physical activity vs control | Intervention(s): 424 Control: 336 | zBMI | Long term | High risk |
| Kriemler 2010 | 5-11 | High | Mixed | Physical activity vs control | Intervention(s): 297 Control: 205 | BMI | Medium term Long term | Low risk (medium term) High risk (long term) |
| Kubik 2021 | 5-11 | High | Mixed | Diet and physical activity vs control | Intervention(s): 66 Control: 66 | zBMI BMI | Medium term Long term | Some concerns |
| Kuhlemeier 2022 | 12-18 | High | Low | Diet and physical activity vs control | Intervention(s): 318 Control: 290 | zBMI | Long term | High risk |
| Kuroko 2020 | 12-18 | High | Mixed | Diet vs control | Intervention(s): 88 Control: 27 | zBMI | Medium term | Some concerns |
| Lappe 2017** | 12-18 | High | Mixed | Diet vs control | Intervention(s): 136 Control: 138 | Percentile | Medium term | Some concerns |
| Lau 2016 | 5-11 | Non-High | Mixed | Physical activity vs control | Intervention(s): 40 Control: 40 | BMI | Short term | Some concerns |
| Lazaar 2007 | 5-11 | High | Mixed | Physical activity vs control | Intervention(s): 138 Control: 187 | zBMI BMI | Short term | Some concerns |
| Leme 2018 | 12-18 | Non-High | Low | Diet and physical activity vs control | Intervention(s): 142 Control: 111 | zBMI BMI | Short term Medium term | Some concerns |
| Lent 2014 | 5-11 | High | Low | Diet vs control | Intervention(s): 435 Control: 332 | zBMI BMI Percentile | Medium term Long term | Some concerns (medium term) High risk (long term) |
| Levy 2012 | 5-11 | Non-High | Mixed | Diet and physical activity vs control | Intervention(s): 510 Control: 509 | Proportion | Short term | Some concerns |
| Li 2010 | 5-11 | Non-High | Mixed | Physical activity vs control | Intervention(s): 2329 Control: 2371 | zBMI BMI | Medium term Long term | Some concerns |
| Li 2019 | 5-11 | Non-High | Mixed | Diet and physical activity vs control | Intervention(s): 832 Control: 809 | zBMI | Medium term | Low risk |
| Lichtenstein 2011 | 5-11 | High | Mixed | Diet and physical activity vs control | Intervention(s): 249 Control: 196 | zBMI | Medium term Long term | High risk |
| Liu 2019 | 5-11 | Non-High | Mixed | Diet and physical activity vs control | Intervention(s): 930 Control: 959 | zBMI BMI | Short term Medium term | Some concerns |
| Liu 2022 | 5-11 | Non-High | Mixed | Diet and physical activity vs control | Intervention(s): 703 Control: 670 | zBMI BMI | Short term Medium term | Low risk |
| Llargues 2012 | 5-11 | High | Mixed | Diet and physical activity vs control | Intervention(s): 225 Control: 201 | BMI | Long term | Some concerns |
| Lloyd 2018 | 5-11 | High | Mixed | Diet and physical activity vs control | Intervention(s): 676 Control: 648 | zBMI BMI | Long term | Low risk |
| Lubans 2021 | 12-18 | High | Mixed | Physical activity vs control | Intervention(s): 333 Control: 328 | zBMI | Short term Medium term | Some concerns (short term) High risk (medium term) |
| Luszczynska 2016b | 12-18 | High | Mixed | Diet (two groups) vs control | Intervention(s): Planning: 153; Self-efficacy: 172 Control: 181 | BMI | Medium term | High risk |
| Magnusson 2012 | 5-11 | High | Mixed | Diet and physical activity vs control | Intervention(s): 103 Control: 82 | BMI | Long term | High risk |
| Marcus 2009 | 5-11 | High | Mixed | Diet and physical activity vs control | Intervention(s): 1538 Control: 1300 | zBMI | Long term | High risk |
| Martinez-Vizcaino 2014 | 5-11 | High | Mixed | Physical activity vs control | Intervention(s): 229 Control: 240 | BMI | Medium term | Some concerns |
| Martinez-Vizcaino 2020 | 5-11 | High | Mixed | Physical activity vs control | Intervention(s): 619 Control: 815 | zBMI BMI | Short term | High risk |
| Martinez-Vizcaino 2022 | 5-11 | High | Mixed | Physical activity vs control | Intervention(s): 248 Control: 239 | zBMI BMI | Medium term | High risk |
| Melnyk 2013 | 12-18 | High | Mixed | Physical activity vs control | Intervention(s): 358 Control: 421 | BMI | Short term Medium term | High risk |
| Meng 2013 (Beijing) | 5-11 | Non-High | Mixed | Diet vs Physical activity vs control | Intervention(s): Nutrition education: 615; Happy 10 intervention: 590 Control: 460 | zBMI BMI | Medium term | High risk |
| Mihas 2010 | 12-18 | High | Mixed | Diet vs control | Intervention(s): 98 Control: 93 | BMI | Medium term | Some concerns |
| Morgan 2011 | 5-11 | High | Mixed | Diet and physical activity vs control | Intervention(s): 39 Control: 32 | zBMI | Short term | High risk |
| Morgan 2014 | 5-11 | High | Mixed | Diet and physical activity vs control | Intervention(s): 72 Control: 60 | zBMI BMI | Short term | Some concerns |
| Morgan 2019 | 5-11 | High | Mixed | Physical activity vs control | Intervention(s): 74 Control: 79 | zBMI | Medium term | Some concerns |
| Muller 2016 | 5-11 | High | Mixed | Physical activity vs control | Intervention(s): 109 Control: 73 | zBMI | Medium term | Some concerns |
| Muller 2019 | 5-11 | Non-High | Mixed | Physical activity vs control | Intervention(s): 300 Control: 446 | zBMI | Medium term | High risk |
| Muzaffar 2019 | 5-11 | High | Mixed | Diet and physical activity vs Diet and physical activity | Intervention(s): PAWS Peer led: 49; PAWS Adult led: 52 | Percentile | Short term Medium term | High risk |
| NCT00224887 2005 | 5-11 | High | Low | Diet vs control | Intervention(s): 154 Control: 153 | BMI | Medium term | Some concerns |
| NCT02067728 2014 | 5-11; 12-18 | High | Mixed | Diet and physical activity vs control | Intervention(s): 45 Control: 44 | zBMI | Short term | High risk |
| Nemet 2011a | 5-11 | High | Low | Diet and physical activity vs control | Intervention(s): 68 Control: 62 | BMI Percentile | Medium term | Some concerns |
| Nemet 2011b | 5-11 | High | Low | Diet and physical activity vs control | Intervention(s): 118 Control: 85 | BMI Percentile | Medium term Long term | Some concerns (medium term) High risk (long term) |
| Neumark-Sztainer 2003 | 12-18 | High | Mixed | Diet and physical activity vs control | Intervention(s): 89 Control: 112 | BMI | Short term | High risk |
| Neumark-Sztainer 2010 | 12-18 | High | Mixed | Diet and physical activity vs control | Intervention(s): 182 Control: 174 | BMI | Short term Medium term | Some concerns |
| Newton 2014 | 5-11 | High | Mixed | Physical activity vs control | Intervention(s): 13 Control: 14 | zBMI BMI Percentile | Short term | Some concerns |
| Nicholl 2021 | 5-11 | High | Mixed | Diet vs control | Intervention(s): 24 Control: 21 | zBMI BMI Percentile | Short term | Some concerns |
| Nollen 2014 | 5-11 | High | Mixed | Diet and physical activity vs control | Intervention(s): 26 Control: 25 | BMI | Short term | High risk |
| Nyberg 2015 | 5-11 | High | Low | Diet and physical activity vs control | Intervention(s): 129 Control: 112 | Proportion | Short term | Some concerns |
| Nyberg 2016 | 5-11 | High | Low | Diet and physical activity vs control | Intervention(s): 185 Control: 193 | zBMI | Short term Medium term | Some concerns |
| O'Connor 2020 | 5-11 | High | Mixed | Diet and physical activity vs control | Intervention(s): 31 Control: 33 | zBMI | Short term | Some concerns |
| Ooi 2021 | 12-18 | High | Low | Diet vs control | Intervention(s): 162 Control: 134 | Proportion | Short term | High risk |
| Paineau 2008 | 5-11 | High | Mixed | Diet (two groups) vs control | Intervention(s): Group A: 280; Group B: 275 Control: 394 | zBMI BMI | Short term | Some concerns |
| Papadaki 2010 | 12-18 | High | Mixed | Diet (four groups) vs control | Intervention(s): LP/LGI: 101; LP)/HGI: 85; HP)/LGI: 91; HP)/HGI: 95 Control: 88 | zBMI BMI | Short term | High risk |
| Pate 2005 | 12-18 | High | Mixed | Physical activity vs control | Intervention(s): 827 Control: 712 | Proportion | Medium term | Some concerns |
| Patrick 2006 | 12-18 | High | Mixed | Diet and physical activity vs control | Intervention(s): 1611 Control: 411 | zBMI BMI | Short term | High risk |
| Peralta 2009 | 12-18 | High | Mixed | Diet and physical activity vs control | Intervention(s): 16 Control: 17 | BMI | Short term | Some concerns |
| Pfeiffer 2019 | 12-18 | High | Low | Physical activity vs control | Intervention(s): 753 Control: 766 | zBMI | Short term | High risk |
| Prins 2012 | 12-18 | High | Mixed | Physical activity (two groups) vs control | Intervention(s): YouRAction:118; YouRAction+e: 136 Control: 132 | Proportion | Short term | Some concerns |
| Puder 2011 | 5-11 | High | Mixed | Diet and physical activity vs control | Intervention(s): 342 Control: 310 | BMI | Medium term | Low risk |
| Ramirez-Rivera 2021 | 5-11 | Non-High | Mixed | Diet and physical activity vs control | Intervention(s): 21 Control: 20 | zBMI | Short term | Some concerns |
| Reesor 2019 | 12-18 | High | Low | Diet and physical activity vs control | Intervention(s): 101 Control: 90 | zBMI | Short term Medium term | High risk |
| Rerksuppaphol 2017 | 5-11 | Non-High | Mixed | Diet and physical activity vs control | Intervention(s): 111 Control: 106 | zBMI BMI | Short term | Some concerns |
| Rhodes 2019 | 5-11 | High | Mixed | Physical activity vs control | Intervention(s): 52 Control: 50 | BMI | Short term | Some concerns |
| Robinson 2003 | 5-11 | High | Mixed | Physical activity vs Diet and physical activity | Intervention(s): Physical activity: 28; Diet and physical activity: 33 | BMI | Short term | Some concerns |
| Robinson 2010 | 5-11 | High | Mixed | Physical activity vs Diet and physical activity | Intervention(s): Physical activity: 134; Diet and physical activity: 127 | zBMI BMI | Long term | High risk |
| Rodearmel 2006 | 12-18 | High | Mixed | Diet and physical activity vs control | Intervention(s): 52 Control: 19 | Percentile | Short term | High risk |
| Rosario 2012 | 5-11 | High | Mixed | Diet and physical activity vs control | Intervention(s): 231 Control: 233 | zBMI BMI | Short term | Some concerns |
| Rosenkranz 2010 | 5-11 | High | Mixed | Diet and physical activity vs control | Intervention(s): 33 Control: 39 | zBMI BMI Percentile | Short term | Some concerns |
| Rush 2012 | 5-11 | High | Mixed | Diet and physical activity vs control | Intervention(s): 692 Control: 660 | zBMI | Long term | High risk |
| Sacchetti 2013 | 5-11 | High | Mixed | Physical activity vs control | Intervention(s): 212 Control: 216 | BMI | Long term | Some concerns |
| Safdie 2013 | 5-11 | Non-High | Low | Diet and physical activity (two groups) vs control | Intervention(s): Basic program: 252; Plus program: 224 Control: 354 | BMI | Short term Medium term Long term | Some concerns |
| Sahota 2001 | 5-11 | High | Mixed | Diet and physical activity vs control | Intervention(s): 314 Control: 322 | zBMI | Medium term | Some concerns |
| Sahota 2019 | 5-11 | High | Mixed | Diet and physical activity vs control | Intervention(s): 188 Control: 170 | zBMI | Long term | Some concerns |
| Salmon 2022 | 5-11 | High | Mixed | Physical activity (three groups) vs control | Intervention(s): Physical activity: 153; Sedentary behaviour: 120; Physical activity + Sedentary behaviour: 150 Control: 141 | zBMI | Long term | Some concerns |
| Santos 2014 | 5-11 | High | Mixed | Diet and physical activity vs control | Intervention(s): 340 Control: 307 | zBMI | Medium term | Some concerns |
| Schreier 2013 | 12-18 | High | Low | Diet and physical activity vs control | Intervention(s): 54 Control: 54 | BMI | Short term | Some concerns |
| Seguin-Fawler 2021 | 5-11 | High | Mixed | Diet vs control | Intervention(s): 148 Control: 157 | Percentile | Short term | Some concerns |
| Sekhavat 2014 | 5-11 | High | Mixed | Diet and physical activity vs control | Intervention(s): 87 Control: 81 | zBMI BMI | Medium term | Some concerns |
| Sgambato 2019 | 5-11 | Non-High | Low | Diet and physical activity vs control | Intervention(s): 1290 Control: 1157 | BMI | Short term | Some concerns |
| Sherwood 2019 | 5-11 | High | Mixed | Diet and physical activity vs control | Intervention(s): 212 Control: 209 | zBMI Percentile | Medium term Long term | Some concerns |
| Shin 2015 | 12-18 | High | Low | Diet vs control | Intervention(s): 89 Control: 63 | Percentile | Medium term | Some concerns |
| Shomaker 2019 | 12-18 | High | Mixed | Diet vs control | Intervention(s): 29 Control: 25 | zBMI BMI Percentile | Short term Long term | Some concerns |
| Sichieri 2008 | 5-11 | Non-High | Low | Diet vs control | Intervention(s): 526 Control: 608 | BMI | Short term | Some concerns |
| Siegrist 2013 | 5-11 | High | Mixed | Diet and physical activity vs control | Intervention(s): 422 Control: 297 | zBMI BMI | Medium term | Some concerns |
| Siegrist 2018 | 5-11 | High | Mixed | Diet and physical activity vs control | Intervention(s): 243 Control: 191 | BMI | Long term | Some concerns |
| Simon 2008 | 5-11 | High | Mixed | Physical activity vs control | Intervention(s): 374 Control: 367 | zBMI BMI | Medium term (BMI only) Long term | Some concerns |
| Simons 2015 | 12-18 | High | Mixed | Physical activity vs control | Intervention(s): 134 Control: 126 | zBMI | Short term Medium term | Some concerns |
| Singh 2009 | 12-18 | High | Mixed | Diet and physical activity vs control | Intervention(s): 601 Control: 452 | BMI | Short term Medium term Long term | Some concerns |
| Smith 2014 | 12-18 | High | Low | Physical activity vs control | Intervention(s): 181 Control: 180 | BMI | Short term | Some concerns |
| Spiegel 2006 | 5-11 | High | Mixed | Diet and physical activity vs control | Intervention(s): 534 Control: 479 | Proportion | Short term | High risk |
| Stettler 2015 | 5-11 | High | Mixed | Diet vs Diet and physical activity vs control | Intervention(s): Smart Steps - beverage-only: 76; Smart Steps - multiple behaviour: 63  Control: 33 | zBMI BMI | Medium term | Some concerns |
| Stolley 1997 | 5-11 | High | Low | Diet and physical activity vs control | Intervention(s): 18 Control: 22 | BMI | Short term Medium term | High risk |
| Story 2003 | 5-11 | High | Low | Diet and physical activity vs control | Intervention(s): 26 Control: 28 | BMI | Short term | Some concerns |
| Story 2012 | 5-11 | High | Low | Diet and physical activity vs control | Intervention(s): 267 Control: 187 | zBMI BMI | Long term | High risk |
| Takacs 2020 | 12-18 | High | Mixed | Diet vs control | Intervention(s): 114 Control: 105 | BMI | Medium term | Some concerns |
| Tanskey 2017 | 5-11 | High | Low | Physical activity (two groups) vs control | Intervention(s): 100 Miles club: 261; Just Move: 249 Control: 259 | zBMI BMI | Medium term | High risk |
| Telford 2012 | 5-11 | High | Mixed | Physical activity vs control | Intervention(s): 312 Control: 308 | BMI | Long term | High risk |
| Tessier 2008 | 5-11 | High | Mixed | Physical activity vs Physical activity | Intervention(s): REGU'LAPS: 474; Active control: 465 | BMI | Short term | High risk |
| Thivel 2011 | 5-11 | High | Mixed | Physical activity vs control | Intervention(s): 168 Control: 187 | BMI | Short term | High risk |
| Topham 2021 | 5-11 | High | Mixed | Diet and physical activity (four groups) vs control | Intervention(s): Family Lifestyle: 117; Family Lifestyle + Family Dynamics: 87; Family Dynamic + Peer Group: 124; Family Lifestyle + Family Dynamic + Peer Group: 129  Control: 81 | zBMI | Long term | High risk |
| van de Berg 2020 | 5-11 | High | Low | Diet vs Physical activity vs Diet and physical activity vs control | Intervention(s): Walk Across Texas (WAT!): 336; Learn!Grow! Eat!Go! (LGEG!): 347; Walk Across Texas (WAT!) + Learn!Grow! Eat!Go! (LGEG!): 358 Control: 285 | Percentile | Medium term | High risk |
| Velez 2010 | 12-18 | High | Mixed | Physical activity vs control | Intervention(s): 13 Control: 15 | BMI | Short term | Some concerns |
| Viggiano 2015 | 12-18 | High | Mixed | Diet vs control | Intervention(s): 1663 Control: 1447 | zBMI | Short term Long term | Some concerns |
| Viggiano 2018 | 5-11 | High | Mixed | Diet vs control | Intervention(s): 837 Control: 476 | zBMI | Short term Long term | High risk |
| Vizcaino 2008 | 5-11 | High | Mixed | Physical activity vs control | Intervention(s): 513 Control: 606 | BMI | Medium term | Some concerns |
| Wang 2012 | 5-11 | Non-High | Mixed | Diet and physical activity vs control | Intervention(s): 476 Control: 527 | Proportion | Medium term | High risk |
| Wang 2018 | 5-11 | Non-High | Mixed | Physical activity vs control | Intervention(s): 5275 Control: 4583 | zBMI BMI | Medium term | Some concerns |
| Weeks 2012 | 12-18 | High | Mixed | Physical activity vs control | Intervention(s): 52 Control: 47 | BMI | Short term | Some concerns |
| Wendel 2016 | 5-11 | High | Mixed | Physical activity vs control | Intervention(s): 121 Control: 72 | BMI Percentile | Long term | High risk |
| White 2019 | 5-11 | High | Low | Diet and physical activity vs control | Intervention(s): 151 Control: 77 | zBMI | Short term Medium term Long term | High risk |
| Whittemore 2013 | 12-18 | High | Mixed | Diet and physical activity vs Diet and physical activity | Intervention(s): HEALTH[e]TEEN+CST: 204; HEALTH[e]TEEN: 174 | BMI | Short term | Some concerns |
| Wieland 2018 | 12-18 | High | Low | Diet and physical activity vs control | Intervention(s): 40 Control: 41 | BMI | Short term Medium term | Some concerns |
| Wilksch 2015 | 12-18 | High | Mixed | Diet and physical activity vs control | Intervention(s): 347 Control: 473 | BMI | Short term Medium term | Some concerns |
| Williamson 2012 | 5-11 | High | Low | Diet and physical activity vs control | Intervention(s): 713 Control: 587 | zBMI | Long term | Some concerns |
| Xu 2015 | 5-11 | Non-High | Mixed | Diet and physical activity vs control | Intervention(s): 612 Control: 513 | zBMI BMI |  | Some concerns |
| Xu 2017 (5 other cities) | 5-11 | Non-High | Mixed | Diet and physical activity vs control | Intervention(s): 2656 Control: 2627 | zBMI BMI | Medium term | Some concerns |
| Yin 2012 | 5-11 | High | Mixed | Physical activity vs control | Intervention(s): 292 Control: 284 | zBMI | Medium term Long term | Some concerns (medium term) High risk (long term) |

# Selecting indicator variables

The correlation matrix between the intervention indicators is shown in **Figure S*1***. We observed two absolute correlations $\geq0.5$ between (i) the total duration of the intervention and the duration of the peak engagement period (correlation of 0.66), and (ii) school setting and community setting (correlation of -0.72). Based on (i) we chose to remove peak duration from our set of indicators. Correlation (ii) reflects the fact that interventions tended to be based in either a school or a community environment, while home elements were often in supplement to one of these settings. Since the effect of all settings were deemed of interest by our stakeholders, we did not remove either of these variables.

**Figure S*2*** shows the proportion of interventions that were coded as 1 (rather than 0) for each intervention indicator variable. We observed six indicators that yielded identical responses more than 80% of the time: delivered in the home (81.6% No), targets activity (81.2% Yes), applied continuously (96.5% Yes), delivered to the child as part of a group (82.7% Yes), involved a single phase (81.2% Yes), commercial interests involved (89.4% No). Based on these results, we chose to remove the continuous, single phase and commercial interests indicators due to a lack of information. Next, we re-defined the home indicator to also include `home activity’. This meant that home was coded as 1 if the intervention was delivered in the home OR included some home based activity for the child. We also re-coded the group and individual indicators as one variable coded as 1 if the intervention was delivered to the child individually (either exclusively or in combination with group delivery) and 0 if there was no individual element. Finally, we chose to combine the flexibility and choice indicators which, while not quite reaching our 80% threshold, were both mostly coded as 0 (No) and represent a similar concept (i.e. the adaptability of the intervention to the preferences of the recipient or deliverer of the intervention). Since evaluating the effect of activity based interventions is of particular interest, we did not remove this indicator.


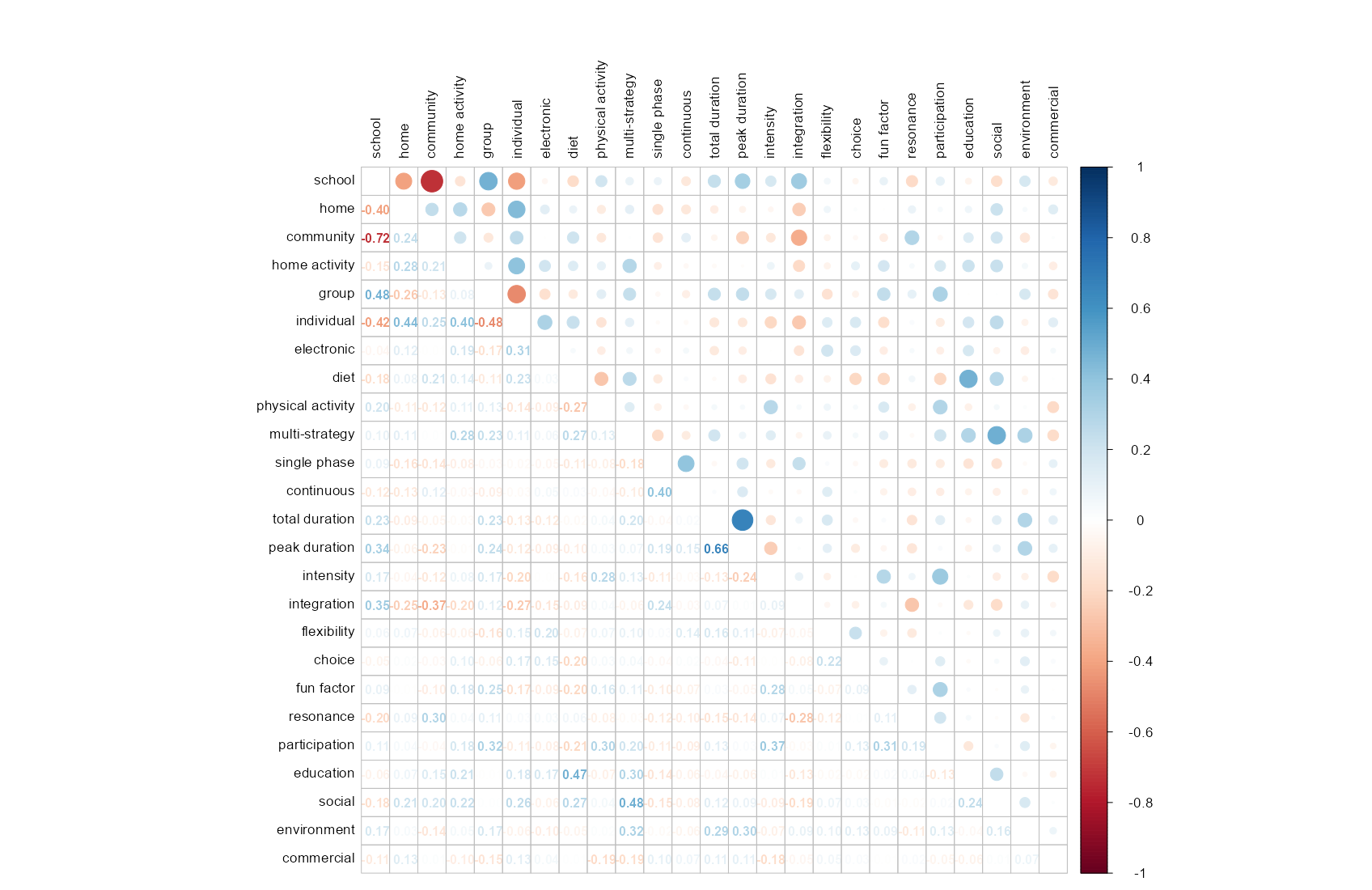


**Figure S1:** Correlation matrix describing the collinearity between pairs of intervention indicators. The colour and size of circles in the upper half of the matrix indicates the correlation between those variables. Negative correlations are indicated in red and positive correlations in blue (as shown in the key on the right hand side). The value of the correlation is shown in the equivalent box in the lower half of the matrix. The lowest correlations are very faint and sometimes cannot be seen.


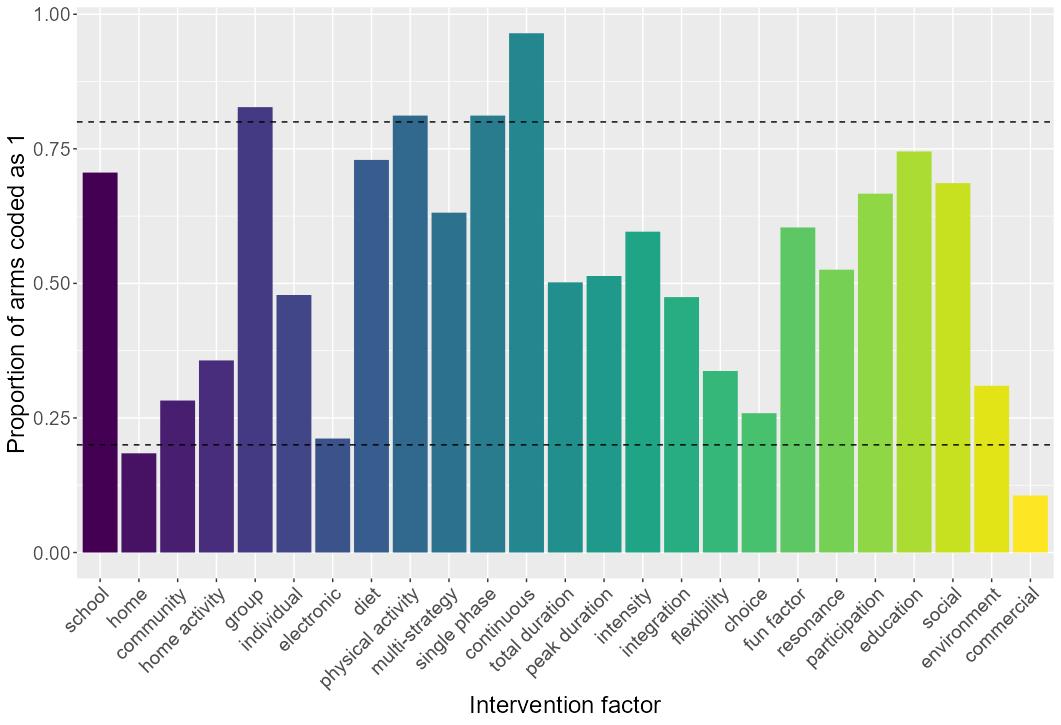


**Figure S2:** Bar chart showing the proportion of identical responses for each intervention indicator. Each bar represents the proportion of intervention arms coded as 1 in the data set (see Section A for a description of each indicator and its coding). The horizontal dotted lines are at 0.2 and 0.8 representing our preliminary cut off of 80% identical responses (for 0 and 1 respectively).

# Selecting interactions via SSVS

The results of the SSVS models at each step are shown in Sections I.1 (**Figure S3**, **Figure S4**, and **Figure S5**) and I.2 (**Figure S6**, **Figure S7**, and **Figure S8**) for the primary (random effects) and secondary (fixed effects) analyses respectively. **Table S*2*** compares the fit of the different models using the deviance information criterion (DIC) [9]. For both the RE and FE procedures, the final model with selected interactions has the lowest DIC indicating the best fit. The RE models have lower DIC (better fit) than the FE models.

**Table S2.** Deviance information criterion (DIC) for the models fitted at each step of the SSVS procedure (for both random effects and fixed effects).

| Model | Interactions | DIC | |
| --- | --- | --- | --- |
|  |  | **RE** | **FE** |
| SSVS step 1 | No interactions | -560.9 | -184.7 |
| SSVS step 2 | Interactions with age and all other indicators | -565.8 | -217.9 |
| SSVS step 3 | Interactions with behaviour targeted and all other indicators | -560.8 | -265.5 |
| Primary | Selected interactions | -579.3 | -305.3 |

## Primary analysis (random effects)

### Step 1: No interactions


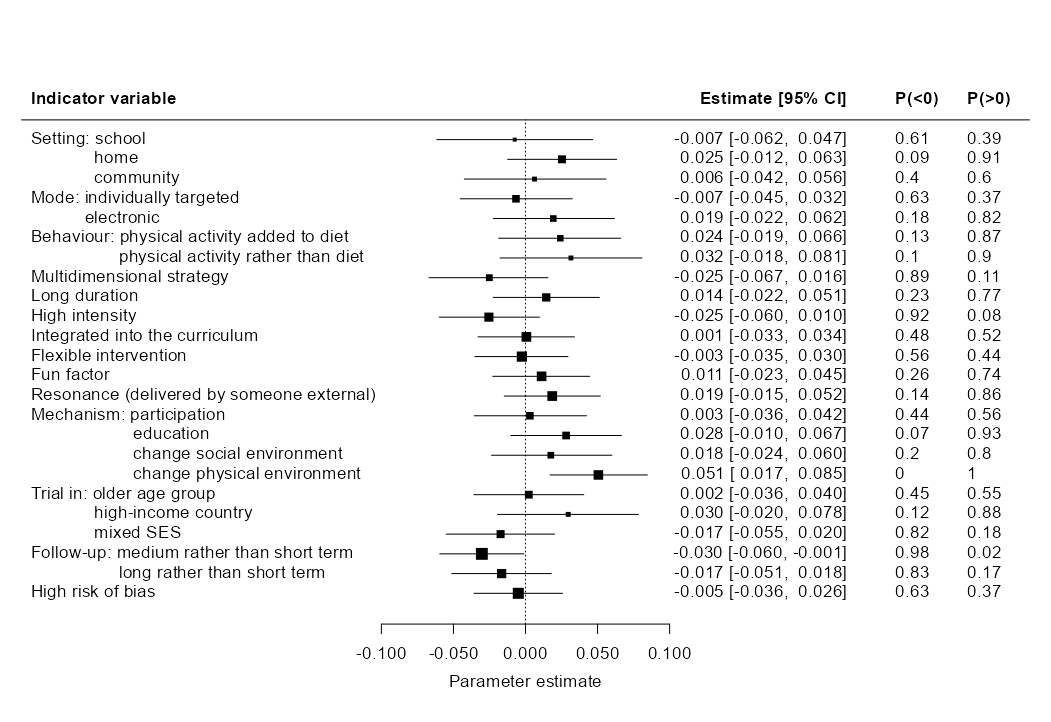


**Figure S3:** Parameter estimates from step 1 of the SSVS procedure (model with no interactions) for the primary (random effects) model. We list the probability that each coefficient is less than or greater than zero, $P(<0)$ and $P(>0)$. The estimates of the intercept and heterogeneity parameter are $\alpha\left[ 95\% CI \right]=-0.039[-0.054,-0.023]$ and $\tau\left[ 95\% CI \right]=0.085 [0.072, 0.099]$ respectively.

### Step 2: Interactions with age


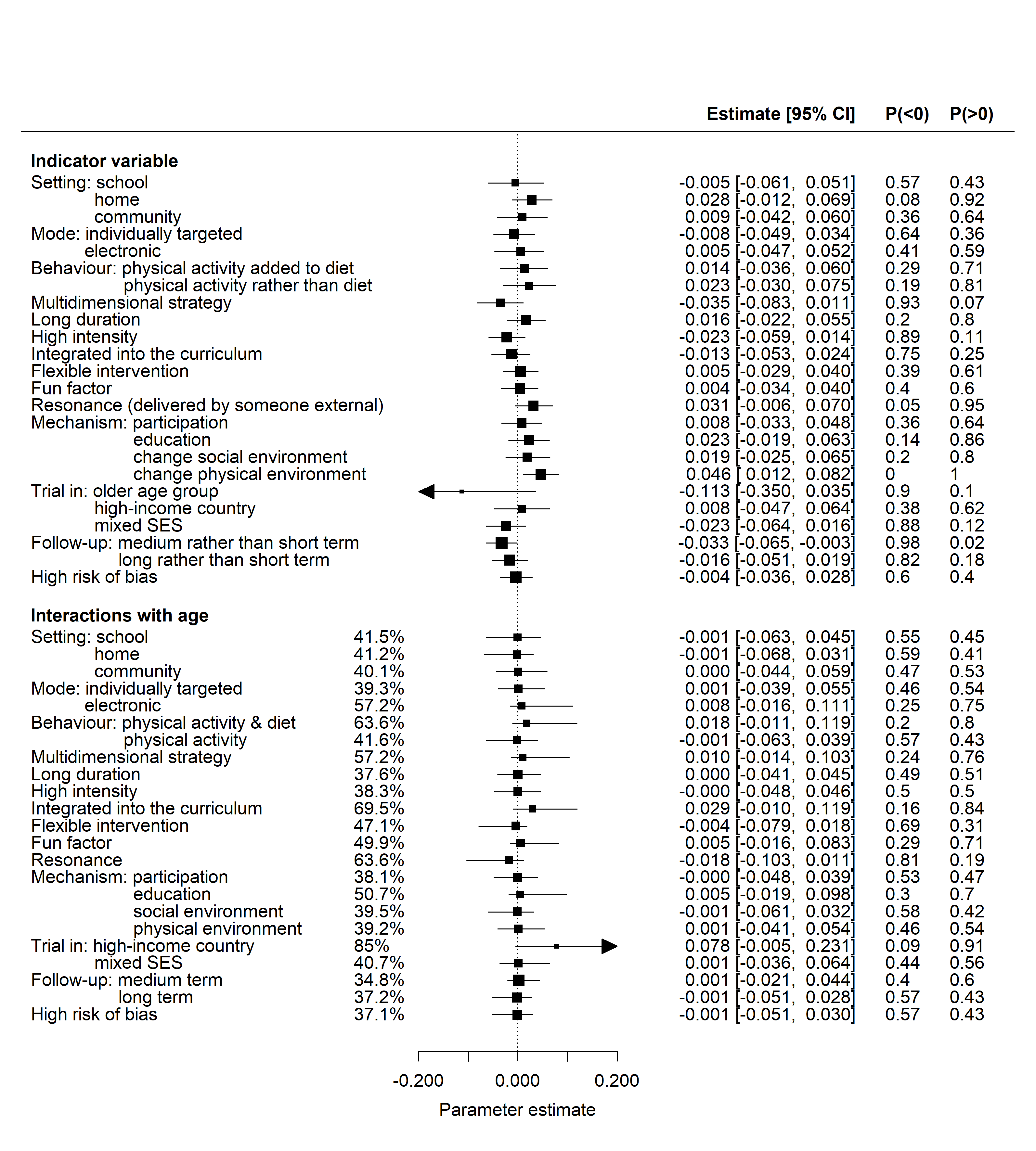


**Figure S4:** Parameter estimates from step 2 of the SSVS procedure (interactions between age and all other indicators) for the primary (random effects) model. We list the probability that each coefficient is less than or greater than zero, $P(<0)$ and $P(>0)$. For each interaction term we list the percentage of times it was selected by the SSVS model. The estimates of the intercept and heterogeneity parameter are $\alpha\left[ 95\% CI \right]=-0.038 [-0.053,-0.023]$ and $\tau\left[ 95\% CI \right]=0.082 [0.070, 0.097]$ respectively.

### Step 3: Interactions with change in behaviour targeted


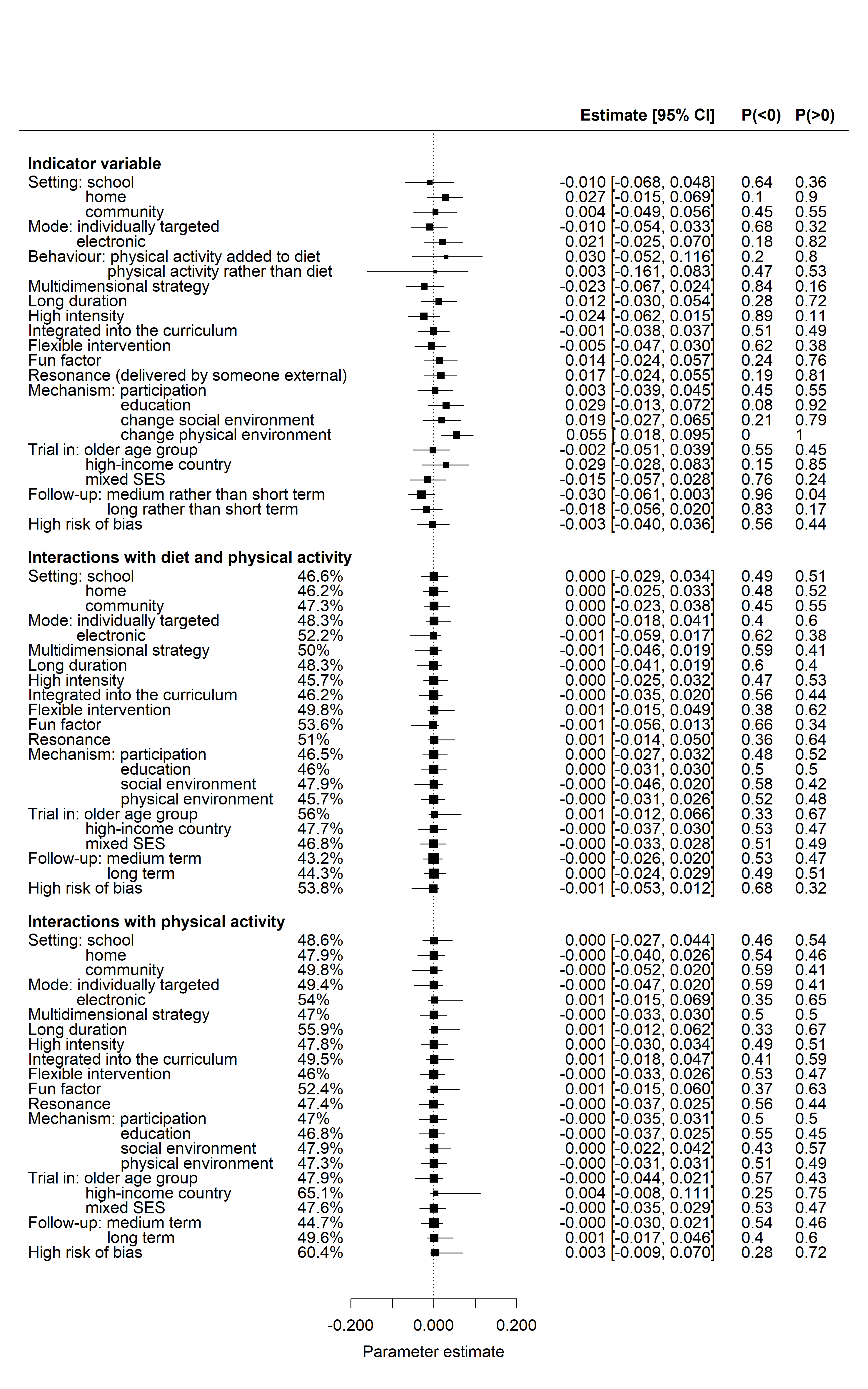


**Figure S5:** Parameter estimates from step 3 of the SSVS procedure (interactions between behaviour targeted (diet and/or activity) and all other indicators) for the primary (random effects) model. We list the probability that each coefficient is less than or greater than zero, $P(<0)$ and $P(>0)$. For each interaction term we list the percentage of times it was selected by the SSVS model. The estimates of the intercept and heterogeneity parameter are $\alpha\left[ 95\% CI \right]=-0.039 [-0.054,-0.023]$ and $\tau\left[ 95\% CI \right]=0.084 [0.071, 0.098]$ respectively.

## Secondary analysis (fixed effects)

### Step 1: No interactions


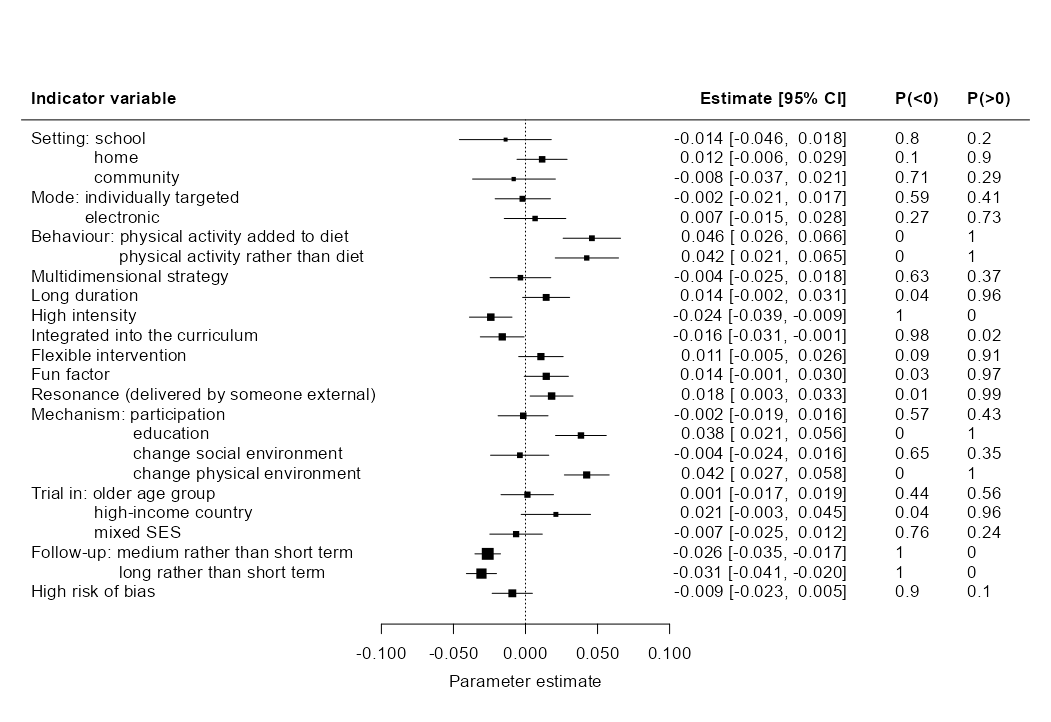


**Figure S6:** Parameter estimates from step 1 of the SSVS procedure (model with no interactions) for the secondary (fixed effects) model. We list the probability that each coefficient is less than or greater than zero, $P(<0)$ and $P(>0)$. The estimate of the intercept is $\alpha\left[ 95\% CI \right]=-0.033[-0.041,-0.025]$.

### Step 2: Interactions with age


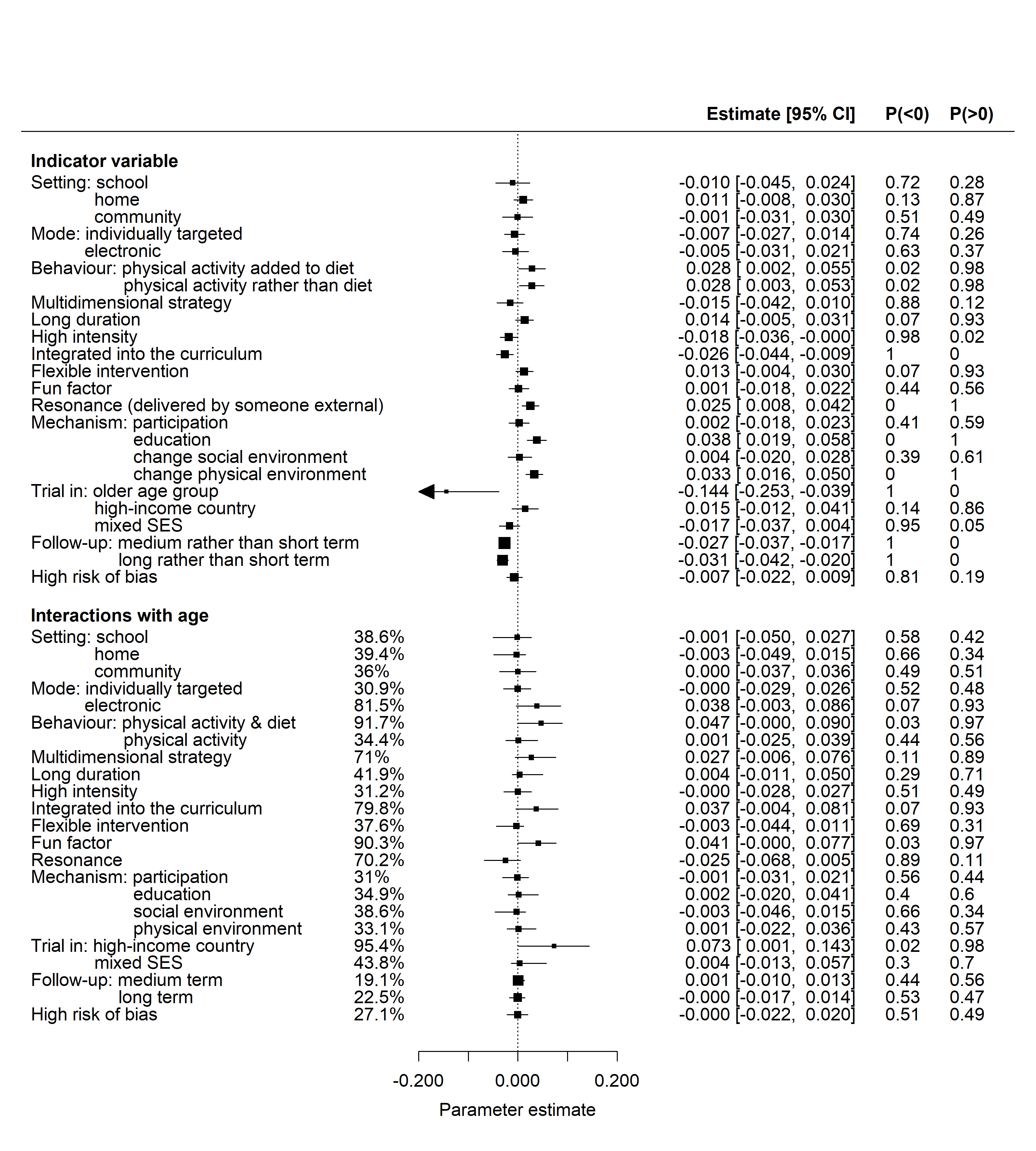


**Figure S7:** Parameter estimates from step 2 of the SSVS procedure (interactions between age and all other indicators) for the secondary (fixed effects) model. For each interaction term we list the percentage of times it was selected by the SSVS model. We list the probability that each coefficient is less than or greater than zero, $P(<0)$ and $P(>0)$. The estimate of the intercept is $\alpha\left[ 95\% CI \right]=-0.034 [-0.042,-0.026]$.

### Step 3: interactions with change in behaviour targeted


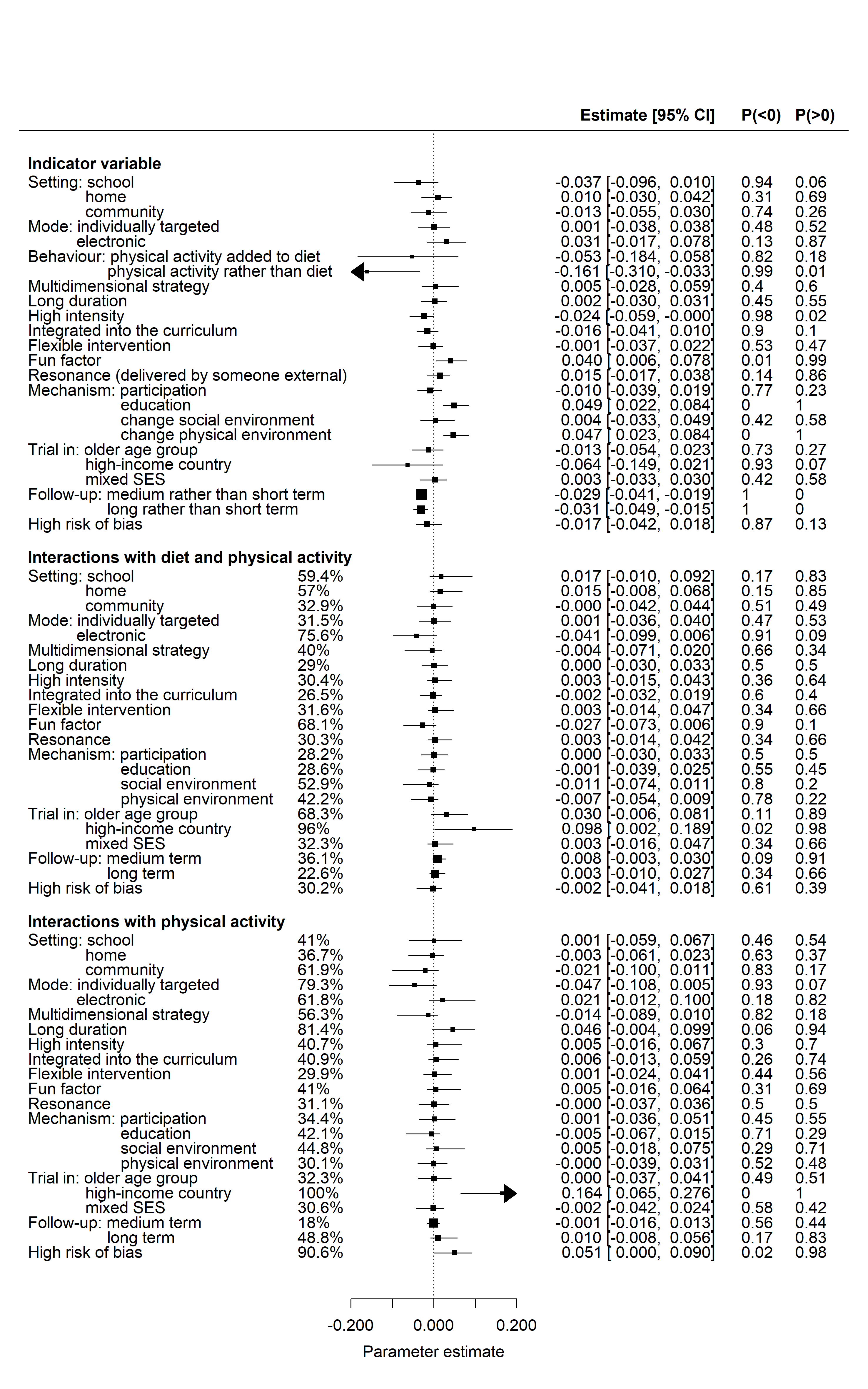


**Figure S8:** Parameter estimates from step 3 of the SSVS procedure (interactions between behaviour targeted (diet and/or activity) and all other indicators) for the secondary (fixed effects) model. We list the probability that each coefficient is less than or greater than zero, $P(<0)$ and $P(>0)$. For each interaction term we list the percentage of times it was selected by the SSVS model. The estimate of the intercept is $\alpha\left[ 95\% CI \right]=-0.032 [-0.041,-0.024]$.

# Comparing random and fixed effects models

Here we summarize the differences between the RE and FE analyses. We identify effects with strong evidence as those with probabilities in either direction ($P\left( <0 \right)$ or $P\left( >0 \right)$) that are greater than or equal to 0.95. For probabilities that exceed or equal 0.9, we label these effects as having reasonable evidence. Any effect which has strong evidence in the same direction in both analyses, we consider ‘robust’. Any effect which has strong evidence in one analysis and reasonable evidence in the other, we consider ‘almost robust’. Based on this procedure, we find robust indicator effects (**Table S3**) for physical activity alone versus diet alone (beneficial), environmental components (less beneficial), older age group (beneficial), high-income countries (beneficial) and medium verses short term (beneficial). The beneficial effect of integration is almost robust. We find robust antagonistic interactions (**Table S4**) between (i) the older age group and diet and physical activity interventions, (ii) the older age group and high-income countries, (iii) activity only and long interventions, and (iv) activity only interventions and high-income countries. Finally, we find an almost robust antagonistic interaction between the older age group and integrated interventions.

**Table S3:** A comparison of the direction and strength of evidence for indicator coefficients in the primary (random effects) and secondary (fixed effects) analyses. A coefficient direction <0 indicates a beneficial effect of an indicator coded as 1 and a direction >0 indicates a less beneficial effect. We categorize evidence as ‘strong’ if the probability that the coefficient takes a value one side of zero is greater than or equal to 0.95 and ‘reasonable’ if the probability is greater than or equal to 0.9. We label a coefficient as ‘robust’ if both analyses provide strong evidence in the same direction and ‘almost robust’ if one analyses is strong and the other is reasonable (in the same direction).

|  | Primary analysis (RE) | | Secondary analysis (FE) | | Robustness |
| --- | --- | --- | --- | --- | --- |
| Indicator | **Direction** | **Strength** | **Direction** | **Strength** |  |
| School | - | - | <0 | Strong |  |
| Home | >0 | Strong | - | - |  |
| Community | - | - | - | - |  |
| Individual | - | - | - | - |  |
| Electronic | - | - | - | - |  |
| Diet & activity | - | - | <0 | Strong |  |
| Activity | <0 | Strong | <0 | Strong | Robust |
| Multi-strategy | <0 | Reasonable | - | - |  |
| Duration | - | - | - | - |  |
| Intensity | - | - | <0 | Strong |  |
| Integration | <0 | Reasonable | <0 | Strong | Almost robust |
| Flexibility/choice | - | - | - | - |  |
| Fun factor | - | - | >0 | Strong |  |
| Resonance | - | - | >0 | Strong |  |
| Participation | - | - | - | - |  |
| Education | - | - | >0 | Strong |  |
| Social | - | - | - | - |  |
| Environment | >0 | Strong | >0 | Strong | Robust |
| Age | <0 | Strong | <0 | Strong | Robust |
| Income country | <0 | Strong | <0 | Strong | Robust |
| SES | - | - | - | - |  |
| Medium term | <0 | Strong | <0 | Strong | Robust |
| Long term | - | - | <0 | Strong |  |
| Risk of bias | - | - | <0 | Strong |  |

**Table S4:** A comparison of the direction and strength of evidence for interaction coefficients in the primary (random effects) and secondary (fixed effects) analyses. A coefficient direction <0 indicates a synergistic interaction between the two *indicator*s and a direction >0 indicates an antagonistic interaction. We categorize evidence as ‘strong’ if the probability that the coefficient takes a value one side of zero is greater than or equal to 0.95 and ‘reasonable’ if the probability is greater than or equal to 0.9. We label a coefficient as ‘robust’ if both analyses provide strong evidence in the same direction and ‘almost robust’ if one analyses is strong and the other is reasonable (in the same direction).

|  | Primary analysis (RE) | | Secondary analysis (FE) | | | Robustness |
| --- | --- | --- | --- | --- | --- | --- |
| Interaction | **Direction** | **Strength** | | **Direction** | **Strength** |  |
| Interactions with age | | | | | | |
| Electronic | - | - | | - | - |  |
| Diet & activity | >0 | Strong | | >0 | Strong | Robust |
| Multi-strategy | - | - | | - | - |  |
| Integration | >0 | Reasonable | | >0 | Strong | Almost robust |
| Fun factor | *Not selected* |  | | - | - |  |
| Resonance | - | - | | <0 | Reasonable |  |
| Education | - | - | | *Not selected* |  |  |
| Income country | >0 | Strong | | >0 | Strong | Robust |
| Interactions with diet & activity | | | | | | |
| School | *Not selected* |  | | >0 | Strong |  |
| Home | *Not selected* |  | | >0 | Reasonable |  |
| Electronic | - | - | | <0 | Strong |  |
| Multi-strategy | - | - | | *Not selected* |  |  |
| Fun factor | - | - | | <0 | Strong |  |
| Resonance |  |  | | *Not selected* |  |  |
| Social | *Not selected* |  | | - | - |  |
| Income country | *Not selected* |  | | >0 | 1 |  |
| Risk of bias | - | - | | *Not selected* |  |  |
| Interactions with activity | | | | | | |
| Community | *Not selected* |  | | <0 | Strong |  |
| Individual | *Not selected* |  | | <0 | Strong |  |
| Electronic | - | - | | >0 | Reasonable |  |
| Multi-strategy | *Not selected* |  | | <0 | Reasonable |  |
| Duration | >0 | Strong | | >0 | Strong | Robust |
| Fun factor | - | - | | *Not selected* |  |  |
| Income country | >0 | Strong | | >0 | Strong | Robust |
| Risk of bias | - | - | | >0 | Strong |  |

# Combination of indicator values

The best (most beneficial) and worst (least beneficial) combination of indicator values are shown in **Table S5** and **Table S6** respectively.

**Table S5.** The indicator values associated with the minimum (most negative) mean difference predicted from our primary (random-effects) model. We show the most beneficial combination of indicator values for each of the two age groups (5-11 years and 12-18 years) and the two levels of country income status (high and non-high). We find the same combination regardless of whether we specify follow-up time (short, medium or long) or SES (mixed or low). The resulting predicted mean difference in each scenario is shown at the bottom of the table. For a given age group and income status, the largest beneficial effect is obtained for medium term follow-up and mixed SES (or universal interventions; highlighted in bold). Overall, the model predicts the greatest benefit for the older age group in non-high-income countries (indicated with an asterisk).

| Indicator | 12-18 years | | 5-11 years | |
| --- | --- | --- | --- | --- |
|  | **Non-high income** | **High income** | **Non-high income** | **High income** |
| School | Yes | Yes | Yes | Yes |
| Home | No | No | No | No |
| Community | No | No | No | No |
| Individual | Yes | Yes | Yes | Yes |
| Electronic | No | No | No | Yes |
| Diet and physical activity | No | No | No | Yes |
| Physical activity | Yes | Yes | Yes | No |
| Multi-strategy | Yes | Yes | Yes | Yes |
| Duration | Short | Short | Short | Short |
| Intensity | High | High | High | High |
| Integration | No | No | Yes | Yes |
| Flexibility/choice | No | No | No | No |
| Fun factor | Boring/Neutral | Boring/Neutral | Boring/Neutral | Fun |
| Resonance | Yes | Yes | No | No |
| Participation | Yes | Yes | Yes | Yes |
| Education | No | No | No | No |
| Social environment | No | No | No | No |
| Physical environment | No | No | No | No |
| Risk of bias | Low/some concerns | Low/some concerns | Low/some concerns | High |
| Predicted mean difference (zBMI): | | | | |
| Short: Low SES | -0.548 | -0.228 | -0.310 | -0.199 |
| Mixed SES | -0.569 | -0.249 | -0.332 | -0.220 |
| Medium: Low SES | -0.584 | -0.264 | -0.347 | -0.235 |
| Mixed SES | **-0.605*** | **-0.285** | **-0.368** | **-0.256** |
| Long: Low SES | -0.567 | -0.247 | -0.330 | -0.218 |
| Mixed SES | -0.588 | -0.268 | -0.351 | -0.239 |

**Table S6.** The indicator values associated with the maximum (most positive) mean difference predicted from our primary (random-effects) model. We show the least beneficial combination of indicator values for each of the two age groups (5-11 years and 12-18 years) and the two levels of country income status (high and non-high). We find the same combination regardless of whether we specify follow-up time (short, medium or long) or SES (mixed or low). The resulting predicted mean difference in each scenario is shown at the bottom of the table. For a given age group and income status, the least beneficial effect is obtained for short term follow-up and low SES. Overall, the model predicts the least benefit for the younger age group high-income countries (indicated with an asterisk). Indicators that take the same value in the best combination (Table S5) are highlighted in yellow.

| Indicator | 12-18 years | | 5-11 years | |
| --- | --- | --- | --- | --- |
|  | **Non-high income** | **High income** | **Non-high income** | **High income** |
| School | No | No | No | No |
| Home | Yes | Yes | Yes | Yes |
| Community | Yes | Yes | Yes | Yes |
| Individual | No | No | No | No |
| Electronic | Yes | Yes | No | Yes |
| Diet and physical activity | Yes | No | Yes | No |
| Physical activity | No | Yes | No | Yes |
| Multi-strategy | No | No | No | No |
| Duration | Long | Long | Long | Long |
| Intensity | Low | Low | Low | Low |
| Integration | Yes | Yes | No | No |
| Flexibility/choice | Yes | Yes | Yes | Yes |
| Fun factor | Boring/Neutral | Fun | Boring/Neutral | Fun |
| Resonance | Yes | No | Yes | Yes |
| Participation | Yes | Yes | Yes | Yes |
| Education | Yes | Yes | Yes | Yes |
| Social environment | Yes | Yes | Yes | Yes |
| Physical environment | Yes | Yes | Yes | Yes |
| Risk of bias | Low/some concerns | High | Low/some concerns | High |
| Predicted mean difference (zBMI): | | | | |
| Short: Low SES | **0.024** | **0.245** | **0.211** | **0.261*** |
| Mixed SES | 0.003 | 0.224 | 0.190 | 0.240 |
| Medium: Low SES | -0.013 | 0.209 | 0.175 | 0.225 |
| Mixed SES | -0.034 | 0.188 | 0.154 | 0.204 |
| Long: Low SES | 0.004 | 0.226 | 0.192 | 0.242 |
| Mixed SES | -0.017 | 0.205 | 0.171 | 0.221 |

# Sensitivity analysis: separate analyses of different outcome scales

Here we show the results of the sensitivity analyses with different outcome scales analysed separately, i.e., excluding any mapped data. The analysis with zBMI outcomes alone (**Figure S9**) is based on 171 observations from 110 trials while the BMI only analysis (**Figure S10**) involves 182 observations from 129 trials. In **Figure S11** we also show the results with zBMI and mapped percentile (excluding mapped BMI and proportions) which includes an additional 25 observations from 18 trials compared with the zBMI only analysis.

On the whole, the results for BMI only are similar to the results of the primary analysis, while the zBMI only results are largely uncertain. The heterogeneity parameter is estimated to be smaller in the analyses of individual outcomes than in the full dataset. We summarize the comparisons between the results for the separate outcome scales in **Table S*7*** and **Table S*8***.

We begin by inspecting the effects with probability $\geq$0.95 in either the random-effects analysis or the fixed effects analysis and probability $\geq$0.9 in the other (those considered ‘robust’ in Section J). There is strong evidence that environmental components are less beneficial in all three sensitivity analyses. The beneficial effects of physical activity alone and medium-term follow-up are also corroborated by the separate analyses, but with stronger evidence from BMI and weaker evidence (in the same direction) from zBMI. However, while there is no evidence of an effect of physical activity in the combined zBMI and percentile analysis, the direction is reversed. The observation that interventions are more effective in the older age group appears to be dominated by the BMI data ($P\left( <0 \right)=1$), with no evidence of an age effect from zBMI only ($P\left( <0 \right)=0.31$) or the combined analysis of zBMI and mapped percentile ($P\left( <0 \right)=0.23$).

Of the effects with reasonable evidence (probabilities $\geq$0.9) in the primary analysis, the beneficial effect of integration and the less beneficial effect of home-based activities are supported by the zBMI alone and combined zBMI and percentile analyses, but with no evidence from BMI alone. The beneficial effect of multi-component strategies is in the same direction in all analyses with strong evidence from zBMI alone ($P\left( <0 \right)=0.96$) and combined zBMI and percentile ($P\left( <0 \right)=0.95$), and slightly weaker evidence from BMI only ($P\left( <0 \right)=0.83$). The only result pointing in opposite directions is for the average effect of high-income countries: BMI strongly indicates a preference for high-income countries ($P\left( <0 \right)=1$), while zBMI finds greater beneficial effects for low-income countries ($P\left( >0 \right)=0.95$). This may be due to a lack of data from low-income countries. The strength of this evidence for the zBMI analysis is increased with the addition of the mapped percentile data ($P\left( >0 \right)=0.98$).

For the interaction terms, we observe no opposing evidence between the analyses on separate outcome scales. However, most of the effects observed in the primary analysis are dominated by one (or two) outcome scales with no evidence on the other(s). For example, the interactions between age and combined diet and physical activity, age and integration, age and income status of country, and activity and income status of country are observed in the BMI only analysis, but there is no evidence of these interactions from zBMI either alone or combined with percentile. Conversely, the interaction between activity and duration is observed in the same direction in all three analyses with the strongest evidence from zBMI and percentile ($P\left( >0 \right)=0.78$), followed by zBMI alone ($P\left( >0 \right)=0.69$) and BMI alone ($P\left( >0 \right)=0.59$).

The only interaction that appears with strong evidence in one of the separate outcome analyses that does not appear in the primary analysis is between interventions with an electronic component and interventions targeting both diet and physical activity. There is strong evidence of a synergistic effect in the BMI only analysis ($P\left( <0 \right)=0.97$), and weaker evidence in the same direction for combined zBMI and percentile ($P\left( <0 \right)=0.89$) and the zBMI only analysis ($P\left( <0 \right)=0.58$).

## zBMI only


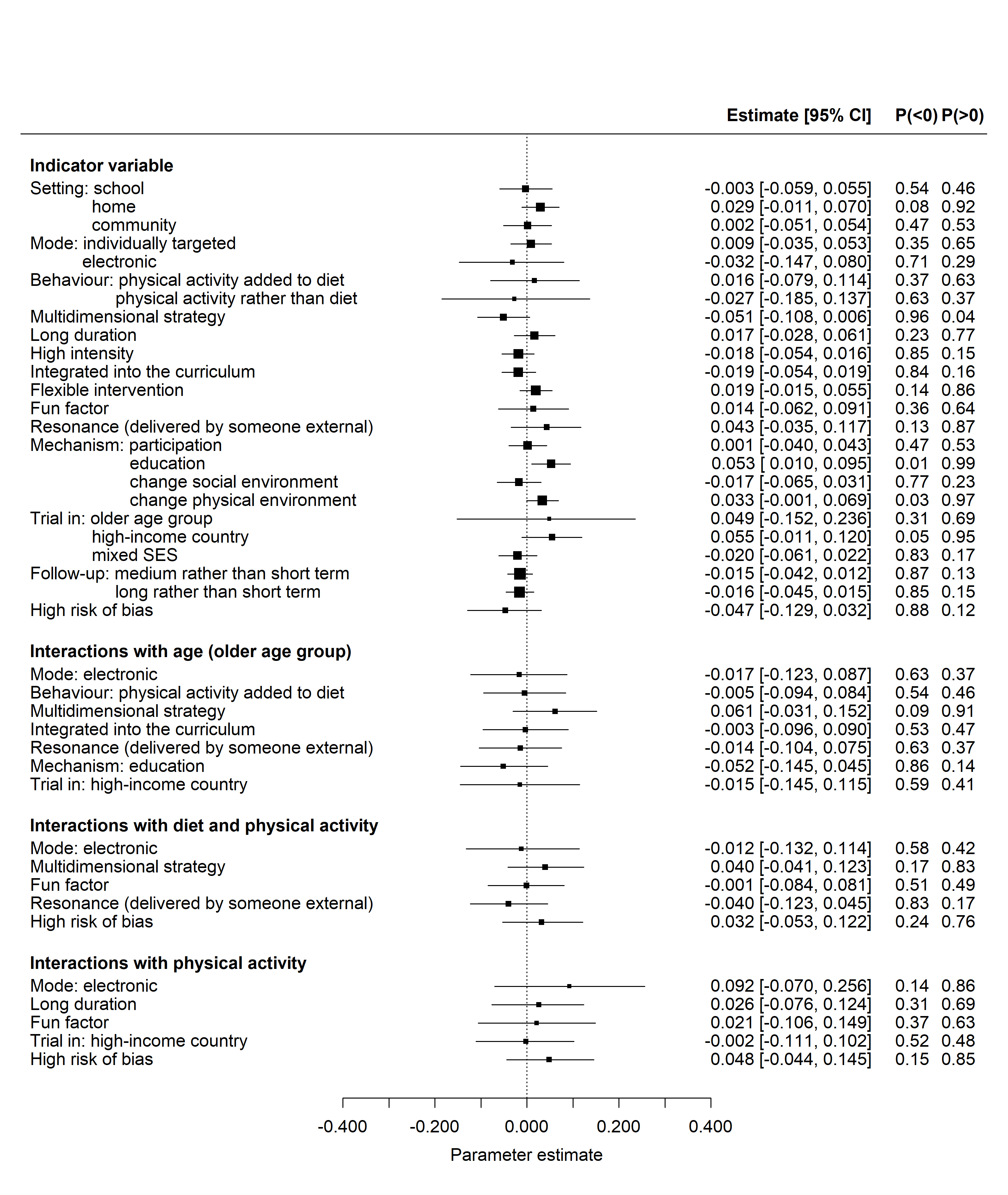


**Figure S9:** Parameter estimates from the random effects model fitted to data on reported zBMI only. The estimates of the intercept (for centred data) and heterogeneity parameter are $\alpha\left[ 95\% CI \right]=-0.045[-0.061,-0.029]$ and $\tau\left[ 95\% CI \right]=0.049 [0.037, 0.063]$ respectively.

## BMI only


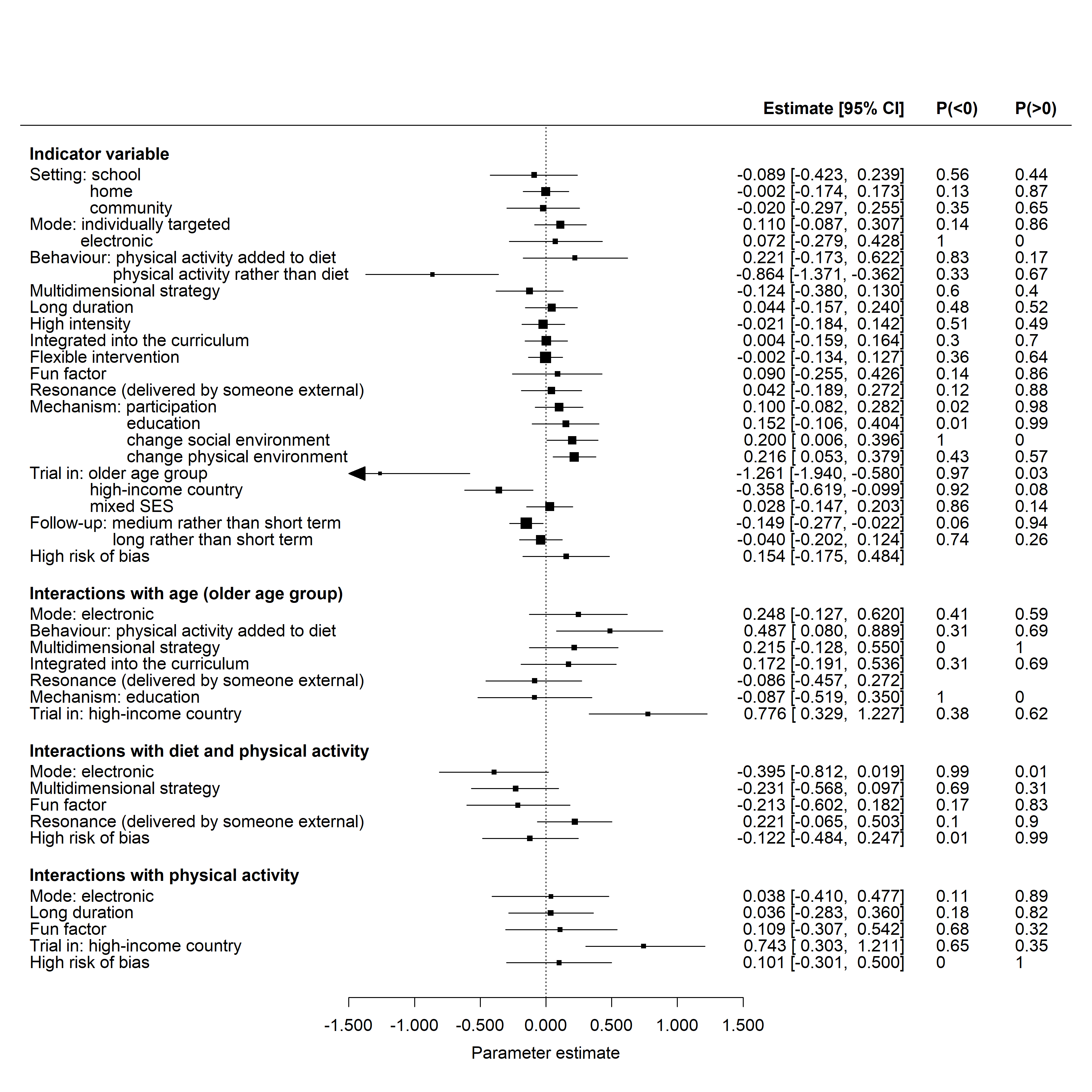


**Figure S10:** Parameter estimates from the random effects model fitted to data on reported zBMI only. The estimates of the intercept (for centred data) and heterogeneity parameter are $\alpha\left[ 95\% CI \right]=-0.05=-0.060[-0.133,-0.015]$ and $\tau\left[ 95\% CI \right]=0.271 [0.219, 0.332]$ respectively.

## zBMI and percentile only


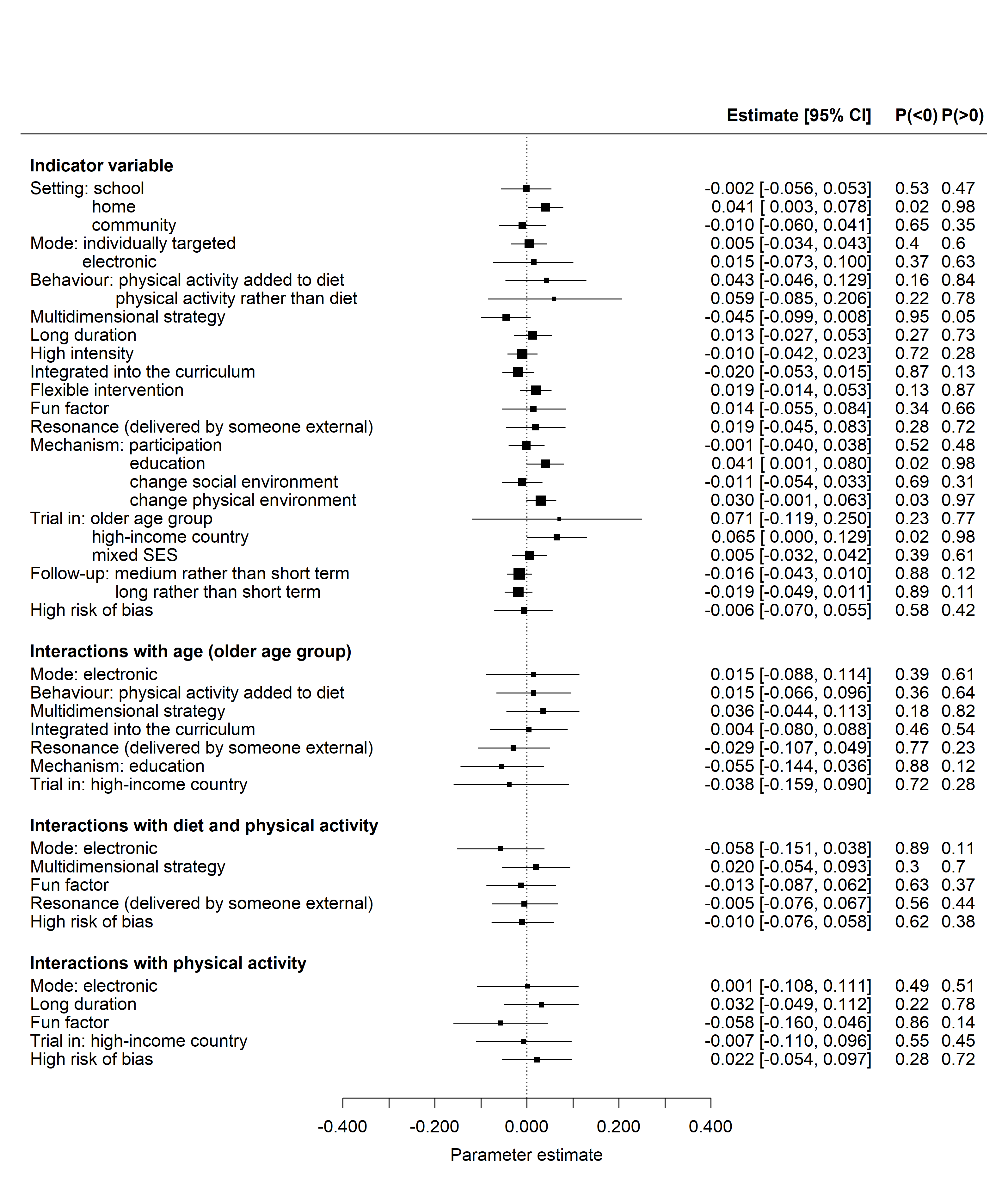


**Figure S11:** Parameter estimates from the random effects model fitted to data on reported zBMI and mapped percentile (excluding mapped BMI). The estimates of the intercept (for centred data) and heterogeneity parameter are $\alpha\left[ 95\% CI \right]=-0.045[-0.060,-0.029]$ and $\tau\left[ 95\% CI \right]=0.051 [0.039, 0.065]$ respectively.

## Comparison of different outcome scales

**Table S7:** A table summarizing the main differences between the indicator parameters estimated from the analyses on different outcome scales. The primary analysis includes all the reported zBMI data as well as the BMI and percentile data mapped onto the zBMI scale. We list the probability that a parameter estimate falls one side of zero in each analysis (with the corresponding direction indicated in the second column).

| Indicator | Direction of effect (in main analysis) | Probabilities in different analyses | | | |
| --- | --- | --- | --- | --- | --- |
|  |  | **Primary** | **BMI only** | **zBMI only** | **zBMI and Percentile** |
| Home | Less beneficial, $P\left( >0 \right)$ | 0.95 | 0.49 | 0.92 | 0.98 |
| Physical activity | Beneficial, $P\left( <0 \right)$ | 1 | 1 | 0.63 | 0.22 |
| Multi-strategy | Beneficial, $P\left( <0 \right)$ | 0.92 | 0.83 | 0.96 | 0.95 |
| Integrated | Beneficial, $P\left( <0 \right)$ | 0.93 | 0.48 | 0.84 | 0.87 |
| Social | Less beneficial, $P\left( >0 \right)$ | 0.77 | 0.98 | 0.23 | 0.31 |
| Environment | Less beneficial, $P\left( >0 \right)$ | 1 | 0.99 | 0.97 | 0.97 |
| Older age group | Beneficial, $P\left( <0 \right)$ | 1 | 1 | 0.31 | 0.23 |
| High income country | Beneficial, $P\left( <0 \right)$ | 0.95 | 1 | 0.05 | 0.02 |
| Medium term | Beneficial, $P\left( <0 \right)$ | 0.99 | 0.99 | 0.87 | 0.88 |

**Table S8:** A table summarizing the main differences between the interactions estimated from the analyses on different outcome scales. The primary analysis includes all the reported zBMI data as well as the BMI and percentile data mapped onto the zBMI scale. We list the probability that an interaction estimate falls one side of zero in each analysis (with the corresponding direction indicated in the second column).

| Interaction | Direction of effect (in main analysis) | Probabilities in different analyses | | | |
| --- | --- | --- | --- | --- | --- |
|  |  | **Primary** | **BMI only** | **zBMI only** | **zBMI and Percentile** |
| Older age & Diet and physical activity | Antagonistic, $P\left( >0 \right)$ | 0.99 | 0.99 | 0.46 | 0.64 |
| Older age & integration | Antagonistic, $P\left( >0 \right)$ | 0.94 | 0.82 | 0.47 | 0.54 |
| Older age & high income country | Antagonistic, $P\left( >0 \right)$ | 1 | 1 | 0.41 | 0.28 |
| Diet and physical activity & electronic | Synergistic, $P\left( <0 \right)$ | 0.84 | 0.97 | 0.58 | 0.89 |
| Physical activity & long duration | Antagonistic, $P\left( >0 \right)$ | 0.97 | 0.59 | 0.69 | 0.78 |
| Physical activity & high income country | Antagonistic, $P\left( >0 \right)$ | 1 | 1 | 0.48 | 0.45 |

# Sensitivity analysis: assuming different correlations between repeated measures over time

In **Figure S12** and **Figure S13** we show the results of the random effects analysis assuming correlations of 0.5 and 0.95 between observations at different time points. We observe negligible differences from the primary analysis across all estimated effects. The estimate of the heterogeneity parameter is smaller for the 0.5 correlation ($0.076 [0.063, 0.091]$) and larger for the 0.95 correlation ($0.088 [0.077, 0.010]$).

## Correlation of 0.5


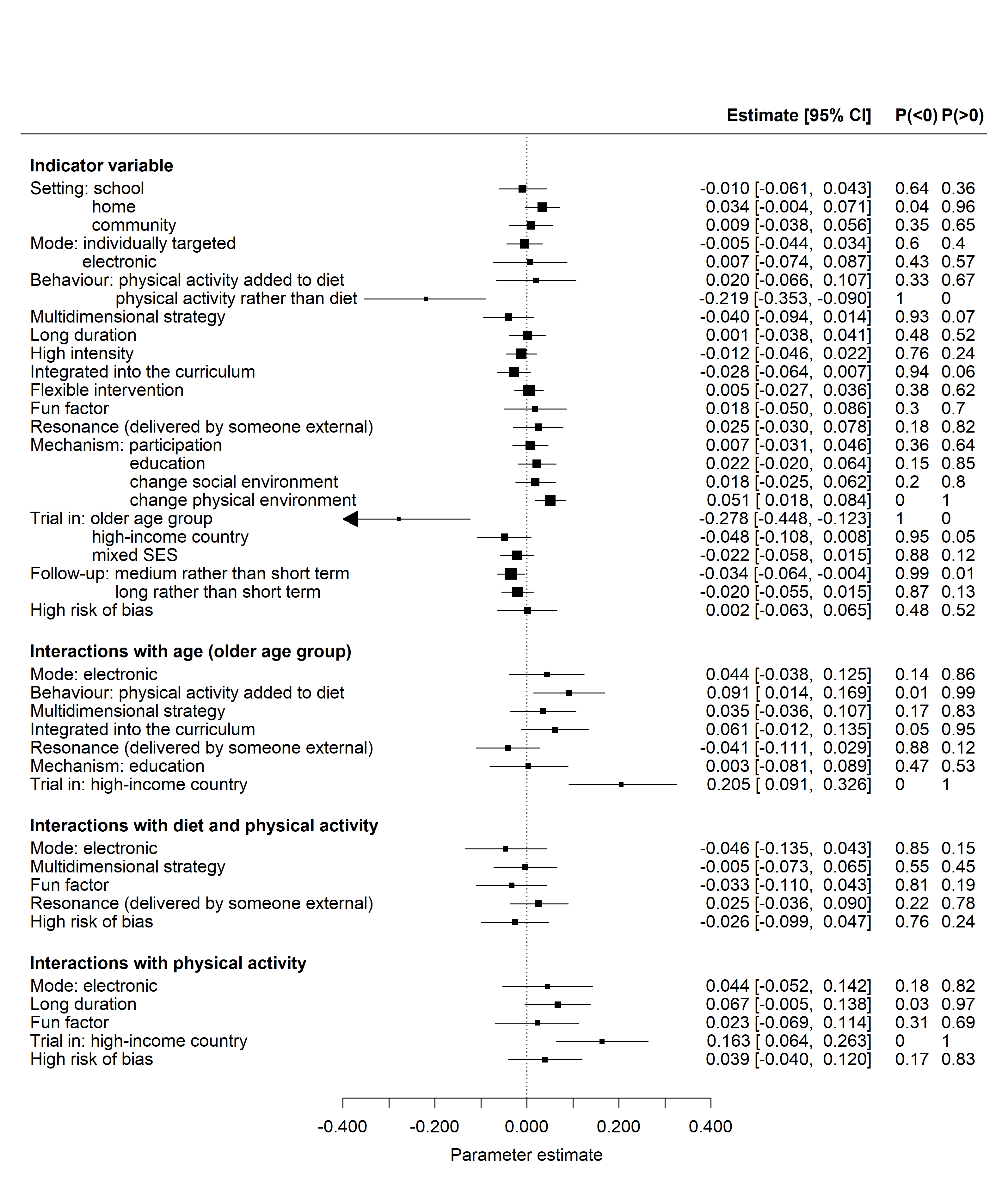


**Figure S12:** Parameter estimates from the random effects model assuming correlations of$\rho_{y,tt^{'}}=\rho_{d,tt^{'}}=0.5$ for one degree of separation (short to medium, and medium to long). The estimates of the intercept (for centred data) and heterogeneity parameter are $\alpha\left[ 95\% CI \right]=-0.037 [-0.052, -0.022]$ and $\tau\left[ 95\% CI \right]=0.076 [0.063, 0.091]$ respectively.

## Correlation of 0.95


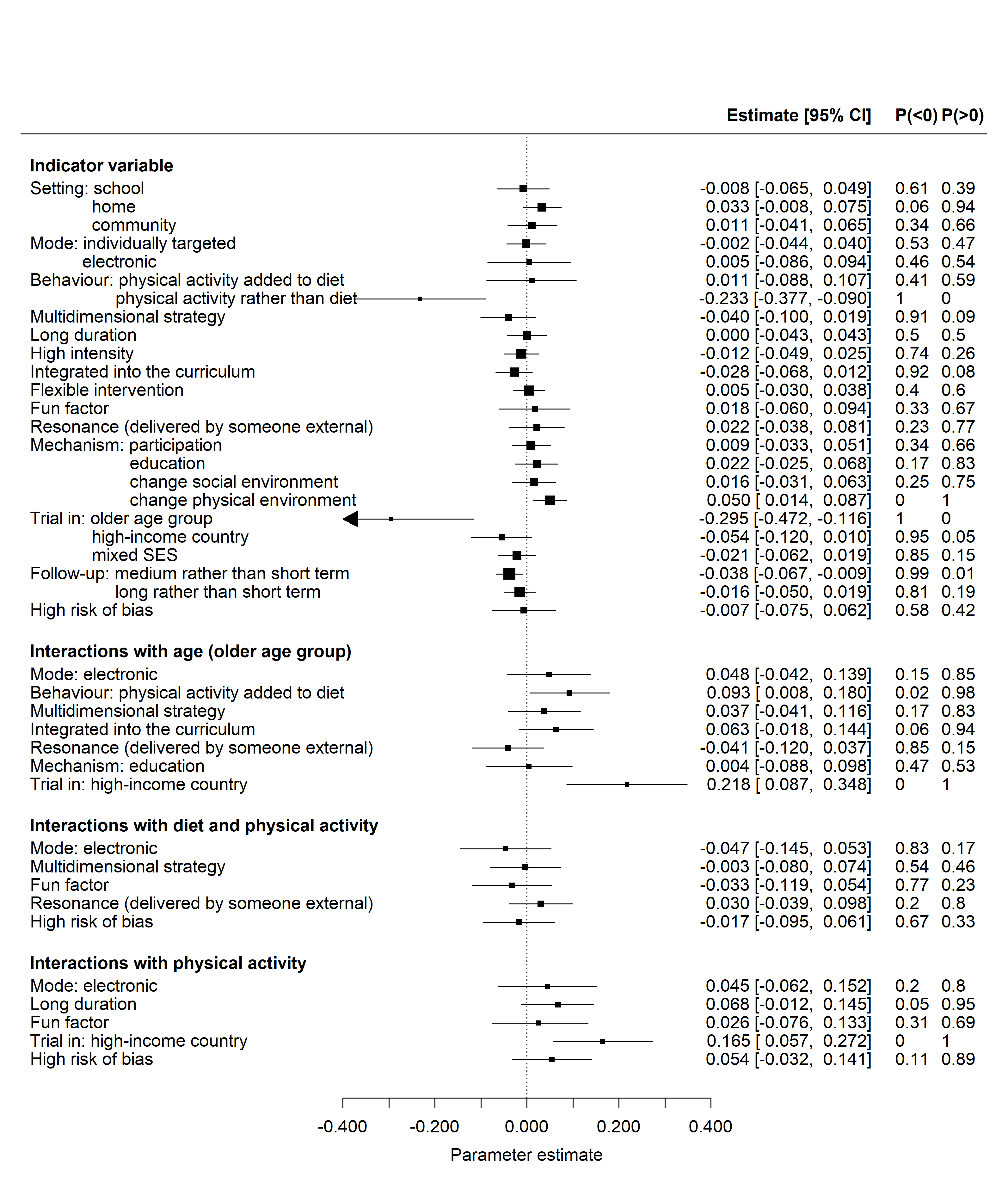


**Figure S13:** Parameter estimates from the random effects model assuming correlations of$\rho_{y,tt^{'}}=\rho_{d,tt^{'}}=0.95$ for one degree of separation (short to medium, and medium to long). The estimates of the intercept (for centred data) and heterogeneity parameter are $\alpha\left[ 95\% CI \right]=-0.037 [-0.053, -0.021]$ and $\tau\left[ 95\% CI \right]=0.088 [0.077, 0.010]$ respectively.

# References for supplementary materials

1. Spiga, F., et al., *A novel analytic framework to investigate differential effects of interventions to prevent obesity in children and young people*. 2024: medrXiv. p. 1-24.

2. Spiga, F., et al., *Interventions to prevent obesity in children aged 5 to 11 years old*. 2024: Cochrane Database of Systematic Reviews TBD.

3. Spiga, F., et al., *Interventions to prevent obesity in children aged 12 to 18 years old*. 2024: Cochrane Database of Systematic Reviews TBD.

4. Davies, A.L. and J.P.T. Higgins, *A complex meta-regression model to identify effective features of interventions from multi-arm, multi-follow-up trials*. 2024: arXiv. p. 1-22.

5. Plummer, M., *JAGS Version 4.3.0 User Manual*. 2017: Online: Available from <https://martynplummer.wordpress.com/>. Accessed January 2024.

6. Brooks, S.P. and A. Gelman, *General Methods for Monitoring Convergence of Iterative Simulations.* Journal of Computational and Graphical Statistics, 1998. **7**(4): p. 434-455.

7. Andrew, G. and B.R. Donald, *Inference from Iterative Simulation Using Multiple Sequences.* Statistical Science, 1992. **7**(4): p. 457-472.

8. Efthimiou, O., et al., *Bayesian models for aggregate and individual patient data component network meta-analysis.* Stat Med, 2022. **41**(14): p. 2586-2601.

9. Spiegelhalter, D.J., et al., *Bayesian Measures of Model Complexity and Fit.* Journal of the Royal Statistical Society Series B: Statistical Methodology, 2002. **64**(4): p. 583-639.
