# Supplementary material for: Factors associated with the effectiveness of interventions to prevent obesity in children: a synthesis of evidence from 204 randomised trials
Source: BMJ Public Health. 2025 May 12;3(1):e001707. doi: 10.1136/bmjph-2024-001707 (PMC12086980; doi:10.1136/bmjph-2024-001707)
Supplement: Supplementary file 2 [file bmjph-3-1-s001.pdf]

Supplementary material for “Factors associated with effectiveness of interventions to prevent obesity in children: a synthesis of evidence synthesis from 204 randomized trials”

Contents

A Coding of binary intervention indicators 3

B Mathematical details of the complex synthesis model 5

B.1 Random effects model 5

B.2 Fixed effects model 6

C Imputing correlation coefficients to specify the within study covariance matrix 7

D Implementation of model in JAGS 7

E Selection of interactions via stepwise stochastic search variable selection (SSVS) models 8

E.1 SSVS model 8

E.2 Step-wise SSVS procedure 8

E.3 Deviations from our analysis plan (indicator selection) 9

F Adjusting the intercept 9

G Study characteristics 11

H Selecting indicator variables 27

I Selecting interactions via SSVS 29

I.1 Primary analysis (random effects) 30

I.1.1 Step 1: No interactions 30

I.1.2 Step 2: Interactions with age 31

I.1.3 Step 3: Interactions with change in behaviour targeted 32

I.2 Secondary analysis (fixed effects) 33

I.2.1 Step 1: No interactions 33

I.2.2 Step 2: Interactions with age 34

I.2.3 Step 3: interactions with change in behaviour targeted 35

J Comparing random and fixed effects models 36

K Combination of indicator values 38

L Sensitivity analysis: separate analyses of different outcome scales 40

L.1 zBMI only 41

L.2 BMI only 42

L.3 zBMI and percentile only 43

L.4 Comparison of different outcome scales 44
M Sensitivity analysis: assuming different correlations between repeated measures over time 45
M.1 Correlation of 0.5 45
M.2 Correlation of 0.95 46
References for supplementary materials 47

A Coding of binary intervention indicators

Below we list the (dichotomous) intervention indicators defined by the analytic framework and how they were coded. For details of how the questions were answered, we refer to [1]. We describe any dichotomizations applied to continuous or categorical variables and give the resulting split of the data (where we write n for the number of interventions that fall into that category). The numbers refer to the full set of coded interventions ($n = 255$) as reported in [1] rather than the final set of interventions included in the analysis ($n = 250$, see Table 1 in the main paper).

1. School: was the intervention delivered in a school (in full or in part)?
    - = 1 if Yes ($n = 180$)
    - = 0 if No ($n = 75$)
2. Home: was the intervention delivered in the home (in full or in part)?
    - = 1 if Yes ($n = 47$)
    - = 0 if No ($n = 208$)
3. Community: was the intervention delivered in the community or other non-school/non-home setting (in full or in part)?
    - = 1 if Yes ($n = 72$)
    - = 0 if No ($n = 183$)
4. Home activity: did the intervention include a home activity for the child?
    - = 1 if Yes ($n = 91$)
    - = 0 if No ($n = 164$)
5. Group: Was the intervention delivered to the child as part of a group (in full or in part)?
    - = 1 if Yes ($n = 211$)
    - = 0 if No ($n = 44$)
6. Individual: Was the intervention delivered to the child individually (in full or in part)?
    - = 1 if Yes ($n = 122$)
    - = 0 if No ($n = 133$)
7. Electronic: was the intervention delivered to the child electronically?
    - = 1 if Yes exclusively/Yes significantly/Yes as a minor component ($n = 54$)
    - = 0 if No ($n = 201$)
8. Diet: Did the intervention aim to change diet?
    - = 1 if Yes exclusively/Yes substantially ($n = 187$)
    - = 0 if No/Yes minimally ($n = 68$)
9. Physical activity: Did the intervention aim to change physical activity?
    - = 1 if Yes exclusively/Yes substantially ($n = 207$)
    - = 0 if No/Yes minimally ($n = 48$)
10. Multi-strategy: Did the intervention use multiple strategies (three or more)?

- = 1 if Yes ($n = 161$)
- = 0 if No ($n = 94$)

11. Single phase: was the intervention applied in a single phase?
    - = 1 if Yes ($n = 207$)
    - = 0 if No ($n = 48$)
12. Continuous: was the intervention applied continuously?
    - = 1 if Yes ($n = 246$)
    - = 0 if No ($n = 9$)
13. Total duration: what was the total duration of the intervention?
    - = 1 if Long (longer than median total duration, ≥ 30.33 weeks) ($n = 128$)
    - = 0 if Short (shorter than median total duration, < 30.33 weeks) ($n = 127$)
14. Peak duration: what was the duration of the period of peak engagement with the intervention?
    - = 1 if Long (longer than median peak duration, ≥ 25.98 weeks) ($n = 131$)
    - = 0 if Short (shorter than median peak duration, < 25.98 weeks) ($n = 124$)
15. Intensity: what was the level of engagement with the children during the peak period?
    - = 1 if High ($n = 152$)
    - = 0 if Low ($n = 103$)
16. Integration: was the intervention integrated into the normal curriculum/ habits?
    - = 1 if Yes ($n = 121$)
    - = 0 if No/Partially ($n = 134$)
17. Flexibility: was the intervention designed to be implemented in a flexible manner/tailored to specific participants?
    - = 1 if Yes ($n = 86$)
    - = 0 if No ($n = 169$)
18. Choice: was choice for the child of physical activity/diet designed into the intervention?
    - = 1 if Yes ($n = 66$)
    - = 0 if No ($n = 189$)
19. Fun factor: was the intervention considered fun?
    - = 1 if Fun ($n = 154$)
    - = 0 if Neutral/Not fun ($n = 101$)
20. Resonance: was the intervention experienced by children via someone external or unusual?
    - = 1 if Yes ($n = 134$)
    - = 0 if No ($n = 121$)
21. Participation: did the intervention have an explicit component that requires the child to participate?
    - = 1 if Yes ($n = 170$)

- = 0 if No ($n = 85$)

22. Education: did the intervention have an explicit component of education/information provision for the child?
    - = 1 if Yes ($n = 190$)
    - = 0 if No ($n = 65$)
23. Social: did the intervention have an explicit component aiming to change the social environment of the child (e.g., at school or home)?
    - = 1 if Yes ($n = 175$)
    - = 0 if No ($n = 80$)
24. Environment: did the intervention have an explicit component aiming to change the physical environment of the child (e.g., at school or home)?
    - = 1 if Yes ($n = 79$)
    - = 0 if No ($n = 176$)
25. Commercial: were commercial interests involved in the intervention/trial?
    - = 1 if Yes ($n = 27$)
    - = 0 if No ($n = 228$)

B Mathematical details of the complex synthesis model

B.1 Random effects model

Each trial i with A_i arms and T_i follow-up times provides a $T_i(A_i - 1) \times 1$ vector of observations $y_i = \left(y_{i,1}, y_{i,2}, \ldots, y_{i,T_i}\right)^\top = \left(y_{i,1}^{(1)}, \ldots, y_{i,1}^{(A_i-1)}, \ldots, y_{i,T_i}^{(1)}, \ldots, y_{i,T_i}^{(A_i-1)}\right)^\top$. Each $y_{i,t}^{(k)}$ is a mean difference in change from baseline to time t between arm k and some trial-specific reference arm r. The reference arm is either a control arm, $r = C$, or another active intervention, $r = A_i$. As described in our Cochrane reviews [2, 3], we calculated mean differences from arm-level data selected in the following order of preference: (i) follow-up means adjusted for baseline values, (ii) mean change from baseline (change scores), (iii) unadjusted baseline and follow-up means, (iv) unadjusted follow-up means without baseline data. For scenario (iii) we used the baseline and follow-up values to first calculate change from baseline. To obtain standard errors on these change scores we assumed a correlation coefficient of 0.9 between baseline and follow-up based on observed correlations in other trials. This is different from the value of 0.95 we used in our Cochrane reviews which was informed by initial observations of a subset of trials.

We define a meta-regression style model assuming additive effects of indicator variables (covariates) on three levels: study (z_i), intervention ($x_i^{(k)}$), and follow-up time ($w_{i,t}$). We also include pairwise interaction terms ($\mathcal{J}_{i,t}^{(k)}$) between indicators on any level. The form of the model depends on the reference arm of the trial and is summarized as follows,

$$\boldsymbol{y_i} \sim N(\boldsymbol{\delta_i}, \boldsymbol{V_i})$$
$$\boldsymbol{\delta_i} \sim N(\boldsymbol{\theta_i}, \boldsymbol{\Sigma_i})$$

$$\theta_{i,t}^{(k)} = \begin{cases} \alpha + \sum_{j=1}^{n} \beta_j x_{i,j}^{(k)} + \sum_{j=1}^{p} \gamma_j z_{i,j} + \sum_{j=1}^{q-1} \phi_j w_{i,jt} + \sum_{j=1}^{l} \eta_j \mathcal{J}_{i,jt}^{(k)} & \text{for } r = C \\ \sum_{j=1}^{n} \beta_j (x_{i,j}^{(k)} - x_{i,j}^{(r)}) + \sum_{j=1}^{l} \eta_j \left(\mathcal{J}_{i,jt}^{(k)} - \mathcal{J}_{i,jt}^{(k)}\right) & \text{for } r = A_i \end{cases}$$

$$\boldsymbol{V_i} = \begin{pmatrix} \boldsymbol{V_{i,1}} & \boldsymbol{V_{i,12}} & \cdots & \boldsymbol{V_{i,1T_i}} \\ \boldsymbol{V_{i,21}} & \boldsymbol{V_{i,2}} & \cdots & \boldsymbol{V_{i,2T_i}} \\ \vdots & \vdots & \ddots & \vdots \\ \boldsymbol{V_{i,T_i1}} & \boldsymbol{V_{i,T_i2}} & \cdots & \boldsymbol{V_{i,T_i}} \end{pmatrix}, \quad \boldsymbol{\Sigma_i} = \begin{pmatrix} \tau^2 & \tau^2/2 & \cdots & \tau^2/2 \\ \tau^2/2 & \tau^2 & \cdots & \tau^2/2 \\ \vdots & \vdots & \ddots & \vdots \\ \tau^2/2 & \tau^2/2 & \cdots & \tau^2 \end{pmatrix} \tag{1}$$

where the parameters $\beta = (\beta_1, \ldots, \beta_n)^\top$, $\gamma = (\gamma_1, \ldots, \gamma_p)^\top$, and $\phi = (\phi_1, \ldots, \phi_{q-1})^\top$ are the regression coefficients for the intervention, study-level, and follow-up time indicators respectively. The parameters $\eta = (\eta_1, \ldots, \eta_l)^\top$ are the regression coefficients for the interaction parameters.

The $T_i(A_i - 1) \times T_i(A_i - 1)$ covariance matrix $\boldsymbol{V_i}$ captures correlations between measurements made in the same trial (due to multiple arms and/or follow-up times) and is assumed to be known. The diagonal elements of the covariance matrix are equal to the observed variance (squared standard error) of the measurements $y_{i,t}^{(k)}$. The off-diagonal elements capture correlations between multiple observations in the same trial and depend on two (imputed) correlation coefficients $\rho_{y,tt'}$ and $\rho_{d,tt'}$ (described in Section C). We refer to our development paper [4] for details of this matrix.

For the random effects model, we assume the trial-specific effects $\delta_{i,t}^{(k)}$ are normally distributed with covariance matrix $\boldsymbol{\Sigma_i}$ (dimensions $T_i(A_i - 1) \times T_i(A_i - 1)$). This matrix depends on the heterogeneity variance, τ^2, which we assume captures the between-trial variance for all arms and all follow-up times.

B.2 Fixed effects model

As our secondary analysis we define a fixed effects model by setting $\tau = 0$ in Equation (1). This yields

$$\boldsymbol{y_i} \sim N(\boldsymbol{\theta_i}, \boldsymbol{V_i})$$

$$\theta_{i,t}^{(k)} = \begin{cases} \alpha + \sum_{j=1}^{n} \beta_j x_{i,j}^{(k)} + \sum_{j=1}^{p} \gamma_j z_{i,j} + \sum_{j=1}^{q-1} \phi_j w_{i,jt} + \sum_{j=1}^{l} \eta_j \mathcal{J}_{i,jt}^{(k)} & \text{for } r = C \\ \sum_{j=1}^{n} \beta_j (x_{i,j}^{(k)} - x_{i,j}^{(r)}) + \sum_{j=1}^{l} \eta_j \left(\mathcal{J}_{i,jt}^{(k)} - \mathcal{J}_{i,jt}^{(k)}\right) & \text{for } r = A_i \end{cases}$$

$$\boldsymbol{V_i} = \begin{pmatrix} \boldsymbol{V_{i,1}} & \boldsymbol{V_{i,12}} & \cdots & \boldsymbol{V_{i,1T_i}} \\ \boldsymbol{V_{i,21}} & \boldsymbol{V_{i,2}} & \cdots & \boldsymbol{V_{i,2T_i}} \\ \vdots & \vdots & \ddots & \vdots \\ \boldsymbol{V_{i,T_i1}} & \boldsymbol{V_{i,T_i2}} & \cdots & \boldsymbol{V_{i,T_i}} \end{pmatrix}. \tag{2}$$

C Imputing correlation coefficients to specify the within study covariance matrix

As described in Section B, the within-study covariance matrix in our model depends on two correlation coefficients $\rho_{y,tt'}$ and $\rho_{d,tt'}$. The former is the correlation between observations of mean difference for a given intervention (relative to the reference arm) at time points t and t', assumed to be common to all arms. The latter is the correlation between observations of the change score in the reference arm between t and t'.

To specify the within-study covariance matrix, we imputed sensible values for the correlations $\rho_{y,tt'}$ and $\rho_{d,tt'}$ based on observed correlations in the data. Using multi-follow-up trials, we plotted mean differences and change scores in control arms for different combinations of time points: (i) short vs medium term, (ii) short vs long term, and (iii) medium vs long term. We then calculated correlation coefficients for each plot.

To simplify the imputation we assumed that time points with one degree of separation, (i) and (iii), have the same correlation, ρ_1. We also assumed that correlations act multiplicatively across time such that time points with two degrees of separation, (ii), have the square of this correlation, $\rho_2 = \rho_1^2$.

For mean differences, $\rho_{y,tt'}$, we found a correlation of $\rho_1 = 0.77$ for short vs medium term (based on $m = 31$ data points), $\rho_2 = 0.83$ ($\rho_1 = \sqrt{\rho_2} = 0.91$) for short vs long term ($m = 13$), and $\rho_1 = 0.86$ for medium vs long term ($m = 45$). This gives an overall weighted mean of $\rho_1 = 0.82$.

For change scores, $\rho_{d,tt'}$, we found a correlation of $\rho_1 = 0.69$ for short vs medium term ($m = 24$), $\rho_2 = 0.77$ ($\rho_1 = \sqrt{\rho_2} = 0.88$) for short vs long term ($m = 9$), and $\rho_1 = 0.84$ for medium vs long term ($m = 34$). This gives an overall weighted mean of $\rho_1 = 0.77$.

Therefore, we chose to use $\rho_1 = 0.8$ for both mean differences and change scores with one degree of separation. To cover the range of correlations observed, we chose to perform sensitivity analyses with $\rho_1 = 0.5$ and $\rho_1 = 0.95$.

D Implementation of model in JAGS

We implemented our models in a Bayesian framework using JAGS [5]. We used four chains and assessed convergence by inspecting MCMC trace plots and using the Brooks-Gelman-Rubin $\hat{R}$ statistic [6, 7]. For all non SSVS models (see Section E) we assigned non-informative prior distributions to all parameters. For heterogeneity, we specified $\tau \sim \mathrm{Unif}(0,5)$. For the intercept and all regression coefficients we used $N(0, 100^2)$. An upper limit of 5 for heterogeneity and a standard deviation of 100 is large on the zBMI scale (which follows a standard normal in the general population). We used an adaptive phase of 10,000 iterations, a burn in of an additional 40,000, and a further 30,000 iterations from which we drew our posterior samples.

All code required to implement our analysis is provided in the GitHub repository here:
https://github.com/AnnieDavies/Obesity_Synthesis

E Selection of interactions via stepwise stochastic search variable selection (SSVS) models

E.1 SSVS model

To select interaction terms $\mathcal{J}_{i,jt}^{(k)}$, we use a Bayesian stochastic search variable selection (SSVS) model from Efthimiou et al [8]. While fitting the model, interactions are selected at each iteration of the MCMC using indicator variables $I_j \in \{0,1\}$ in the prior distributions of the interaction coefficients η_j. The priors are defined as the sum of two normal distributions. The value of the indicator variable at each iteration specifies which of these distributions to use. The first is very narrowly distributed around zero and is used when $I_j = 0$ (the jth interaction term is not selected). The second is wider, allowing for non-zero values of the coefficient and is used when $I_j = 1$ (the jth interaction is selected). By inspecting the posterior distribution of the indicator parameter for each interaction term j we can assess the proportion of times the interaction was selected. The more important an interaction, the more often it will be selected.

The SSVS version of our model is defined in the same way as in Section B but with the following prior distributions on the interaction coefficients,

$$\begin{aligned}
&\pi(\eta_j \mid I_j) = (1 - I_j)N(0, \xi^2) + I_j N(0, g^2\xi^2) \\
&I_j \sim \text{Bernoulli}(p_j), \qquad \xi \sim N(0, v_\xi), \\
&v_\xi = \text{small variance}, \quad g = \text{large number},
\end{aligned} \tag{3}$$

where $j = 1, \ldots, l$ and l is the number of interactions from which to select. For all other model parameters we use the non-informative prior distributions described in Section D. The variance v_ξ, which specifies the width of the narrow distribution, should be such that it only allows for effects that are `practically zero' relative to the scale of the data. The parameter g, which specifies the width of the wider distribution, should be large enough to allow for non-zero (but sensible) values of the parameter. Informed by [8], the scale of zBMI, and preliminary tests on our data, we chose $v_\xi = 10^{-3}$ and $g^2 = 100$.

In Equation (3), the indicator variables are assigned Bernoulli prior distributions with probabilities p_j. These probabilities can be used to define informative priors based on our beliefs about the importance of interaction terms. Otherwise, setting $p_j = 0.5\ \forall j$ defines non-informative (equi-probable) priors which choose the interactions based on information from the data alone. In our step-wise procedure described in Section E.2 we use the latter.

E.2 Step-wise SSVS procedure

As described in the main paper, we selected interaction terms for our model by applying the SSVS method in a step-wise process. Each step involved a different set of interaction terms from which to select. In the following we list the model specified at each step and give the implementation details used to fit it in JAGS [5].

i. Step 1: Model with no interactions

- We used an adaptive phase of $n_{\text{adapt}} = 10{,}000$ iterations, an additional burn in of $n_{\text{burn}} = 20{,}000$, followed by a further $n_{\text{samp}} = 30{,}000$ iterations from which we drew our posterior samples.

ii. Step 2: Model with interactions between age and all other indicator variables

- Implementation details: $n_{\text{adapt}} = 10{,}000$, $n_{\text{burn}} = 20{,}000$, $n_{\text{samp}} = 30{,}000$.

iii. Step 3: Model with interactions between 'change of behaviour targeted' (diet and/or physical activity) and all other indicators

- As described in Section 3.1 of the main paper, behaviour targeted was coded as two binary variables indicating whether the intervention targeted physical activity alone or a combination of diet and physical activity. An intervention targeting diet alone is then indicated by setting both these variables to zero.
- This model included interactions between each of these variables (physical activity alone and diet & physical activity) and every other indicator.
- Implementation details: $n_{\text{adapt}} = 10{,}000$, $n_{\text{burn}} = 40{,}000$, $n_{\text{samp}} = 30{,}000$.

To define our final model, we chose interaction terms that were selected more than 50% of the time in steps 2 and 3. We performed steps 1-3 separately for the random effects and fixed effect models.

E.3 Deviations from our analysis plan (indicator selection)

In our analysis plan we set out to use the SSVS method to select intervention indicators as well as interactions. However, the first two steps of our selection process (assessing collinearity and the proportion of identical responses) led to a manageable number of indicators (see Table 1 in the main paper) all of which were deemed important by our stakeholders. Therefore, we did not require the SSVS method to reduce the number of indicators further.

Our analysis plan also states that we would to assign informative probabilities to the SSVS prior distributions, describing the interactions that our stakeholders considered most and least probable. However, beyond expressing interest in interactions between age and behaviour targeted, our stakeholders encountered challenges in pinpointing particular interactions of interest. Additionally, preliminary analyses revealed that the use of informative priors led to the dominance of predetermined interactions, undermining our objective to select interactions based on the data.

F Adjusting the intercept

In Section 3.2.3 of the main manuscript we evaluated our primary model for every possible combination of indicator variables. Each combination involved inserting values of 0 or 1 for each indicator. However, the model intercept was calculated using indicators centred at their mean value. Therefore, to interpret the outcome of the model as a mean difference in change from baseline in zBMI we need to convert the intercept from the centred model to the intercept for the non-centred model.

Using the notation from Section B, the fixed effects of the non-centred (nc) model (for control comparison studies) is

$$\theta_{i,t}^{(k)} = \alpha_{nc} + \sum_{j=1}^{n} \beta_j x_{i,j}^{(k)} + \sum_{j=1}^{p} \gamma_j z_{i,j} + \sum_{j=1}^{q-1} \phi_j w_{i,jt} + \sum_{j=1}^{l} \eta_j \mathcal{J}_{i,jt}^{(k)}. \tag{4}$$

The equivalent model with centred (c) indicators is

$$\theta_{i,t}^{(k)} = \alpha_c + \sum_{j=1}^{n} \beta_j (x_{i,j}^{(k)} - \langle x_j \rangle) + \sum_{j=1}^{p} \gamma_j (z_{i,j} - \langle z_j \rangle) + \sum_{j=1}^{q-1} \phi_j (w_{i,jt} - \langle w_j \rangle) + \sum_{j=1}^{l} \eta_j (\mathcal{J}_{i,jt}^{(k)} - \langle \mathcal{J}_j \rangle), \tag{5}$$

where the angular brackets $\langle\, .\, \rangle$ denote the mean value of that indicator (or interaction) over all observations in the data set. The intercept reported in the main paper is an estimate of the centred intercept, α_c. To convert this to the non-centred intercept α_{nc} we equate right hand side of Equations (4) and (5), which leads to

$$\alpha_{nc} = \alpha_c - \left(\sum_{j=1}^{n} \beta_j \langle x_j \rangle + \sum_{j=1}^{p} \gamma_j \langle z_j \rangle + \sum_{j=1}^{q-1} \phi_j \langle w_j \rangle + \sum_{j=1}^{l} \eta_j \langle \mathcal{J}_j \rangle \right). \tag{6}$$

To obtain the predicted mean differences in Table 2 in the main paper, we calculated the intercept from Equation (6) using the mean values of each indicator variable used to centre the data, and the estimated intercept (α_c) and regression coefficients from our primary analysis. This gave $\alpha_{nc} = -0.0003$, which represents the effect of an intervention whose indicators are all equal to zero.

G Study characteristics

Table S1. Summary of the characteristics of the trials included in our analyses. For studies that provide data on multiple outcomes, we selected data in the following order of preference: (i) zBMI, (ii) percentile (mapped), (iii) BMI (mapped). The BMI-only sensitivity analysis included all studies that provided data on BMI.

**Studies that only provide contrast-level data on BMI and could not be mapped to zBMI. These studies were excluded from the main analysis but were included in the BMI-only sensitivity analysis.*

***Study that only provides contrast-level data on percentile and could not be mapped to zBMI. This study was excluded from all analyses.*

Study	Age group	Income status of country	Socio-economic status	Comparison(s)	Participants at baseline	Outcome	Follow-up time(s)	Risk of bias judgement(s)
Adab 2018	5-11	High	Low	Diet and physical activity vs control	Intervention(s): 660<br>Control: 732	zBMI	Long term	Some concerns
Amaro 2006	12-18	High	Mixed	Diet vs control	Intervention(s): 153<br>Control: 88	zBMI	Short term	Some concerns
Andrade 2014	12-18	Non-High	Mixed	Diet and physical activity vs control	Intervention(s): 687<br>Control: 692	zBMI<br>BMI	Long term	Some concerns
Annesi 2016	5-11	High	Mixed	Diet and physical activity vs control	Intervention(s): 72<br>Control: 42	BMI<br>Percentile	Short term (BMI only)<br>Medium term	High risk
Annesi 2017	5-11	High	Mixed	Diet and physical activity vs control	Intervention(s): 86<br>Control: 55	BMI	Short term<br>Medium term	High risk
Arlinghaus 2021	12-18	High	Low	Physical activity vs control	Intervention(s): 87<br>Control: 45	zBMI	Short term	High risk
Baranowski 2003	5-11	High	Mixed	Diet and physical activity vs control	Intervention(s): 19<br>Control: 16	BMI	Short term	Some concerns
Baranowski 2011	5-11	High	Mixed	Diet and physical activity vs control	Intervention(s): 103<br>Control: 50	zBMI<br>Percentile	Short term	High risk
Barbeau 2007	5-11	High	Mixed	Physical activity vs control	Intervention(s): 118<br>Control: 83	BMI	Medium term	High risk

Barnes 2015	5-11	High	Mixed	Physical activity vs control	Intervention(s): 25<br>Control: 23	zBMI<br>BMI	Short term	Some concerns
Barnes 2021	5-11	High	Low	Diet vs Physical activity vs Diet and physical activity vs control	Intervention(s): SWAP IT: 283; Physically Active children in Education (PACE): 163; SWAP IT + PACE combined: 202<br>Control: 167	zBMI<br>BMI	Medium term	Some concerns
Bayne-Smith 2004	12-18	High	Mixed	Diet and physical activity vs control	Intervention(s): 310<br>Control: 132	BMI	Short term	High risk
Beech 2003	5-11	High	Mixed	Diet and physical activity vs control	Intervention(s): 21<br>Control: 18	BMI	Short term	Some concerns
Black 2010	12-18	High	Low	Diet and physical activity vs control	Intervention(s): 121<br>Control: 114	zBMI	Medium term<br>Long term	Some concerns
Bogart 2016	12-18	High	Low	Diet and physical activity vs control	Intervention(s): 829<br>Control: 539	Percentile	Long term	High risk
Bohnert 2013	5-11	High	Low	Diet and physical activity vs control	Intervention(s): 52<br>Control: 24	zBMI	Short term	High risk
Bonsergent 2013	12-18	High	Mixed	Diet and physical activity (three groups) vs control	Intervention(s): Education: 1949; Environment: 1728; Screening and care: 1687<br>Control: 1589	zBMI<br>BMI	Long term	Some concerns
Brandstetter 2012	5-11	High	Mixed	Diet and physical activity vs control	Intervention(s): 450<br>Control: 495	BMI	Long term	Some concerns
Branscum 2013	5-11	High	Mixed	Diet and physical activity vs Diet and physical activity	Intervention(s): Comics for Health: 36; Knowledge based intervention: 34	Percentile	Short term	High risk
Breheny 2020	5-11	High	Low	Physical activity vs control	Intervention(s): 1153<br>Control: 1127	zBMI	Short term<br>Medium term	Some concerns
Brito Beck da Silva 2019	12-18	Non-High	Mixed	Diet and physical activity vs control	Intervention(s): 425<br>Control: 457	BMI	Medium term	Some concerns

Brown 2013	5-11	High	Low	Diet and physical activity vs control	Intervention(s): 31<br>Control: 32	zBMI<br>BMI<br>Percentile	Short term	Some concerns
Caballero 2003	5-11	High	Low	Diet and physical activity vs control	Intervention(s): 879<br>Control: 825	BMI	Long term	Some concerns
Cao 2015	5-11	Non-High	Mixed	Diet and physical activity vs control	Intervention(s): 965<br>Control: 889	zBMI	Medium term<br>Long term	Some concerns
Chai 2019	5-11	High	Mixed	Diet (two groups) vs control	Intervention(s): Telehealth: 16; Telehealth + SMS: 15<br>Control: 15	zBMI<br>BMI	Short term	High risk
Chen 2010	5-11	High	Mixed	Diet and physical activity vs control	Intervention(s): 35<br>Control: 32	BMI	Short term	High risk
Chen 2011	12-18	High	Mixed	Diet and physical activity vs control	Intervention(s): 27<br>Control: 27	BMI	Short term	Some concerns
Choo 2020	5-11	High	Mixed	Diet and physical activity vs control	Intervention(s): 49<br>Control: 55	zBMI	Short term	High risk
Clemes 2020	5-11	High	Low	Physical activity vs control	Intervention(s): 84<br>Control: 90	BMI	Short term	Low risk
Coleman 2012	5-11	High	Low	Diet vs control	Intervention(s): 279<br>Control: 300	Proportion	Medium term<br>Long term	High risk
Crespo 2012	5-11	High	Mixed	Diet and physical activity (three groups) vs control	Intervention(s): Family/Home + School/Community: 163; Family/Home: 195; School/Community: 216<br>Control: 223	zBMI<br>Percentile	Medium term<br>Long term	Some concerns
Cunha 2013*	5-11	Non-High	Low	Diet vs control	Intervention(s): 242<br>Control: 236	BMI	Medium term	Some concerns
Damsgaard 2014	5-11	High	Mixed	Diet vs control	Intervention(s): 412<br>Control: 411	zBMI	Short term	Some concerns
Davis 2021	5-11	High	Low	Diet vs control	Intervention(s): 1412<br>Control: 1723	zBMI<br>BMI<br>Percentile	Medium term	High risk

De Bock 2013	5-11	High	Mixed	Physical activity vs control	Intervention(s): 363<br>Control: 328	BMI	Short term<br>Medium term	High risk
de Greeff 2016	5-11	High	Mixed	Physical activity vs control	Intervention(s): 181<br>Control: 195	BMI	Short term	Some concerns
De Heer 2011	5-11	High	Low	Diet and physical activity vs control	Intervention(s): 242<br>Control: 326	BMI Percentile	Short term	Some concerns
de Ruyter 2012	5-11	High	Mixed	Diet vs control	Intervention(s): 319<br>Control: 322	zBMI	Short term<br>Medium term<br>Long term	Low risk (short term)<br>Some concerns (medium and long term)
Dewar 2013	12-18	High	Low	Diet and physical activity vs control	Intervention(s): 178<br>Control: 179	zBMI<br>BMI	Medium term<br>Long term	Some concerns
Diaz-Castro 2021	5-11	High	Mixed	Physical activity vs control	Intervention(s): 52<br>Control: 51	zBMI<br>BMI	Short term	Some concerns
Donnelly 2009	5-11	High	Mixed	Physical activity vs control	Intervention(s): 814<br>Control: 713	BMI	Long term	High risk
Drummy 2016	5-11	High	Mixed	Physical activity vs control	Intervention(s): 54<br>Control: 53	BMI	Short term	High risk
Duncan 2019	5-11	High	Mixed	Diet and physical activity vs control	Intervention(s): 346<br>Control: 329	BMI	Short term	Some concerns
Dunker 2018	12-18	Non-High	Mixed	Diet and physical activity vs control	Intervention(s): 131<br>Control: 139	BMI	Short term	Some concerns
Ebbeling 2006	12-18	High	Mixed	Diet vs control	Intervention(s): 53<br>Control: 50	BMI	Short term	Some concerns
El Ansari 2010	12-18	Non-High	Mixed	Physical activity vs control	Intervention(s): 80<br>Control: 80	BMI	Short term	Some concerns
Elder 2014	5-11	High	Mixed	Diet and physical activity vs control	Intervention(s): 271<br>Control: 267	zBMI<br>BMI<br>Percentile	Medium term<br>Long term	Some concerns
Ezendam 2012	12-18	High	Mixed	Diet and physical activity vs control	Intervention(s): 440<br>Control: 376	BMI	Long term	Some concerns

Fairclough 2013	5-11	High	Low	Diet and physical activity vs control	Intervention(s): 138<br>Control: 127	zBMI<br>BMI	Short term	High risk
Farmer 2017	5-11	High	Mixed	Physical activity vs control	Intervention(s): 374<br>Control: 369	zBMI<br>BMI	Medium term<br>Long term	Some concerns
Ford 2013	5-11	High	Mixed	Physical activity vs control	Intervention(s): 77<br>Control: 75	BMI	Short term	High risk
Foster 2008	5-11	High	Low	Diet and physical activity vs control	Intervention(s): 479<br>Control: 364	zBMI<br>BMI	Long term	High risk
French 2011	12-18	High	Mixed	Diet and physical activity vs control	Intervention(s): 38<br>Control: 38	zBMI	Medium term	Some concerns
Fulkerson 2010	5-11	High	Mixed	Diet vs control	Intervention(s): 22<br>Control: 22	zBMI<br>Percentile	Short term	Some concerns
Fulkerson 2015	5-11	High	Mixed	Diet vs control	Intervention(s): 74<br>Control: 75	zBMI	Medium term<br>Long term	Some concerns
Fulkerson 2022	5-11	High	Mixed	Diet and physical activity vs control	Intervention(s): 56<br>Control: 46	zBMI	Medium term	High risk
Gentile 2009	5-11	High	Mixed	Diet and physical activity vs control	Intervention(s): 642<br>Control: 640	BMI	Short term<br>Medium term	High risk
Greve 2015*	5-11	High	Mixed	Diet and physical activity vs control	Intervention(s): 6460<br>Control: 6459	BMI	Long term	High risk
Griffin 2019	5-11	High	Mixed	Diet and physical activity vs control	Intervention(s): 42<br>Control: 19	zBMI	Short term	High risk
Grydeland 2014	5-11	High	Mixed	Diet and physical activity vs control	Intervention(s): 465<br>Control: 859	zBMI<br>BMI	Long term	High risk
Gustafson 2019	12-18	High	Mixed	Diet vs control	Intervention(s): 277<br>Control: 134	Percentile	Short term	High risk
Ha 2021	5-11	Non-High	Mixed	Physical activity vs control	Intervention(s): 83<br>Control: 77	BMI	Short term<br>Medium term	Some concerns
Habib-Mourad 2014	5-11	Non-High	Mixed	Diet and physical activity vs control	Intervention(s): 193<br>Control: 181	BMI	Short term	Some concerns
Habib-Mourad 2020	5-11	Non-High	Mixed	Diet and physical activity vs control	Intervention(s): 698<br>Control: 541	zBMI	Long term	High risk

Haerens 2006	12-18	High	Mixed	Diet and physical activity vs control	Intervention(s): 1116<br>Control: 671	zBMI<br>BMI	Medium term<br>Long term	Some concerns (medium term)<br>High risk (long term)
Haire-Joshu 2010	5-11	High	Mixed	Diet and physical activity vs control	Intervention(s): 154<br>Control: 69	zBMI	Short term	High risk
Han 2006	5-11	Non-High	Mixed	Diet vs control	Intervention(s): 1333<br>Control: 1345	Proportion	Long term	High risk
Hannon 2018	5-11	High	Mixed	Diet and physical activity vs Diet and physical activity	Intervention(s):<br>Intervention 1: 72;<br>Intervention 2: 82	Percentile	Short term<br>Medium term	High risk (short term)<br>Some concerns (medium term)
Harrington 2018	12-18	High	Mixed	Physical activity vs control	Intervention(s): 867<br>Control: 885	zBMI	Short term<br>Medium term	Some concerns
HEALTHY Study Group 2010	5-11	High	Low	Diet and physical activity vs control	Intervention(s): 2307<br>Control: 2296	zBMI	Long term	Some concerns
Hendrie 2011	5-11	High	Mixed	Diet vs control	Intervention(s): 76<br>Control: 69	zBMI<br>BMI	Short term	Some concerns
Hendy 2011	5-11	High	Mixed	Diet and physical activity vs control	Intervention(s): 102<br>Control: 98	Percentile	Short term	Some concerns
Hollis 2016	12-18	High	Low	Physical activity vs control	Intervention(s): 645<br>Control: 505	zBMI<br>BMI	Medium term<br>Long term	Low risk
Hopper 2005	5-11	High	Mixed	Diet and physical activity vs control	Intervention(s): 142<br>Control: 96	BMI	Short term	High risk
Hovell 2018	12-18	High	Mixed	Diet and physical activity vs control	Intervention(s): 332<br>Control: 361	zBMI	Long term	High risk
Howe 2011	5-11	High	Mixed	Physical activity vs control	Intervention(s): 62<br>Control: 44	BMI	Medium term	Some concerns
Hull 2018	5-11	High	Mixed	Diet and physical activity vs control	Intervention(s): 162<br>Control: 157	zBMI<br>BMI	Short term<br>Long term	High risk

Ickovics 2019	5-11	High	Low	Diet vs Physical activity vs Diet and physical activity vs control	Intervention(s): Nutrition: 152; Physical activity: 178; Nutrition and physical activity: 152<br>Control: 113	Percentile	Long term	High risk
Isensee 2018	12-18	High	Mixed	Physical activity vs control	Intervention(s): 649<br>Control: 371	Percentile	Medium term	High risk
Jago 2006	12-18	High	Mixed	Physical activity vs Diet	Intervention(s): Physical activity: 227<br>Diet: 224	BMI<br>Percentile	Short term	High risk
James 2004	5-11	High	Mixed	Diet vs control	Intervention(s): 311<br>Control: 304	zBMI<br>BMI	Medium term<br>Long term	Some concerns
Jansen 2011	5-11	High	Low	Diet and physical activity vs control	Intervention(s): 1240<br>Control: 1382	BMI	Short term	High risk
Jones 2015	5-11	High	Mixed	Physical activity vs control	Intervention(s): 19<br>Control: 18	zBMI<br>BMI	Short term<br>Medium term	Some concerns
Kain 2014	5-11	High	Low	Diet and physical activity vs control	Intervention(s): 651<br>Control: 823	zBMI<br>BMI	Medium term	High risk
Keller 2009	5-11	High	Mixed	Diet and physical activity vs control	Intervention(s): 180<br>Control: 185	zBMI	Medium term	High risk
Kennedy 2018	12-18	High	Mixed	Physical activity vs control	Intervention(s): 348<br>Control: 252	zBMI<br>BMI	Short term<br>Medium term	Low risk
Keshani 2016	5-11	Non-High	Mixed	Diet vs control	Intervention(s): 83<br>Control: 88	BMI	Medium term	High risk
Ketelhut 2022	5-11	High	Low	Physical activity vs control	Intervention(s): 18<br>Control: 16	BMI	Short term	High risk
Khan 2014	5-11	High	Mixed	Physical activity vs control	Intervention(s): 64<br>Control: 68	zBMI<br>BMI	Medium term	High risk
Kipping 2008*	5-11	High	Mixed	Diet and physical activity vs control	Intervention(s): 249<br>Control: 223	BMI	Short term	High risk
Kipping 2014	5-11	High	Mixed	Diet and physical activity vs control	Intervention(s): 889<br>Control: 953	zBMI	Short term<br>Long term	Some concerns

Klesges 2010	5-11	High	Mixed	Diet and physical activity vs control	Intervention(s): 153<br>Control: 150	BMI	Medium term<br>Long term	Some concerns
Kobel 2017	5-11	High	Mixed	Diet and physical activity vs control	Intervention(s): 307<br>Control: 200	BMI<br>Percentile	Medium term	High risk
Kocken 2016	5-11	High	Mixed	Diet and physical activity vs control	Intervention(s): 615<br>Control: 497	zBMI	Short term<br>Long term	High risk
Kovalskys 2016	5-11	Non-High	Mixed	Physical activity vs control	Intervention(s): 424<br>Control: 336	zBMI	Long term	High risk
Kriemler 2010	5-11	High	Mixed	Physical activity vs control	Intervention(s): 297<br>Control: 205	BMI	Medium term<br>Long term	Low risk (medium term)<br>High risk (long term)
Kubik 2021	5-11	High	Mixed	Diet and physical activity vs control	Intervention(s): 66<br>Control: 66	zBMI<br>BMI	Medium term<br>Long term	Some concerns
Kuhlemeier 2022	12-18	High	Low	Diet and physical activity vs control	Intervention(s): 318<br>Control: 290	zBMI	Long term	High risk
Kuroko 2020	12-18	High	Mixed	Diet vs control	Intervention(s): 88<br>Control: 27	zBMI	Medium term	Some concerns
Lappe 2017**	12-18	High	Mixed	Diet vs control	Intervention(s): 136<br>Control: 138	Percentile	Medium term	Some concerns
Lau 2016	5-11	Non-High	Mixed	Physical activity vs control	Intervention(s): 40<br>Control: 40	BMI	Short term	Some concerns
Lazaar 2007	5-11	High	Mixed	Physical activity vs control	Intervention(s): 138<br>Control: 187	zBMI<br>BMI	Short term	Some concerns
Leme 2018	12-18	Non-High	Low	Diet and physical activity vs control	Intervention(s): 142<br>Control: 111	zBMI<br>BMI	Short term<br>Medium term	Some concerns
Lent 2014	5-11	High	Low	Diet vs control	Intervention(s): 435<br>Control: 332	zBMI<br>BMI<br>Percentile	Medium term<br>Long term	Some concerns (medium term)<br>High risk (long term)
Levy 2012	5-11	Non-High	Mixed	Diet and physical activity vs control	Intervention(s): 510<br>Control: 509	Proportion	Short term	Some concerns

Li 2010	5-11	Non-High	Mixed	Physical activity vs control	Intervention(s): 2329<br>Control: 2371	zBMI<br>BMI	Medium term<br>Long term	Some concerns
Li 2019	5-11	Non-High	Mixed	Diet and physical activity vs control	Intervention(s): 832<br>Control: 809	zBMI	Medium term	Low risk
Lichtenstein 2011	5-11	High	Mixed	Diet and physical activity vs control	Intervention(s): 249<br>Control: 196	zBMI	Medium term<br>Long term	High risk
Liu 2019	5-11	Non-High	Mixed	Diet and physical activity vs control	Intervention(s): 930<br>Control: 959	zBMI<br>BMI	Short term<br>Medium term	Some concerns
Liu 2022	5-11	Non-High	Mixed	Diet and physical activity vs control	Intervention(s): 703<br>Control: 670	zBMI<br>BMI	Short term<br>Medium term	Low risk
Llargues 2012	5-11	High	Mixed	Diet and physical activity vs control	Intervention(s): 225<br>Control: 201	BMI	Long term	Some concerns
Lloyd 2018	5-11	High	Mixed	Diet and physical activity vs control	Intervention(s): 676<br>Control: 648	zBMI<br>BMI	Long term	Low risk
Lubans 2021	12-18	High	Mixed	Physical activity vs control	Intervention(s): 333<br>Control: 328	zBMI	Short term<br>Medium term	Some concerns (short term)<br>High risk (medium term)
Luszczynska 2016b	12-18	High	Mixed	Diet (two groups) vs control	Intervention(s): Planning: 153; Self-efficacy: 172<br>Control: 181	BMI	Medium term	High risk
Magnusson 2012	5-11	High	Mixed	Diet and physical activity vs control	Intervention(s): 103<br>Control: 82	BMI	Long term	High risk
Marcus 2009	5-11	High	Mixed	Diet and physical activity vs control	Intervention(s): 1538<br>Control: 1300	zBMI	Long term	High risk
Martinez-Vizcaino 2014	5-11	High	Mixed	Physical activity vs control	Intervention(s): 229<br>Control: 240	BMI	Medium term	Some concerns
Martinez-Vizcaino 2020	5-11	High	Mixed	Physical activity vs control	Intervention(s): 619<br>Control: 815	zBMI<br>BMI	Short term	High risk
Martinez-Vizcaino 2022	5-11	High	Mixed	Physical activity vs control	Intervention(s): 248<br>Control: 239	zBMI<br>BMI	Medium term	High risk
Melnyk 2013	12-18	High	Mixed	Physical activity vs control	Intervention(s): 358<br>Control: 421	BMI	Short term<br>Medium term	High risk

Meng 2013 (Beijing)	5-11	Non-High	Mixed	Diet vs Physical activity vs control	Intervention(s): Nutrition education: 615; Happy 10 intervention: 590<br>Control: 460	zBMI<br>BMI	Medium term	High risk
Mihas 2010	12-18	High	Mixed	Diet vs control	Intervention(s): 98<br>Control: 93	BMI	Medium term	Some concerns
Morgan 2011	5-11	High	Mixed	Diet and physical activity vs control	Intervention(s): 39<br>Control: 32	zBMI	Short term	High risk
Morgan 2014	5-11	High	Mixed	Diet and physical activity vs control	Intervention(s): 72<br>Control: 60	zBMI<br>BMI	Short term	Some concerns
Morgan 2019	5-11	High	Mixed	Physical activity vs control	Intervention(s): 74<br>Control: 79	zBMI	Medium term	Some concerns
Muller 2016	5-11	High	Mixed	Physical activity vs control	Intervention(s): 109<br>Control: 73	zBMI	Medium term	Some concerns
Muller 2019	5-11	Non-High	Mixed	Physical activity vs control	Intervention(s): 300<br>Control: 446	zBMI	Medium term	High risk
Muzaffar 2019	5-11	High	Mixed	Diet and physical activity vs Diet and physical activity	Intervention(s): PAWS Peer led: 49; PAWS Adult led: 52	Percentile	Short term<br>Medium term	High risk
NCT00224887 2005	5-11	High	Low	Diet vs control	Intervention(s): 154<br>Control: 153	BMI	Medium term	Some concerns
NCT02067728 2014	5-11; 12-18	High	Mixed	Diet and physical activity vs control	Intervention(s): 45<br>Control: 44	zBMI	Short term	High risk
Nemet 2011a	5-11	High	Low	Diet and physical activity vs control	Intervention(s): 68<br>Control: 62	BMI<br>Percentile	Medium term	Some concerns
Nemet 2011b	5-11	High	Low	Diet and physical activity vs control	Intervention(s): 118<br>Control: 85	BMI<br>Percentile	Medium term<br>Long term	Some concerns (medium term)<br>High risk (long term)
Neumark-Sztainer 2003	12-18	High	Mixed	Diet and physical activity vs control	Intervention(s): 89<br>Control: 112	BMI	Short term	High risk
Neumark-Sztainer 2010	12-18	High	Mixed	Diet and physical activity vs control	Intervention(s): 182<br>Control: 174	BMI	Short term<br>Medium term	Some concerns

Newton 2014	5-11	High	Mixed	Physical activity vs control	Intervention(s): 13<br>Control: 14	zBMI<br>BMI Percentile	Short term	Some concerns
Nicholl 2021	5-11	High	Mixed	Diet vs control	Intervention(s): 24<br>Control: 21	zBMI<br>BMI Percentile	Short term	Some concerns
Nollen 2014	5-11	High	Mixed	Diet and physical activity vs control	Intervention(s): 26<br>Control: 25	BMI	Short term	High risk
Nyberg 2015	5-11	High	Low	Diet and physical activity vs control	Intervention(s): 129<br>Control: 112	Proportion	Short term	Some concerns
Nyberg 2016	5-11	High	Low	Diet and physical activity vs control	Intervention(s): 185<br>Control: 193	zBMI	Short term<br>Medium term	Some concerns
O'Connor 2020	5-11	High	Mixed	Diet and physical activity vs control	Intervention(s): 31<br>Control: 33	zBMI	Short term	Some concerns
Ooi 2021	12-18	High	Low	Diet vs control	Intervention(s): 162<br>Control: 134	Proportion	Short term	High risk
Paineau 2008	5-11	High	Mixed	Diet (two groups) vs control	Intervention(s): Group A: 280; Group B: 275<br>Control: 394	zBMI<br>BMI	Short term	Some concerns
Papadaki 2010	12-18	High	Mixed	Diet (four groups) vs control	Intervention(s): LP/LGI: 101; LP)/HGI: 85; HP)/LGI: 91; HP)/HGI: 95<br>Control: 88	zBMI<br>BMI	Short term	High risk
Pate 2005	12-18	High	Mixed	Physical activity vs control	Intervention(s): 827<br>Control: 712	Proportion	Medium term	Some concerns
Patrick 2006	12-18	High	Mixed	Diet and physical activity vs control	Intervention(s): 1611<br>Control: 411	zBMI<br>BMI	Short term	High risk
Peralta 2009	12-18	High	Mixed	Diet and physical activity vs control	Intervention(s): 16<br>Control: 17	BMI	Short term	Some concerns
Pfeiffer 2019	12-18	High	Low	Physical activity vs control	Intervention(s): 753<br>Control: 766	zBMI	Short term	High risk
Prins 2012	12-18	High	Mixed	Physical activity (two groups) vs control	Intervention(s): YouRAction:118;	Proportion	Short term	Some concerns

					YouRAction+e: 136<br>Control: 132			
Puder 2011	5-11	High	Mixed	Diet and physical activity vs control	Intervention(s): 342<br>Control: 310	BMI	Medium term	Low risk
Ramirez-Rivera 2021	5-11	Non-High	Mixed	Diet and physical activity vs control	Intervention(s): 21<br>Control: 20	zBMI	Short term	Some concerns
Reesor 2019	12-18	High	Low	Diet and physical activity vs control	Intervention(s): 101<br>Control: 90	zBMI	Short term<br>Medium term	High risk
Rerksuppaphol 2017	5-11	Non-High	Mixed	Diet and physical activity vs control	Intervention(s): 111<br>Control: 106	zBMI<br>BMI	Short term	Some concerns
Rhodes 2019	5-11	High	Mixed	Physical activity vs control	Intervention(s): 52<br>Control: 50	BMI	Short term	Some concerns
Robinson 2003	5-11	High	Mixed	Physical activity vs Diet and physical activity	Intervention(s): Physical activity: 28; Diet and physical activity: 33	BMI	Short term	Some concerns
Robinson 2010	5-11	High	Mixed	Physical activity vs Diet and physical activity	Intervention(s): Physical activity: 134; Diet and physical activity: 127	zBMI<br>BMI	Long term	High risk
Rodearmel 2006	12-18	High	Mixed	Diet and physical activity vs control	Intervention(s): 52<br>Control: 19	Percentile	Short term	High risk
Rosario 2012	5-11	High	Mixed	Diet and physical activity vs control	Intervention(s): 231<br>Control: 233	zBMI<br>BMI	Short term	Some concerns
Rosenkranz 2010	5-11	High	Mixed	Diet and physical activity vs control	Intervention(s): 33<br>Control: 39	zBMI<br>BMI<br>Percentile	Short term	Some concerns
Rush 2012	5-11	High	Mixed	Diet and physical activity vs control	Intervention(s): 692<br>Control: 660	zBMI	Long term	High risk
Sacchetti 2013	5-11	High	Mixed	Physical activity vs control	Intervention(s): 212<br>Control: 216	BMI	Long term	Some concerns
Safdie 2013	5-11	Non-High	Low	Diet and physical activity (two groups) vs control	Intervention(s): Basic program: 252; Plus program: 224<br>Control: 354	BMI	Short term<br>Medium term<br>Long term	Some concerns

Sahota 2001	5-11	High	Mixed	Diet and physical activity vs control	Intervention(s): 314<br>Control: 322	zBMI	Medium term	Some concerns
Sahota 2019	5-11	High	Mixed	Diet and physical activity vs control	Intervention(s): 188<br>Control: 170	zBMI	Long term	Some concerns
Salmon 2022	5-11	High	Mixed	Physical activity (three groups) vs control	Intervention(s): Physical activity: 153; Sedentary behaviour: 120; Physical activity + Sedentary behaviour: 150<br>Control: 141	zBMI	Long term	Some concerns
Santos 2014	5-11	High	Mixed	Diet and physical activity vs control	Intervention(s): 340<br>Control: 307	zBMI	Medium term	Some concerns
Schreier 2013	12-18	High	Low	Diet and physical activity vs control	Intervention(s): 54<br>Control: 54	BMI	Short term	Some concerns
Seguin-Fawler 2021	5-11	High	Mixed	Diet vs control	Intervention(s): 148<br>Control: 157	Percentile	Short term	Some concerns
Sekhavat 2014	5-11	High	Mixed	Diet and physical activity vs control	Intervention(s): 87<br>Control: 81	zBMI<br>BMI	Medium term	Some concerns
Sgambato 2019	5-11	Non-High	Low	Diet and physical activity vs control	Intervention(s): 1290<br>Control: 1157	BMI	Short term	Some concerns
Sherwood 2019	5-11	High	Mixed	Diet and physical activity vs control	Intervention(s): 212<br>Control: 209	zBMI<br>Percentile	Medium term<br>Long term	Some concerns
Shin 2015	12-18	High	Low	Diet vs control	Intervention(s): 89<br>Control: 63	Percentile	Medium term	Some concerns
Shomaker 2019	12-18	High	Mixed	Diet vs control	Intervention(s): 29<br>Control: 25	zBMI<br>BMI<br>Percentile	Short term<br>Long term	Some concerns
Sichieri 2008	5-11	Non-High	Low	Diet vs control	Intervention(s): 526<br>Control: 608	BMI	Short term	Some concerns
Siegrist 2013	5-11	High	Mixed	Diet and physical activity vs control	Intervention(s): 422<br>Control: 297	zBMI<br>BMI	Medium term	Some concerns
Siegrist 2018	5-11	High	Mixed	Diet and physical activity vs control	Intervention(s): 243<br>Control: 191	BMI	Long term	Some concerns

Simon 2008	5-11	High	Mixed	Physical activity vs control	Intervention(s): 374<br>Control: 367	zBMI<br>BMI	Medium term (BMI only)<br>Long term	Some concerns
Simons 2015	12-18	High	Mixed	Physical activity vs control	Intervention(s): 134<br>Control: 126	zBMI	Short term<br>Medium term	Some concerns
Singh 2009	12-18	High	Mixed	Diet and physical activity vs control	Intervention(s): 601<br>Control: 452	BMI	Short term<br>Medium term<br>Long term	Some concerns
Smith 2014	12-18	High	Low	Physical activity vs control	Intervention(s): 181<br>Control: 180	BMI	Short term	Some concerns
Spiegel 2006	5-11	High	Mixed	Diet and physical activity vs control	Intervention(s): 534<br>Control: 479	Proportion	Short term	High risk
Stettler 2015	5-11	High	Mixed	Diet vs Diet and physical activity vs control	Intervention(s): Smart Steps - beverage-only: 76; Smart Steps - multiple behaviour: 63<br>Control: 33	zBMI<br>BMI	Medium term	Some concerns
Stolley 1997	5-11	High	Low	Diet and physical activity vs control	Intervention(s): 18<br>Control: 22	BMI	Short term<br>Medium term	High risk
Story 2003	5-11	High	Low	Diet and physical activity vs control	Intervention(s): 26<br>Control: 28	BMI	Short term	Some concerns
Story 2012	5-11	High	Low	Diet and physical activity vs control	Intervention(s): 267<br>Control: 187	zBMI<br>BMI	Long term	High risk
Takacs 2020	12-18	High	Mixed	Diet vs control	Intervention(s): 114<br>Control: 105	BMI	Medium term	Some concerns
Tanskey 2017	5-11	High	Low	Physical activity (two groups) vs control	Intervention(s): 100 Miles club: 261; Just Move: 249<br>Control: 259	zBMI<br>BMI	Medium term	High risk
Telford 2012	5-11	High	Mixed	Physical activity vs control	Intervention(s): 312<br>Control: 308	BMI	Long term	High risk

Tessier 2008	5-11	High	Mixed	Physical activity vs Physical activity	Intervention(s): REGU'LAPS: 474; Active control: 465	BMI	Short term	High risk
Thivel 2011	5-11	High	Mixed	Physical activity vs control	Intervention(s): 168<br>Control: 187	BMI	Short term	High risk
Topham 2021	5-11	High	Mixed	Diet and physical activity (four groups) vs control	Intervention(s): Family Lifestyle: 117; Family Lifestyle + Family Dynamics: 87; Family Dynamic + Peer Group: 124; Family Lifestyle + Family Dynamic + Peer Group: 129<br>Control: 81	zBMI	Long term	High risk
van de Berg 2020	5-11	High	Low	Diet vs Physical activity vs Diet and physical activity vs control	Intervention(s): Walk Across Texas (WAT!): 336; Learn!Grow! Eat!Go! (LGEG!): 347; Walk Across Texas (WAT!) + Learn!Grow! Eat!Go! (LGEG!): 358<br>Control: 285	Percentile	Medium term	High risk
Velez 2010	12-18	High	Mixed	Physical activity vs control	Intervention(s): 13<br>Control: 15	BMI	Short term	Some concerns
Viggiano 2015	12-18	High	Mixed	Diet vs control	Intervention(s): 1663<br>Control: 1447	zBMI	Short term<br>Long term	Some concerns
Viggiano 2018	5-11	High	Mixed	Diet vs control	Intervention(s): 837<br>Control: 476	zBMI	Short term<br>Long term	High risk
Vizcaino 2008	5-11	High	Mixed	Physical activity vs control	Intervention(s): 513<br>Control: 606	BMI	Medium term	Some concerns
Wang 2012	5-11	Non-High	Mixed	Diet and physical activity vs control	Intervention(s): 476<br>Control: 527	Proportion	Medium term	High risk

Wang 2018	5-11	Non-High	Mixed	Physical activity vs control	Intervention(s): 5275<br>Control: 4583	zBMI<br>BMI	Medium term	Some concerns
Weeks 2012	12-18	High	Mixed	Physical activity vs control	Intervention(s): 52<br>Control: 47	BMI	Short term	Some concerns
Wendel 2016	5-11	High	Mixed	Physical activity vs control	Intervention(s): 121<br>Control: 72	BMI Percentile	Long term	High risk
White 2019	5-11	High	Low	Diet and physical activity vs control	Intervention(s): 151<br>Control: 77	zBMI	Short term<br>Medium term<br>Long term	High risk
Whittemore 2013	12-18	High	Mixed	Diet and physical activity vs Diet and physical activity	Intervention(s): HEALTH[e]TEEN+CST: 204; HEALTH[e]TEEN: 174	BMI	Short term	Some concerns
Wieland 2018	12-18	High	Low	Diet and physical activity vs control	Intervention(s): 40<br>Control: 41	BMI	Short term<br>Medium term	Some concerns
Wilksch 2015	12-18	High	Mixed	Diet and physical activity vs control	Intervention(s): 347<br>Control: 473	BMI	Short term<br>Medium term	Some concerns
Williamson 2012	5-11	High	Low	Diet and physical activity vs control	Intervention(s): 713<br>Control: 587	zBMI	Long term	Some concerns
Xu 2015	5-11	Non-High	Mixed	Diet and physical activity vs control	Intervention(s): 612<br>Control: 513	zBMI<br>BMI		Some concerns
Xu 2017 (5 other cities)	5-11	Non-High	Mixed	Diet and physical activity vs control	Intervention(s): 2656<br>Control: 2627	zBMI<br>BMI	Medium term	Some concerns
Yin 2012	5-11	High	Mixed	Physical activity vs control	Intervention(s): 292<br>Control: 284	zBMI	Medium term<br>Long term	Some concerns (medium term)<br>High risk (long term)

H Selecting indicator variables

The correlation matrix between the intervention indicators is shown in **Figure S*1***. We observed two absolute correlations ≥ 0.5 between (i) the total duration of the intervention and the duration of the peak engagement period (correlation of 0.66), and (ii) school setting and community setting (correlation of -0.72). Based on (i) we chose to remove peak duration from our set of indicators. Correlation (ii) reflects the fact that interventions tended to be based in either a school or a community environment, while home elements were often in supplement to one of these settings. Since the effect of all settings were deemed of interest by our stakeholders, we did not remove either of these variables.

Figure S*2* shows the proportion of interventions that were coded as 1 (rather than 0) for each intervention indicator variable. We observed six indicators that yielded identical responses more than 80% of the time: delivered in the home (81.6% No), targets activity (81.2% Yes), applied continuously (96.5% Yes), delivered to the child as part of a group (82.7% Yes), involved a single phase (81.2% Yes), commercial interests involved (89.4% No). Based on these results, we chose to remove the continuous, single phase and commercial interests indicators due to a lack of information. Next, we re-defined the home indicator to also include `home activity'. This meant that home was coded as 1 if the intervention was delivered in the home OR included some home based activity for the child. We also re-coded the group and individual indicators as one variable coded as 1 if the intervention was delivered to the child individually (either exclusively or in combination with group delivery) and 0 if there was no individual element. Finally, we chose to combine the flexibility and choice indicators which, while not quite reaching our 80% threshold, were both mostly coded as 0 (No) and represent a similar concept (i.e. the adaptability of the intervention to the preferences of the recipient or deliverer of the intervention). Since evaluating the effect of activity based interventions is of particular interest, we did not remove this indicator.

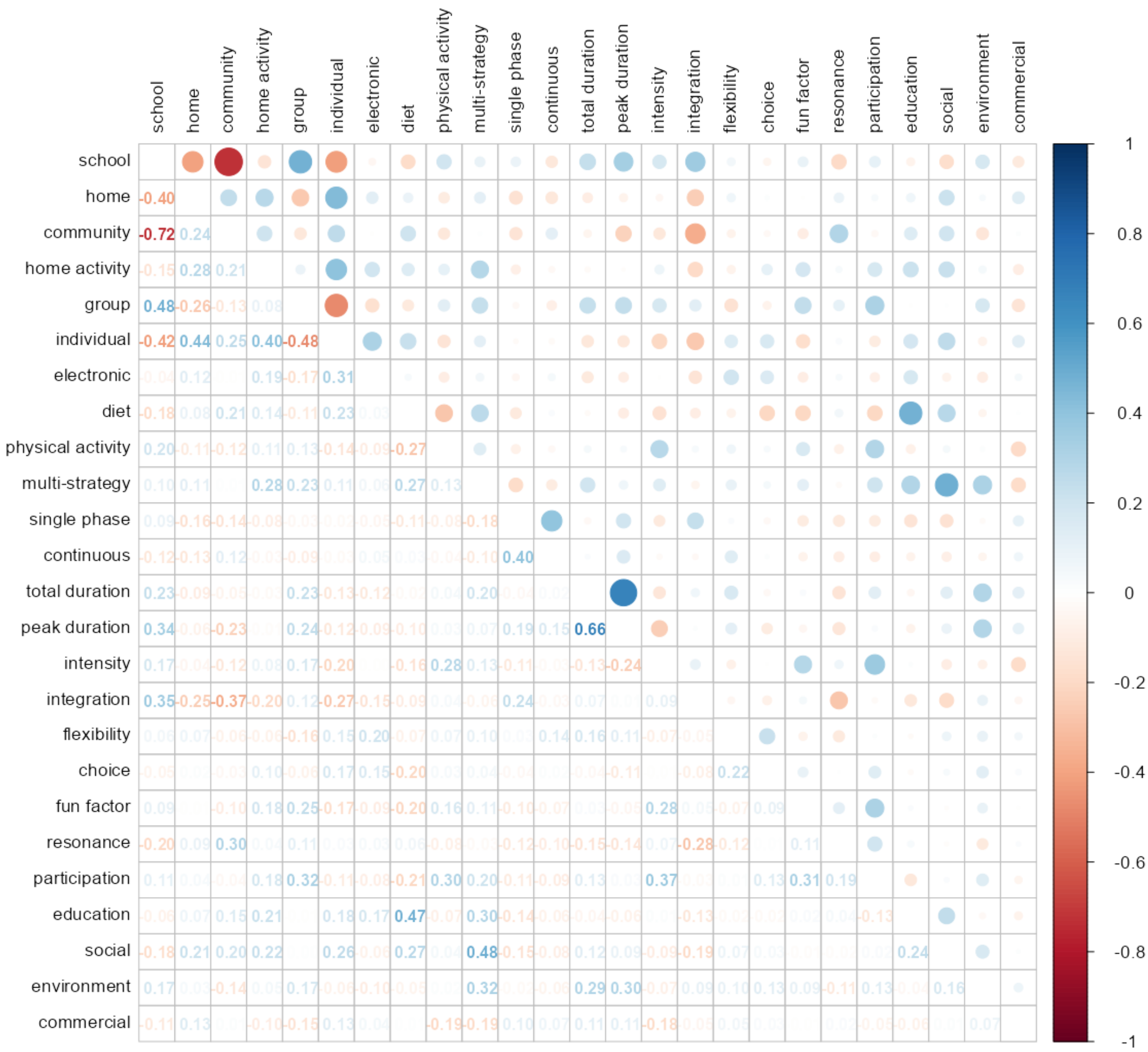

Figure S1: Correlation matrix describing the collinearity between pairs of intervention indicators. The colour and size of circles in the upper half of the matrix indicates the correlation between those variables. Negative correlations are indicated in red and positive correlations in blue (as shown in the key on the right hand side). The value of the correlation is shown in the equivalent box in the lower half of the matrix. The lowest correlations are very faint and sometimes cannot be seen.

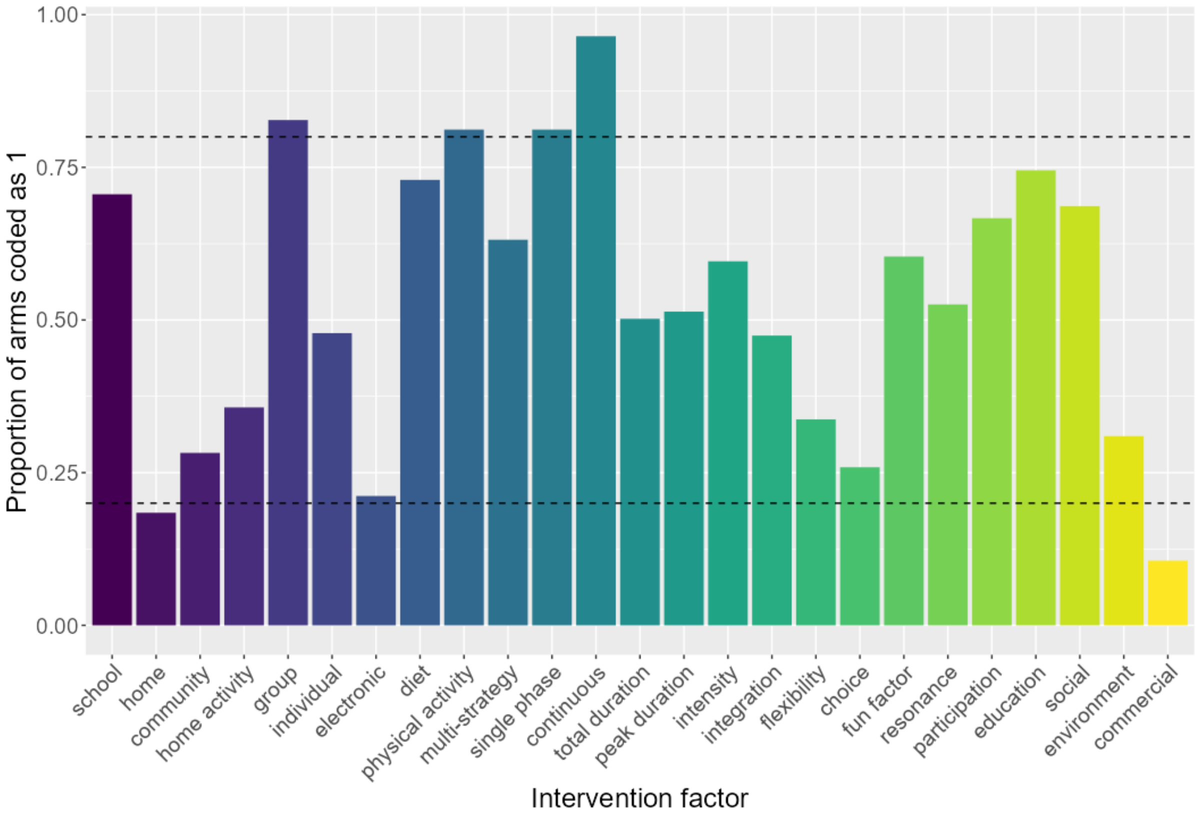

Figure S2: Bar chart showing the proportion of identical responses for each intervention indicator. Each bar represents the proportion of intervention arms coded as 1 in the data set (see Section A for a description of each indicator and its coding). The horizontal dotted lines are at 0.2 and 0.8 representing our preliminary cut off of 80% identical responses (for 0 and 1 respectively).

I Selecting interactions via SSVS

The results of the SSVS models at each step are shown in Sections I.1 (**Figure S*3***, **Figure S*4***, and **Figure S*5***) and I.2 (**Figure S*6***, **Figure S*7***, and **Figure S*8***) for the primary (random effects) and secondary (fixed effects) analyses respectively. **Table S*2*** compares the fit of the different models using the deviance information criterion (DIC) [9]. For both the RE and FE procedures, the final model with selected interactions has the lowest DIC indicating the best fit. The RE models have lower DIC (better fit) than the FE models.

Table S2. Deviance information criterion (DIC) for the models fitted at each step of the SSVS procedure (for both random effects and fixed effects).

		DIC	
**Model**	**Interactions**	**RE**	**FE**
SSVS step 1	No interactions	-560.9	-184.7
SSVS step 2	Interactions with age and all other indicators	-565.8	-217.9
SSVS step 3	Interactions with behaviour targeted and all other indicators	-560.8	-265.5
Primary	Selected interactions	-579.3	-305.3

I.1 Primary analysis (random effects)

I.1.1 Step 1: No interactions

Indicator variable	Estimate [95% CI]	P(<0)	P(>0)
Setting: school	-0.007 [-0.062, 0.047]	0.61	0.39
home	0.025 [-0.012, 0.063]	0.09	0.91
community	0.006 [-0.042, 0.056]	0.4	0.6
Mode: individually targeted	-0.007 [-0.045, 0.032]	0.63	0.37
electronic	0.019 [-0.022, 0.062]	0.18	0.82
Behaviour: physical activity added to diet	0.024 [-0.019, 0.066]	0.13	0.87
physical activity rather than diet	0.032 [-0.018, 0.081]	0.1	0.9
Multidimensional strategy	-0.025 [-0.067, 0.016]	0.89	0.11
Long duration	0.014 [-0.022, 0.051]	0.23	0.77
High intensity	-0.025 [-0.060, 0.010]	0.92	0.08
Integrated into the curriculum	0.001 [-0.033, 0.034]	0.48	0.52
Flexible intervention	-0.003 [-0.035, 0.030]	0.56	0.44
Fun factor	0.011 [-0.023, 0.045]	0.26	0.74
Resonance (delivered by someone external)	0.019 [-0.015, 0.052]	0.14	0.86
Mechanism: participation	0.003 [-0.036, 0.042]	0.44	0.56
education	0.028 [-0.010, 0.067]	0.07	0.93
change social environment	0.018 [-0.024, 0.060]	0.2	0.8
change physical environment	0.051 [0.017, 0.085]	0	1
Trial in: older age group	0.002 [-0.036, 0.040]	0.45	0.55
high-income country	0.030 [-0.020, 0.078]	0.12	0.88
mixed SES	-0.017 [-0.055, 0.020]	0.82	0.18
Follow-up: medium rather than short term	-0.030 [-0.060, -0.001]	0.98	0.02
long rather than short term	-0.017 [-0.051, 0.018]	0.83	0.17
High risk of bias	-0.005 [-0.036, 0.026]	0.63	0.37

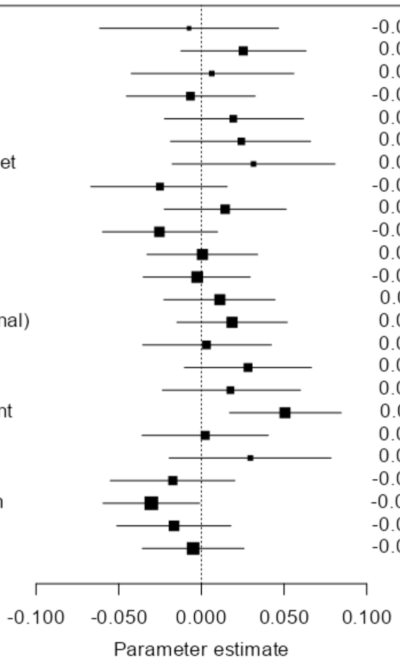

Figure S3: Parameter estimates from step 1 of the SSVS procedure (model with no interactions) for the primary (random effects) model. We list the probability that each coefficient is less than or greater than zero, $P(<0)$ and $P(>0)$. The estimates of the intercept and heterogeneity parameter are $\alpha\ [95\%\ CI] = -0.039[-0.054, -0.023]$ and $\tau\ [95\%\ CI] = 0.085\ [0.072, 0.099]$ respectively.

I.1.2 Step 2: Interactions with age

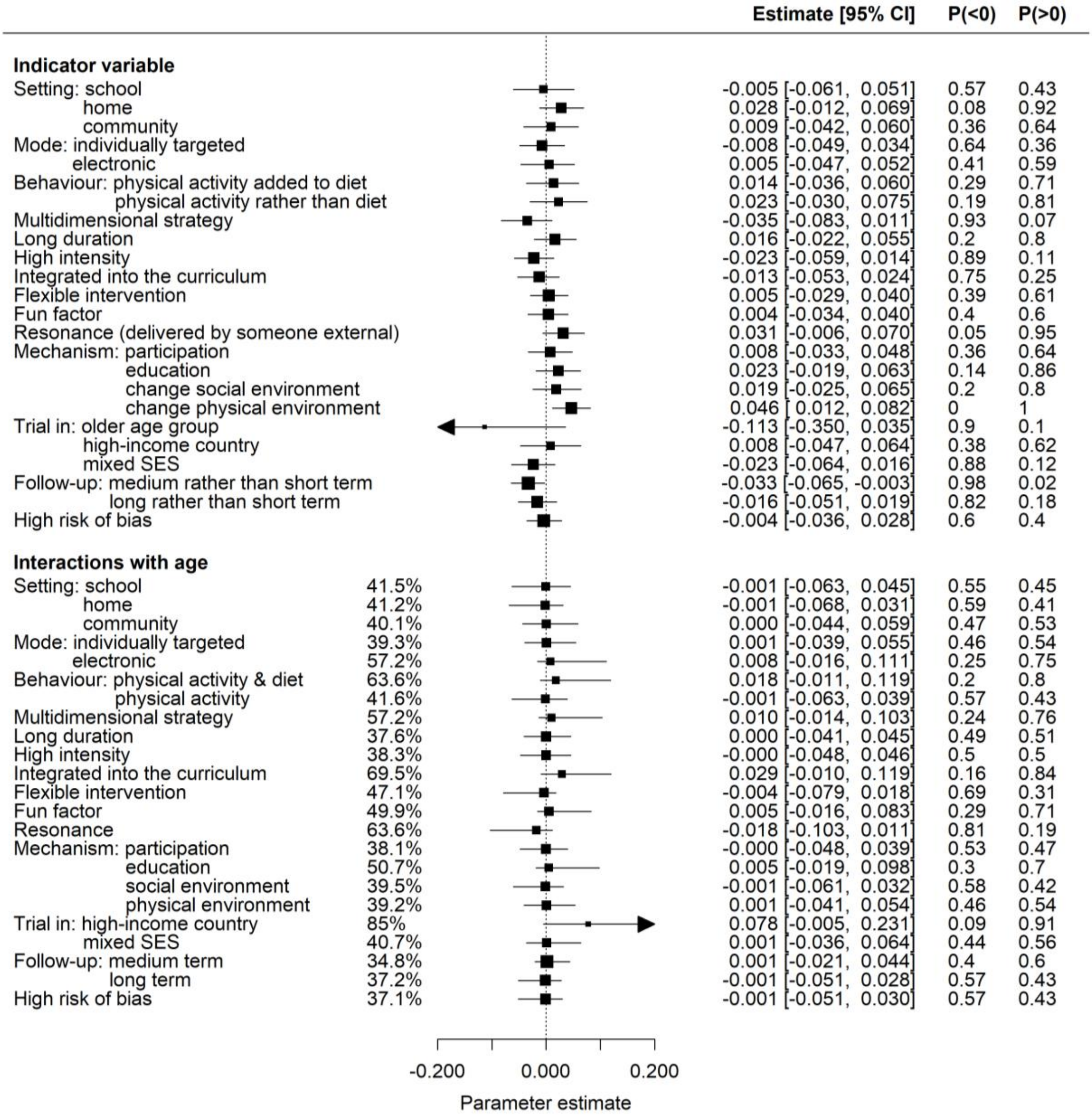

Figure S4: Parameter estimates from step 2 of the SSVS procedure (interactions between age and all other indicators) for the primary (random effects) model. We list the probability that each coefficient is less than or greater than zero, $P(< 0)$ and $P(> 0)$. For each interaction term we list the percentage of times it was selected by the SSVS model. The estimates of the intercept and heterogeneity parameter are $\alpha\ [95\%\ CI] = -0.038\ [-0.053, -0.023]$ and $\tau\ [95\%\ CI] = 0.082\ [0.070, 0.097]$ respectively.

I.1.3 Step 3: Interactions with change in behaviour targeted

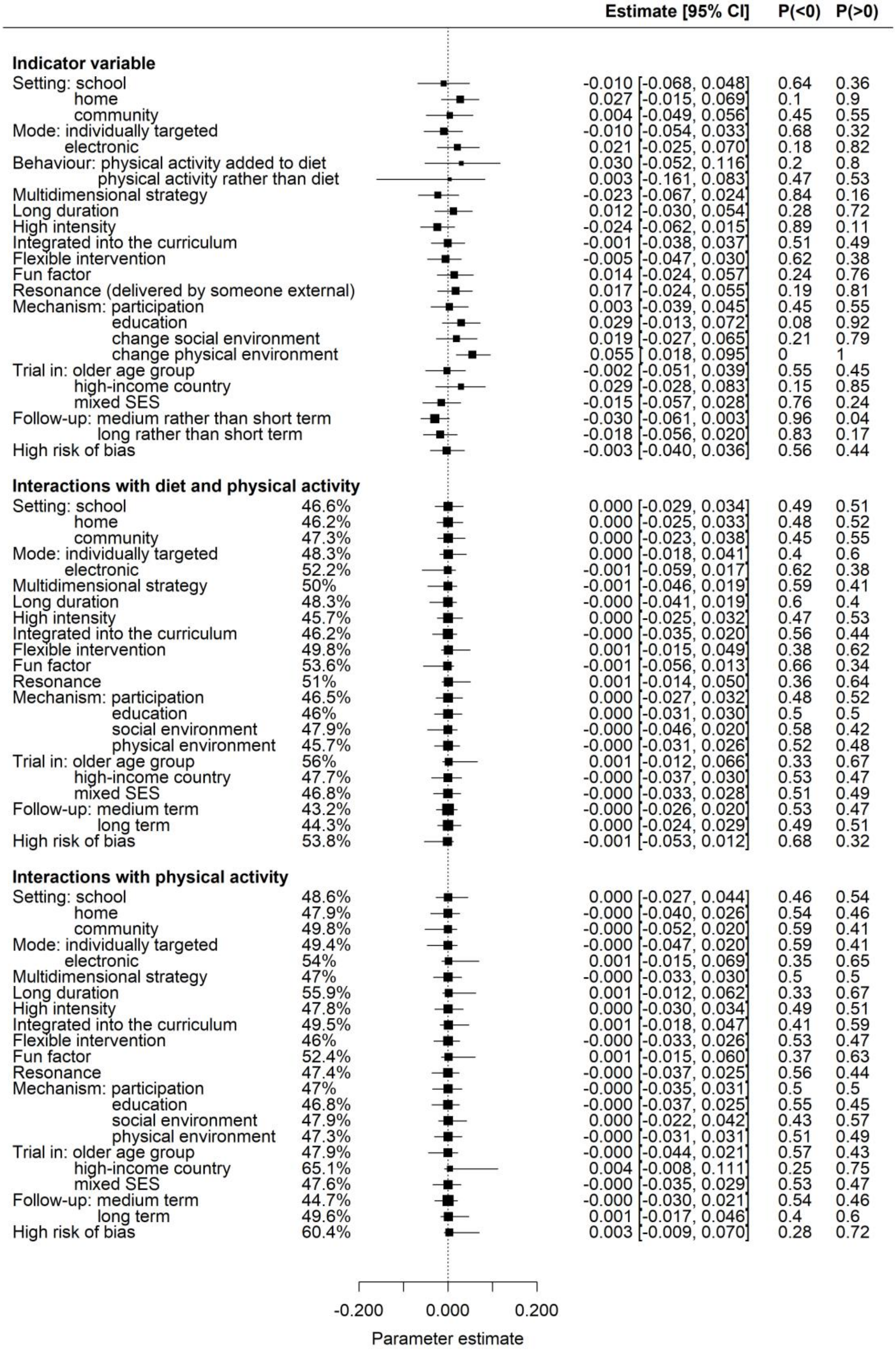

Figure S5: Parameter estimates from step 3 of the SSVS procedure (interactions between behaviour targeted (diet and/or activity) and all other indicators) for the primary (random effects) model. We list the probability that each coefficient is less than or greater than zero, $P(<0)$ and $P(>0)$. For each interaction term we list the percentage of times it was selected by the SSVS model. The estimates of the intercept and heterogeneity parameter are $\alpha\ [95\%\ CI] = -0.039\ [-0.054, -0.023]$ and $\tau\ [95\%\ CI] = 0.084\ [0.071, 0.098]$ respectively.

I.2 Secondary analysis (fixed effects)

I.2.1 Step 1: No interactions

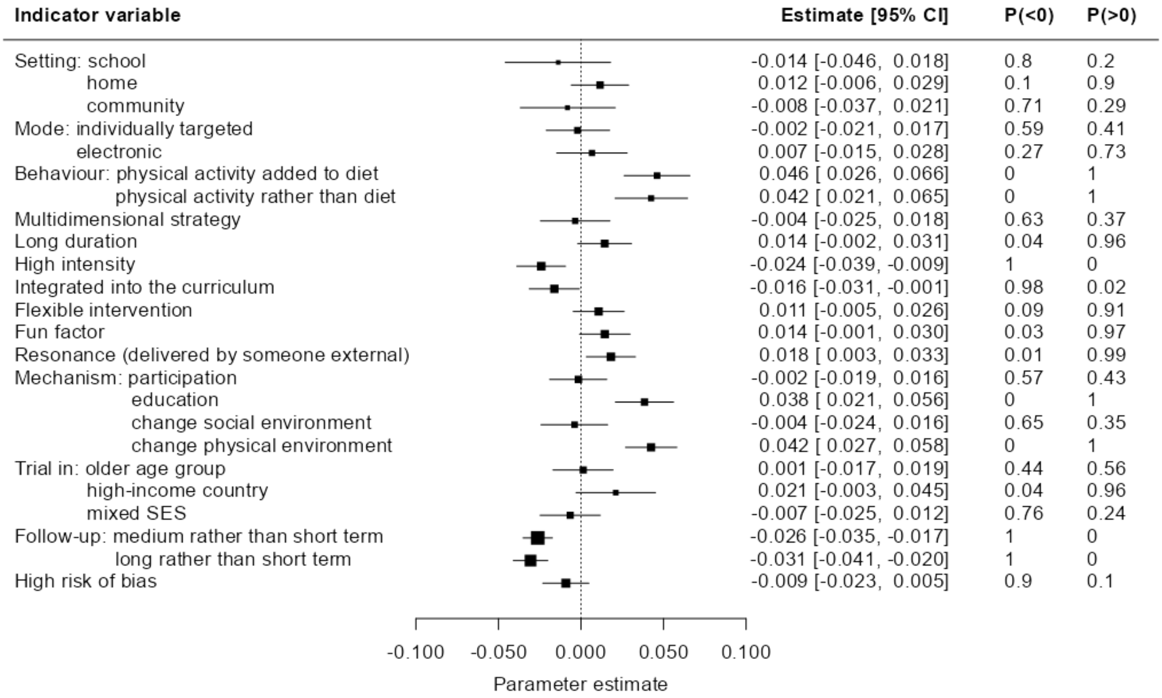

Figure S6: Parameter estimates from step 1 of the SSVS procedure (model with no interactions) for the secondary (fixed effects) model. We list the probability that each coefficient is less than or greater than zero, $P(< 0)$ and $P(> 0)$. The estimate of the intercept is α $[95\% \; CI] = -0.033[-0.041, -0.025]$.

I.2.2 Step 2: Interactions with age

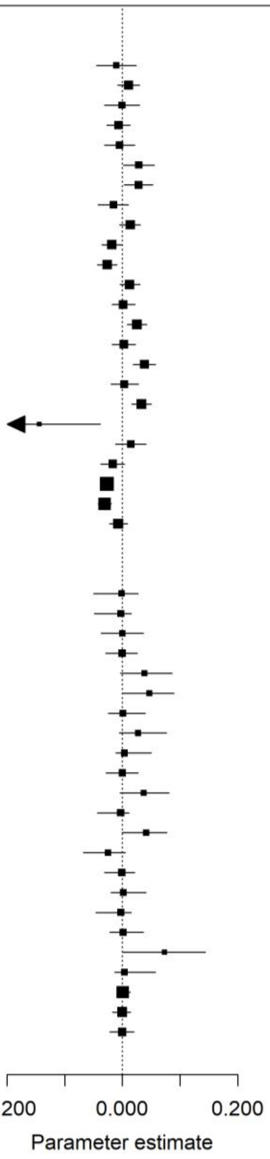

		Estimate [95% CI]	P(<0)	P(>0)
**Indicator variable**				
Setting: school		-0.010 [-0.045, 0.024]	0.72	0.28
home		0.011 [-0.008, 0.030]	0.13	0.87
community		-0.001 [-0.031, 0.030]	0.51	0.49
Mode: individually targeted		-0.007 [-0.027, 0.014]	0.74	0.26
electronic		-0.005 [-0.031, 0.021]	0.63	0.37
Behaviour: physical activity added to diet		0.028 [0.002, 0.055]	0.02	0.98
physical activity rather than diet		0.028 [0.003, 0.053]	0.02	0.98
Multidimensional strategy		-0.015 [-0.042, 0.010]	0.88	0.12
Long duration		0.014 [-0.005, 0.031]	0.07	0.93
High intensity		-0.018 [-0.036, -0.000]	0.98	0.02
Integrated into the curriculum		-0.026 [-0.044, -0.009]	1	0
Flexible intervention		0.013 [-0.004, 0.030]	0.07	0.93
Fun factor		0.001 [-0.018, 0.022]	0.44	0.56
Resonance (delivered by someone external)		0.025 [0.008, 0.042]	0	1
Mechanism: participation		0.002 [-0.018, 0.023]	0.41	0.59
education		0.038 [0.019, 0.058]	0	1
change social environment		0.004 [-0.020, 0.028]	0.39	0.61
change physical environment		0.033 [0.016, 0.050]	0	1
Trial in: older age group		-0.144 [-0.253, -0.039]	1	0
high-income country		0.015 [-0.012, 0.041]	0.14	0.86
mixed SES		-0.017 [-0.037, 0.004]	0.95	0.05
Follow-up: medium rather than short term		-0.027 [-0.037, -0.017]	1	0
long rather than short term		-0.031 [-0.042, -0.020]	1	0
High risk of bias		-0.007 [-0.022, 0.009]	0.81	0.19
**Interactions with age**				
Setting: school	38.6%	-0.001 [-0.050, 0.027]	0.58	0.42
home	39.4%	-0.003 [-0.049, 0.015]	0.66	0.34
community	36%	0.000 [-0.037, 0.036]	0.49	0.51
Mode: individually targeted	30.9%	-0.000 [-0.029, 0.026]	0.52	0.48
electronic	81.5%	0.038 [-0.003, 0.086]	0.07	0.93
Behaviour: physical activity & diet	91.7%	0.047 [-0.000, 0.090]	0.03	0.97
physical activity	34.4%	0.001 [-0.025, 0.039]	0.44	0.56
Multidimensional strategy	71%	0.027 [-0.006, 0.076]	0.11	0.89
Long duration	41.9%	0.004 [-0.011, 0.050]	0.29	0.71
High intensity	31.2%	-0.000 [-0.028, 0.027]	0.51	0.49
Integrated into the curriculum	79.8%	0.037 [-0.004, 0.081]	0.07	0.93
Flexible intervention	37.6%	-0.003 [-0.044, 0.011]	0.69	0.31
Fun factor	90.3%	0.041 [-0.000, 0.077]	0.03	0.97
Resonance	70.2%	-0.025 [-0.068, 0.005]	0.89	0.11
Mechanism: participation	31%	-0.001 [-0.031, 0.021]	0.56	0.44
education	34.9%	0.002 [-0.020, 0.041]	0.4	0.6
social environment	38.6%	-0.003 [-0.046, 0.015]	0.66	0.34
physical environment	33.1%	0.001 [-0.022, 0.036]	0.43	0.57
Trial in: high-income country	95.4%	0.073 [0.001, 0.143]	0.02	0.98
mixed SES	43.8%	0.004 [-0.013, 0.057]	0.3	0.7
Follow-up: medium term	19.1%	0.001 [-0.010, 0.013]	0.44	0.56
long term	22.5%	-0.000 [-0.017, 0.014]	0.53	0.47
High risk of bias	27.1%	-0.000 [-0.022, 0.020]	0.51	0.49

Figure S7: Parameter estimates from step 2 of the SSVS procedure (interactions between age and all other indicators) for the secondary (fixed effects) model. For each interaction term we list the percentage of times it was selected by the SSVS model. We list the probability that each coefficient is less than or greater than zero, $P(< 0)$ and $P(> 0)$. The estimate of the intercept is α $[95\%\ CI] = -0.034$ $[-0.042, -0.026]$.

I.2.3 Step 3: interactions with change in behaviour targeted

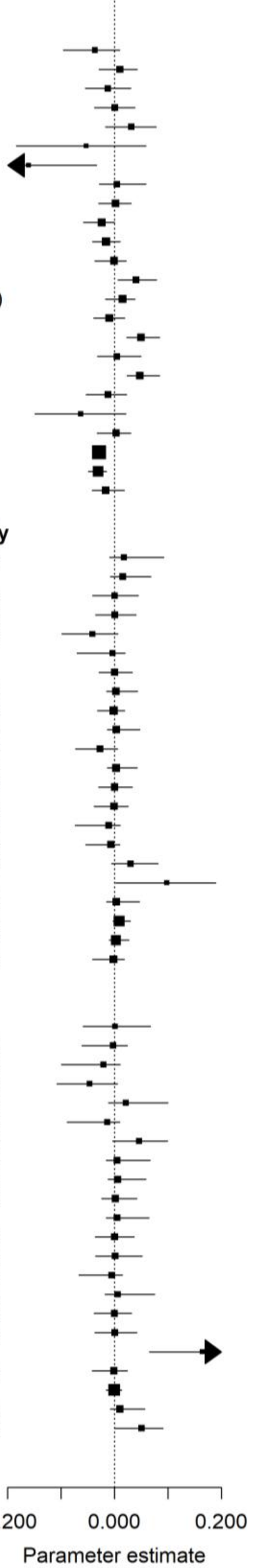

	% selected	Estimate [95% CI]	P(<0)	P(>0)
**Indicator variable**				
Setting: school		-0.037 [-0.096, 0.010]	0.94	0.06
home		0.010 [-0.030, 0.042]	0.31	0.69
community		-0.013 [-0.055, 0.030]	0.74	0.26
Mode: individually targeted		0.001 [-0.038, 0.038]	0.48	0.52
electronic		0.031 [-0.017, 0.078]	0.13	0.87
Behaviour: physical activity added to diet		-0.053 [-0.184, 0.058]	0.82	0.18
physical activity rather than diet		-0.161 [-0.310, -0.033]	0.99	0.01
Multidimensional strategy		0.005 [-0.028, 0.059]	0.4	0.6
Long duration		0.002 [-0.030, 0.031]	0.45	0.55
High intensity		-0.024 [-0.059, -0.000]	0.98	0.02
Integrated into the curriculum		-0.016 [-0.041, 0.010]	0.9	0.1
Flexible intervention		-0.001 [-0.037, 0.022]	0.53	0.47
Fun factor		0.040 [0.006, 0.078]	0.01	0.99
Resonance (delivered by someone external)		0.015 [-0.017, 0.038]	0.14	0.86
Mechanism: participation		-0.010 [-0.039, 0.019]	0.77	0.23
education		0.049 [0.022, 0.084]	0	1
change social environment		0.004 [-0.033, 0.049]	0.42	0.58
change physical environment		0.047 [0.023, 0.084]	0	1
Trial in: older age group		-0.013 [-0.054, 0.023]	0.73	0.27
high-income country		-0.064 [-0.149, 0.021]	0.93	0.07
mixed SES		0.003 [-0.033, 0.030]	0.42	0.58
Follow-up: medium rather than short term		-0.029 [-0.041, -0.019]	1	0
long rather than short term		-0.031 [-0.049, -0.015]	1	0
High risk of bias		-0.017 [-0.042, 0.018]	0.87	0.13
**Interactions with diet and physical activity**				
Setting: school	59.4%	0.017 [-0.010, 0.092]	0.17	0.83
home	57%	0.015 [-0.008, 0.068]	0.15	0.85
community	32.9%	-0.000 [-0.042, 0.044]	0.51	0.49
Mode: individually targeted	31.5%	0.001 [-0.036, 0.040]	0.47	0.53
electronic	75.6%	-0.041 [-0.099, 0.006]	0.91	0.09
Multidimensional strategy	40%	-0.004 [-0.071, 0.020]	0.66	0.34
Long duration	29%	0.000 [-0.030, 0.033]	0.5	0.5
High intensity	30.4%	0.003 [-0.015, 0.043]	0.36	0.64
Integrated into the curriculum	26.5%	-0.002 [-0.032, 0.019]	0.6	0.4
Flexible intervention	31.6%	0.003 [-0.014, 0.047]	0.34	0.66
Fun factor	68.1%	-0.027 [-0.073, 0.006]	0.9	0.1
Resonance	30.3%	0.003 [-0.014, 0.042]	0.34	0.66
Mechanism: participation	28.2%	0.000 [-0.030, 0.033]	0.5	0.5
education	28.6%	-0.001 [-0.039, 0.025]	0.55	0.45
social environment	52.9%	-0.011 [-0.074, 0.011]	0.8	0.2
physical environment	42.2%	-0.007 [-0.054, 0.009]	0.78	0.22
Trial in: older age group	68.3%	0.030 [-0.006, 0.081]	0.11	0.89
high-income country	96%	0.098 [0.002, 0.189]	0.02	0.98
mixed SES	32.3%	0.003 [-0.016, 0.047]	0.34	0.66
Follow-up: medium term	36.1%	0.008 [-0.003, 0.030]	0.09	0.91
long term	22.6%	0.003 [-0.010, 0.027]	0.34	0.66
High risk of bias	30.2%	-0.002 [-0.041, 0.018]	0.61	0.39
**Interactions with physical activity**				
Setting: school	41%	0.001 [-0.059, 0.067]	0.46	0.54
home	36.7%	-0.003 [-0.061, 0.023]	0.63	0.37
community	61.9%	-0.021 [-0.100, 0.011]	0.83	0.17
Mode: individually targeted	79.3%	-0.047 [-0.108, 0.005]	0.93	0.07
electronic	61.8%	0.021 [-0.012, 0.100]	0.18	0.82
Multidimensional strategy	56.3%	-0.014 [-0.089, 0.010]	0.82	0.18
Long duration	81.4%	0.046 [-0.004, 0.099]	0.06	0.94
High intensity	40.7%	0.005 [-0.016, 0.067]	0.3	0.7
Integrated into the curriculum	40.9%	0.006 [-0.013, 0.059]	0.26	0.74
Flexible intervention	29.9%	0.001 [-0.024, 0.041]	0.44	0.56
Fun factor	41%	0.005 [-0.016, 0.064]	0.31	0.69
Resonance	31.1%	-0.000 [-0.037, 0.036]	0.5	0.5
Mechanism: participation	34.4%	0.001 [-0.036, 0.051]	0.45	0.55
education	42.1%	-0.005 [-0.067, 0.015]	0.71	0.29
social environment	44.8%	0.005 [-0.018, 0.075]	0.29	0.71
physical environment	30.1%	-0.000 [-0.039, 0.031]	0.52	0.48
Trial in: older age group	32.3%	0.000 [-0.037, 0.041]	0.49	0.51
high-income country	100%	0.164 [0.065, 0.276]	0	1
mixed SES	30.6%	-0.002 [-0.042, 0.024]	0.58	0.42
Follow-up: medium term	18%	-0.001 [-0.016, 0.013]	0.56	0.44
long term	48.8%	0.010 [-0.008, 0.056]	0.17	0.83
High risk of bias	90.6%	0.051 [0.000, 0.090]	0.02	0.98

Figure S8: Parameter estimates from step 3 of the SSVS procedure (interactions between behaviour targeted (diet and/or activity) and all other indicators) for the secondary (fixed effects) model. We list the probability that each coefficient is less than or greater than zero, $P(<0)$ and $P(>0)$. For each interaction term we list the percentage of times it was selected by the SSVS model. The estimate of the intercept is $\alpha\ [95\%\ CI] = -0.032\ [-0.041, -0.024]$.

J Comparing random and fixed effects models

Here we summarize the differences between the RE and FE analyses. We identify effects with strong evidence as those with probabilities in either direction ($\mathrm{P}(< 0)$ or $\mathrm{P}(> 0)$) that are greater than or equal to 0.95. For probabilities that exceed or equal 0.9, we label these effects as having reasonable evidence. Any effect which has strong evidence in the same direction in both analyses, we consider 'robust'. Any effect which has strong evidence in one analysis and reasonable evidence in the other, we consider 'almost robust'. Based on this procedure, we find robust indicator effects (**Table S*3***) for physical activity alone versus diet alone (beneficial), environmental components (less beneficial), older age group (beneficial), high-income countries (beneficial) and medium verses short term (beneficial). The beneficial effect of integration is almost robust. We find robust antagonistic interactions (**Table S4**) between (i) the older age group and diet and physical activity interventions, (ii) the older age group and high-income countries, (iii) activity only and long interventions, and (iv) activity only interventions and high-income countries. Finally, we find an almost robust antagonistic interaction between the older age group and integrated interventions.

Table S3: A comparison of the direction and strength of evidence for indicator coefficients in the primary (random effects) and secondary (fixed effects) analyses. A coefficient direction <0 indicates a beneficial effect of an indicator coded as 1 and a direction >0 indicates a less beneficial effect. We categorize evidence as 'strong' if the probability that the coefficient takes a value one side of zero is greater than or equal to 0.95 and 'reasonable' if the probability is greater than or equal to 0.9. We label a coefficient as 'robust' if both analyses provide strong evidence in the same direction and 'almost robust' if one analyses is strong and the other is reasonable (in the same direction).

	**Primary analysis (RE)**		**Secondary analysis (FE)**		**Robustness**
**Indicator**	**Direction**	**Strength**	**Direction**	**Strength**	
School	-	-	<0	Strong	
Home	>0	Strong	-	-	
Community	-	-	-	-	
Individual	-	-	-	-	
Electronic	-	-	-	-	
Diet & activity	-	-	<0	Strong	
Activity	<0	Strong	<0	Strong	Robust
Multi-strategy	<0	Reasonable	-	-	
Duration	-	-	-	-	
Intensity	-	-	<0	Strong	
Integration	<0	Reasonable	<0	Strong	Almost robust
Flexibility/choice	-	-	-	-	
Fun factor	-	-	>0	Strong	
Resonance	-	-	>0	Strong	
Participation	-	-	-	-	
Education	-	-	>0	Strong	
Social	-	-	-	-	
Environment	>0	Strong	>0	Strong	Robust
Age	<0	Strong	<0	Strong	Robust
Income country	<0	Strong	<0	Strong	Robust
SES	-	-	-	-	
Medium term	<0	Strong	<0	Strong	Robust
Long term	-	-	<0	Strong	
Risk of bias	-	-	<0	Strong	

Table S4: A comparison of the direction and strength of evidence for interaction coefficients in the primary (random effects) and secondary (fixed effects) analyses. A coefficient direction <0 indicates a synergistic interaction between the two *indicator*s and a direction >0 indicates an antagonistic interaction. We categorize evidence as 'strong' if the probability that the coefficient takes a value one side of zero is greater than or equal to 0.95 and 'reasonable' if the probability is greater than or equal to 0.9. We label a coefficient as 'robust' if both analyses provide strong evidence in the same direction and 'almost robust' if one analyses is strong and the other is reasonable (in the same direction).

	**Primary analysis (RE)**		**Secondary analysis (FE)**		**Robustness**
**Interaction**	**Direction**	**Strength**	**Direction**	**Strength**	
**Interactions with age**					
Electronic	-	-	-	-	
Diet & activity	>0	Strong	>0	Strong	Robust
Multi-strategy	-	-	-	-	
Integration	>0	Reasonable	>0	Strong	Almost robust
Fun factor	*Not selected*		-	-	
Resonance	-	-	<0	Reasonable	
Education	-	-	*Not selected*		
Income country	>0	Strong	>0	Strong	Robust
**Interactions with diet & activity**					
School	*Not selected*		>0	Strong	
Home	*Not selected*		>0	Reasonable	
Electronic	-	-	<0	Strong	
Multi-strategy	-	-	*Not selected*		
Fun factor	-	-	<0	Strong	
Resonance			*Not selected*		
Social	*Not selected*		-	-	
Income country	*Not selected*		>0	1	
Risk of bias	-	-	*Not selected*		
**Interactions with activity**					
Community	*Not selected*		<0	Strong	
Individual	*Not selected*		<0	Strong	
Electronic	-	-	>0	Reasonable	
Multi-strategy	*Not selected*		<0	Reasonable	
Duration	>0	Strong	>0	Strong	Robust
Fun factor	-	-	*Not selected*		
Income country	>0	Strong	>0	Strong	Robust
Risk of bias	-	-	>0	Strong	

K Combination of indicator values

The best (most beneficial) and worst (least beneficial) combination of indicator values are shown in **Table S5** and **Table S6** respectively.

Table S5. The indicator values associated with the minimum (most negative) mean difference predicted from our primary (random-effects) model. We show the most beneficial combination of indicator values for each of the two age groups (5-11 years and 12-18 years) and the two levels of country income status (high and non-high). We find the same combination regardless of whether we specify follow-up time (short, medium or long) or SES (mixed or low). The resulting predicted mean difference in each scenario is shown at the bottom of the table. For a given age group and income status, the largest beneficial effect is obtained for medium term follow-up and mixed SES (or universal interventions; highlighted in bold). Overall, the model predicts the greatest benefit for the older age group in non-high-income countries (indicated with an asterisk).

		**12-18 years**		**5-11 years**	
**Indicator**		**Non-high income**	**High income**	**Non-high income**	**High income**
School		Yes	Yes	Yes	Yes
Home		No	No	No	No
Community		No	No	No	No
Individual		Yes	Yes	Yes	Yes
Electronic		No	No	No	Yes
Diet and physical activity		No	No	No	Yes
Physical activity		Yes	Yes	Yes	No
Multi-strategy		Yes	Yes	Yes	Yes
Duration		Short	Short	Short	Short
Intensity		High	High	High	High
Integration		No	No	Yes	Yes
Flexibility/choice		No	No	No	No
Fun factor		Boring/Neutral	Boring/Neutral	Boring/Neutral	Fun
Resonance		Yes	Yes	No	No
Participation		Yes	Yes	Yes	Yes
Education		No	No	No	No
Social environment		No	No	No	No
Physical environment		No	No	No	No
Risk of bias		Low/some concerns	Low/some concerns	Low/some concerns	High
**Predicted mean difference (zBMI):**					
Short:	Low SES	-0.548	-0.228	-0.310	-0.199
	Mixed SES	-0.569	-0.249	-0.332	-0.220
**Medium:**	Low SES	-0.584	-0.264	-0.347	-0.235
	**Mixed SES**	**-0.605***	**-0.285**	**-0.368**	**-0.256**
Long:	Low SES	-0.567	-0.247	-0.330	-0.218
	Mixed SES	-0.588	-0.268	-0.351	-0.239

Table S6. The indicator values associated with the maximum (most positive) mean difference predicted from our primary (random-effects) model. We show the least beneficial combination of indicator values for each of the two age groups (5-11 years and 12-18 years) and the two levels of country income status (high and non-high). We find the same combination regardless of whether we specify follow-up time (short, medium or long) or SES (mixed or low). The resulting predicted mean difference in each scenario is shown at the bottom of the table. For a given age group and income status, the least beneficial effect is obtained for short term follow-up and low SES. Overall, the model predicts the least benefit for the younger age group high-income countries (indicated with an asterisk). Indicators that take the same value in the best combination (Table S5) are highlighted in yellow.

		12-18 years		5-11 years	
**Indicator**		**Non-high income**	**High income**	**Non-high income**	**High income**
School		No	No	No	No
Home		Yes	Yes	Yes	Yes
Community		Yes	Yes	Yes	Yes
Individual		No	No	No	No
Electronic		Yes	Yes	No	Yes
Diet and physical activity		Yes	No	Yes	No
Physical activity		No	Yes	No	Yes
Multi-strategy		No	No	No	No
Duration		Long	Long	Long	Long
Intensity		Low	Low	Low	Low
Integration		Yes	Yes	No	No
Flexibility/choice		Yes	Yes	Yes	Yes
Fun factor		Boring/Neutral	Fun	Boring/Neutral	Fun
Resonance		Yes	No	Yes	Yes
Participation		Yes	Yes	Yes	Yes
Education		Yes	Yes	Yes	Yes
Social environment		Yes	Yes	Yes	Yes
Physical environment		Yes	Yes	Yes	Yes
Risk of bias		Low/some concerns	High	Low/some concerns	High
**Predicted mean difference (zBMI):**					
**Short:**	**Low SES**	**0.024**	**0.245**	**0.211**	**0.261***
	Mixed SES	0.003	0.224	0.190	0.240
**Medium:**	Low SES	-0.013	0.209	0.175	0.225
	**Mixed SES**	-0.034	0.188	0.154	0.204
Long:	Low SES	0.004	0.226	0.192	0.242
	Mixed SES	-0.017	0.205	0.171	0.221

L Sensitivity analysis: separate analyses of different outcome scales

Here we show the results of the sensitivity analyses with different outcome scales analysed separately, i.e., excluding any mapped data. The analysis with zBMI outcomes alone (**Figure S*9***) is based on 171 observations from 110 trials while the BMI only analysis (**Figure S*10***) involves 182 observations from 129 trials. In **Figure S*11*** we also show the results with zBMI and mapped percentile (excluding mapped BMI and proportions) which includes an additional 25 observations from 18 trials compared with the zBMI only analysis.

On the whole, the results for BMI only are similar to the results of the primary analysis, while the zBMI only results are largely uncertain. The heterogeneity parameter is estimated to be smaller in the analyses of individual outcomes than in the full dataset. We summarize the comparisons between the results for the separate outcome scales in **Table S*7*** and **Table S*8***.

We begin by inspecting the effects with probability ≥0.95 in either the random-effects analysis or the fixed effects analysis and probability ≥0.9 in the other (those considered 'robust' in Section J). There is strong evidence that environmental components are less beneficial in all three sensitivity analyses. The beneficial effects of physical activity alone and medium-term follow-up are also corroborated by the separate analyses, but with stronger evidence from BMI and weaker evidence (in the same direction) from zBMI. However, while there is no evidence of an effect of physical activity in the combined zBMI and percentile analysis, the direction is reversed. The observation that interventions are more effective in the older age group appears to be dominated by the BMI data ($\mathrm{P}(<0) = 1$), with no evidence of an age effect from zBMI only ($\mathrm{P}(<0) = 0.31$) or the combined analysis of zBMI and mapped percentile ($\mathrm{P}(<0) = 0.23$).

Of the effects with reasonable evidence (probabilities ≥0.9) in the primary analysis, the beneficial effect of integration and the less beneficial effect of home-based activities are supported by the zBMI alone and combined zBMI and percentile analyses, but with no evidence from BMI alone. The beneficial effect of multi-component strategies is in the same direction in all analyses with strong evidence from zBMI alone ($\mathrm{P}(<0) = 0.96$) and combined zBMI and percentile ($\mathrm{P}(<0) = 0.95$), and slightly weaker evidence from BMI only ($\mathrm{P}(<0) = 0.83$). The only result pointing in opposite directions is for the average effect of high-income countries: BMI strongly indicates a preference for high-income countries ($\mathrm{P}(<0) = 1$), while zBMI finds greater beneficial effects for low-income countries ($\mathrm{P}(>0) = 0.95$). This may be due to a lack of data from low-income countries. The strength of this evidence for the zBMI analysis is increased with the addition of the mapped percentile data ($\mathrm{P}(>0) = 0.98$).

For the interaction terms, we observe no opposing evidence between the analyses on separate outcome scales. However, most of the effects observed in the primary analysis are dominated by one (or two) outcome scales with no evidence on the other(s). For example, the interactions between age and combined diet and physical activity, age and integration, age and income status of country, and activity and income status of country are observed in the BMI only analysis, but there is no evidence of these interactions from zBMI either alone or combined with percentile. Conversely, the interaction between activity and duration is observed in the same direction in all three analyses with the strongest evidence from zBMI and percentile ($\mathrm{P}(>0) = 0.78$), followed by zBMI alone ($\mathrm{P}(>0) = 0.69$) and BMI alone ($\mathrm{P}(>0) = 0.59$).

The only interaction that appears with strong evidence in one of the separate outcome analyses that does not appear in the primary analysis is between interventions with an electronic component and interventions targeting both diet and physical activity. There is strong evidence of a synergistic effect in the BMI only analysis ($\mathrm{P}(<0) = 0.97$), and weaker evidence in the same direction for combined zBMI and percentile ($\mathrm{P}(<0) = 0.89$) and the zBMI only analysis ($\mathrm{P}(<0) = 0.58$).

L.1 zBMI only

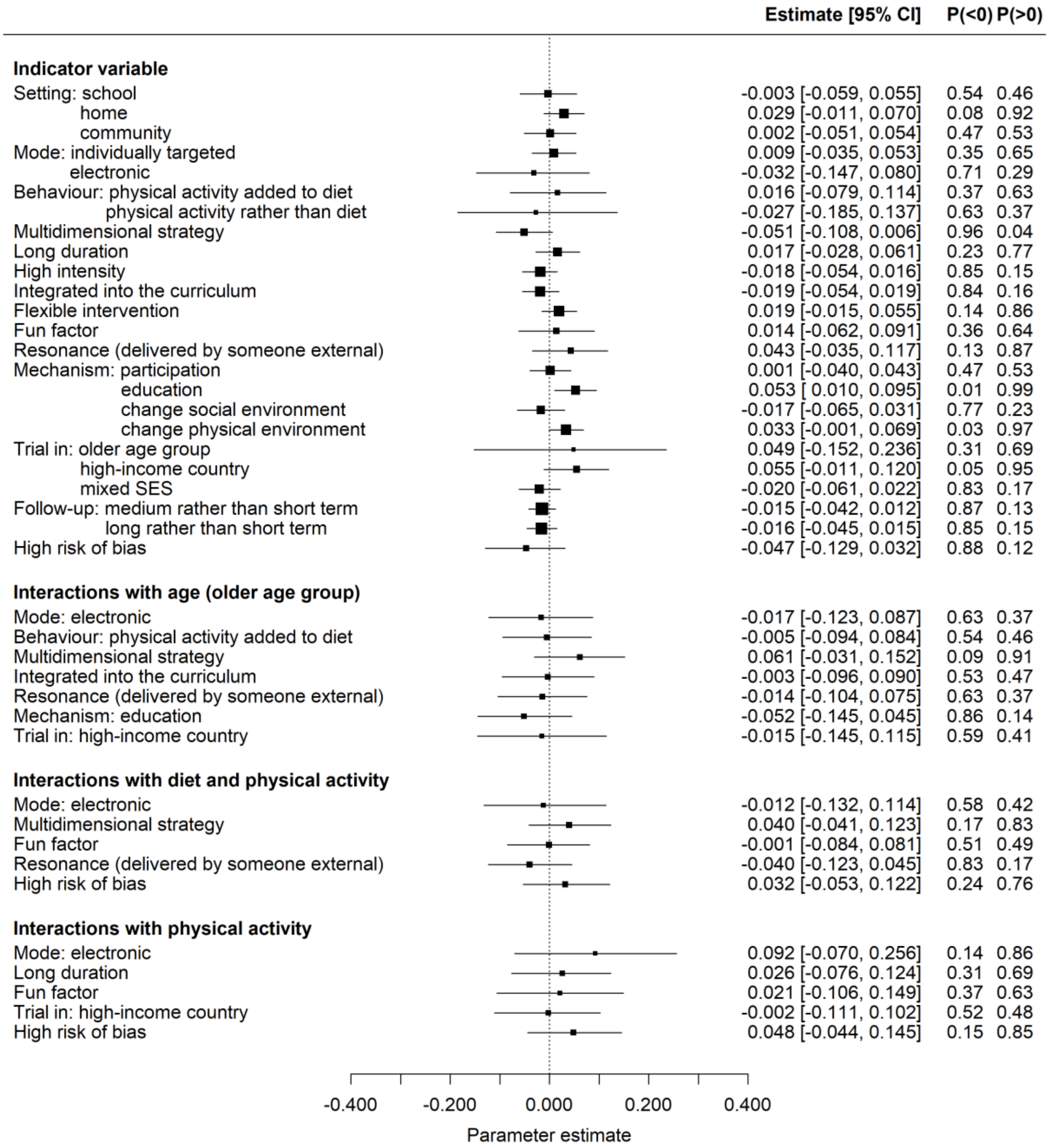

Figure S9: Parameter estimates from the random effects model fitted to data on reported zBMI only. The estimates of the intercept (for centred data) and heterogeneity parameter are $\alpha\ [95\%\ CI] = -0.045[-0.061, -0.029]$ and $\tau\ [95\%\ CI] = 0.049\ [0.037, 0.063]$ respectively.

L.2 BMI only

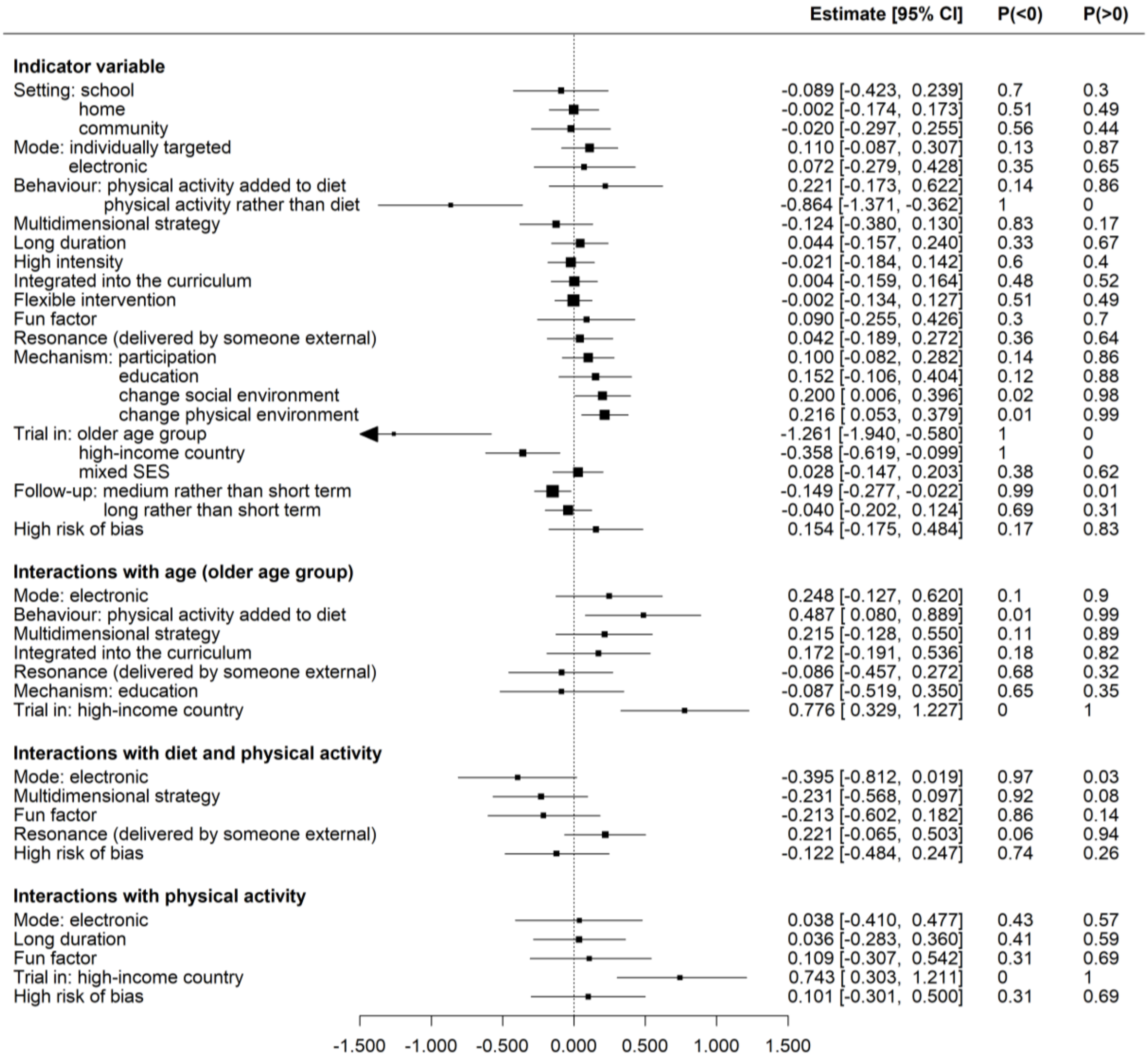

Figure S10: Parameter estimates from the random effects model fitted to data on reported zBMI only. The estimates of the intercept (for centred data) and heterogeneity parameter are $\alpha\ [95\%\ CI] = -0.05 = -0.060[-0.133, -0.015]$ and $\tau\ [95\%\ CI] = 0.271\ [0.219, 0.332]$ respectively.

L.3 zBMI and percentile only

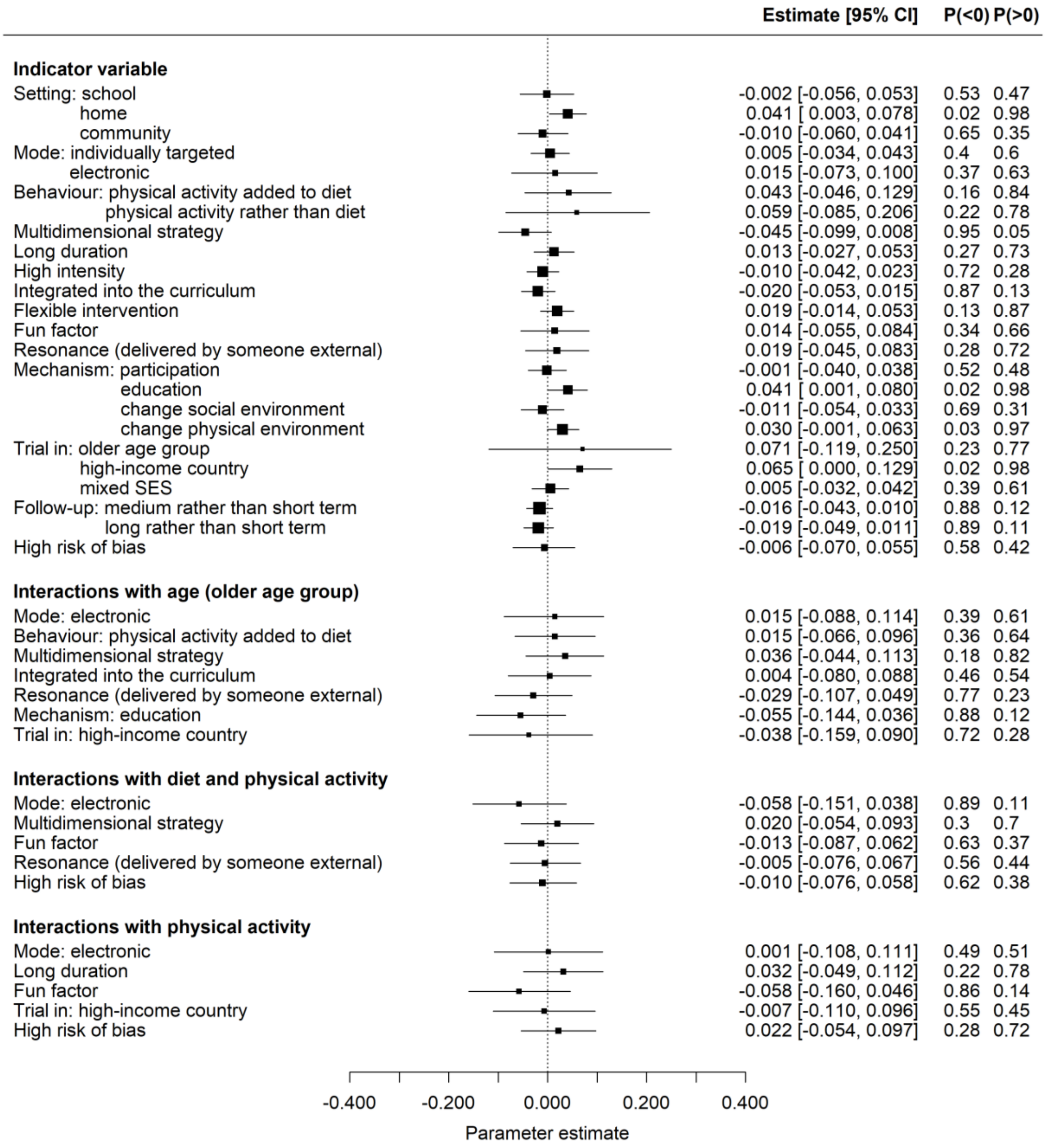

Figure S11: Parameter estimates from the random effects model fitted to data on reported zBMI and mapped percentile (excluding mapped BMI). The estimates of the intercept (for centred data) and heterogeneity parameter are α $[95\%\ CI] = -0.045[-0.060, -0.029]$ and τ $[95\%\ CI] = 0.051\ [0.039, 0.065]$ respectively.

L.4 Comparison of different outcome scales

Table S7: A table summarizing the main differences between the indicator parameters estimated from the analyses on different outcome scales. The primary analysis includes all the reported zBMI data as well as the BMI and percentile data mapped onto the zBMI scale. We list the probability that a parameter estimate falls one side of zero in each analysis (with the corresponding direction indicated in the second column).

Indicator	Direction of effect (in main analysis)	Probabilities in different analyses: Primary	BMI only	zBMI only	zBMI and Percentile
Home	Less beneficial, $P(>0)$	0.95	0.49	0.92	0.98
Physical activity	Beneficial, $P(<0)$	1	1	0.63	0.22
Multi-strategy	Beneficial, $P(<0)$	0.92	0.83	0.96	0.95
Integrated	Beneficial, $P(<0)$	0.93	0.48	0.84	0.87
Social	Less beneficial, $P(>0)$	0.77	0.98	0.23	0.31
Environment	Less beneficial, $P(>0)$	1	0.99	0.97	0.97
Older age group	Beneficial, $P(<0)$	1	1	0.31	0.23
High income country	Beneficial, $P(<0)$	0.95	1	0.05	0.02
Medium term	Beneficial, $P(<0)$	0.99	0.99	0.87	0.88

Table S8: A table summarizing the main differences between the interactions estimated from the analyses on different outcome scales. The primary analysis includes all the reported zBMI data as well as the BMI and percentile data mapped onto the zBMI scale. We list the probability that an interaction estimate falls one side of zero in each analysis (with the corresponding direction indicated in the second column).

Interaction	Direction of effect (in main analysis)	Probabilities in different analyses: Primary	BMI only	zBMI only	zBMI and Percentile
Older age & Diet and physical activity	Antagonistic, $P(>0)$	0.99	0.99	0.46	0.64
Older age & integration	Antagonistic, $P(>0)$	0.94	0.82	0.47	0.54
Older age & high income country	Antagonistic, $P(>0)$	1	1	0.41	0.28
Diet and physical activity & electronic	Synergistic, $P(<0)$	0.84	0.97	0.58	0.89
Physical activity & long duration	Antagonistic, $P(>0)$	0.97	0.59	0.69	0.78
Physical activity & high income country	Antagonistic, $P(>0)$	1	1	0.48	0.45

M Sensitivity analysis: assuming different correlations between repeated measures over time

In **Figure S*12*** and **Figure S*13*** we show the results of the random effects analysis assuming correlations of 0.5 and 0.95 between observations at different time points. We observe negligible differences from the primary analysis across all estimated effects. The estimate of the heterogeneity parameter is smaller for the 0.5 correlation ($0.076\ [0.063, 0.091]$) and larger for the 0.95 correlation ($0.088\ [0.077, 0.010]$).

M.1 Correlation of 0.5

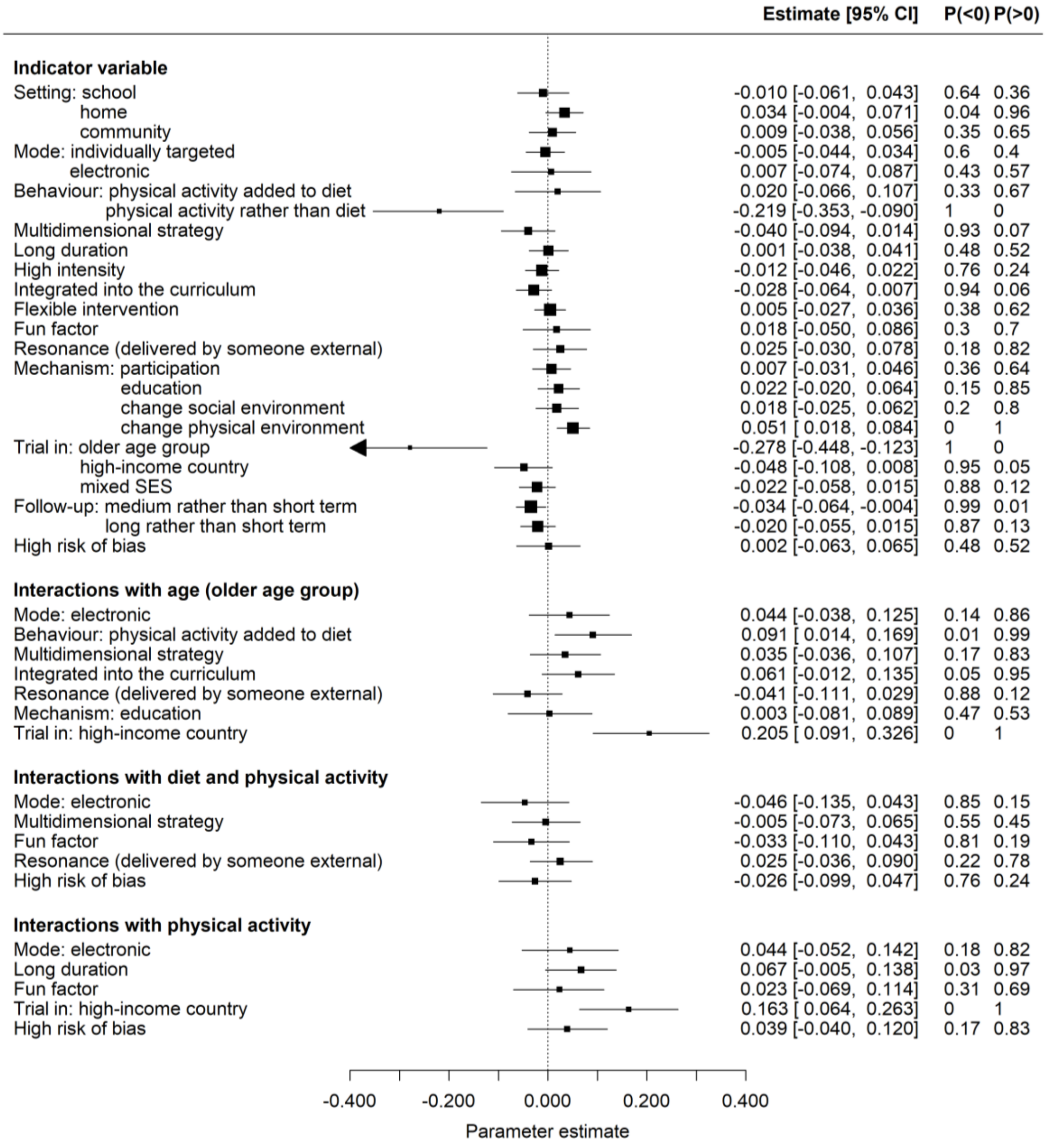

Figure S12: Parameter estimates from the random effects model assuming correlations of $\rho_{y,tt'} = \rho_{d,tt'} = 0.5$ for one degree of separation (short to medium, and medium to long). The estimates of

the intercept (for centred data) and heterogeneity parameter are α $[95\%\ CI] = -0.037$ $[-0.052, -0.022]$ and τ $[95\%\ CI] = 0.076$ $[0.063, 0.091]$ respectively.

M.2 Correlation of 0.95

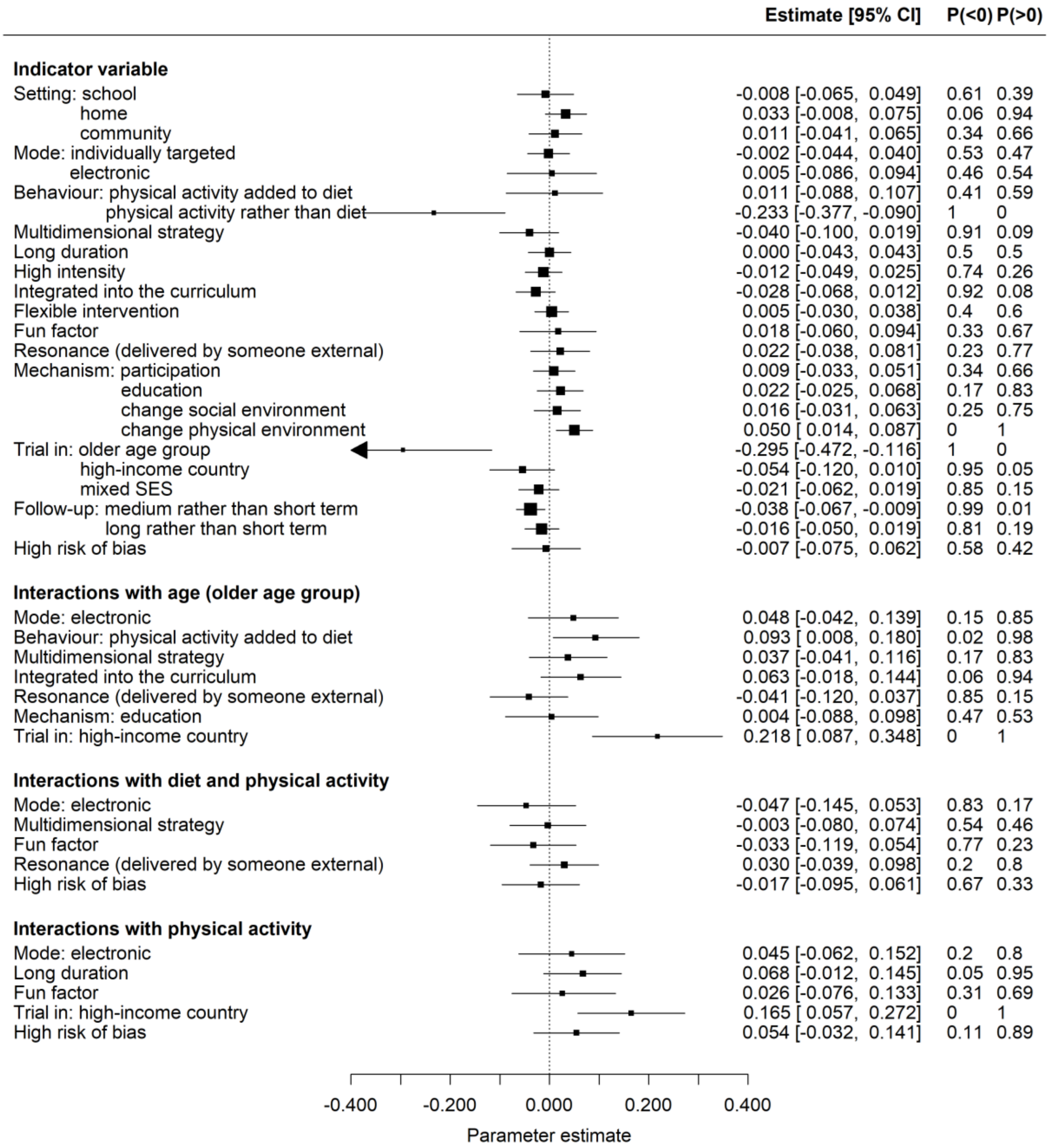

Figure S13: Parameter estimates from the random effects model assuming correlations of $\rho_{y,tt'} = \rho_{d,tt'} = 0.95$ for one degree of separation (short to medium, and medium to long). The estimates of the intercept (for centred data) and heterogeneity parameter are α $[95\%\ CI] = -0.037$ $[-0.053, -0.021]$ and τ $[95\%\ CI] = 0.088$ $[0.077, 0.010]$ respectively.

References for supplementary materials

1. Spiga, F., et al., *A novel analytic framework to investigate differential effects of interventions to prevent obesity in children and young people*. 2024: medrXiv. p. 1-24.
2. Spiga, F., et al., *Interventions to prevent obesity in children aged 5 to 11 years old*. 2024: Cochrane Database of Systematic Reviews TBD.
3. Spiga, F., et al., *Interventions to prevent obesity in children aged 12 to 18 years old*. 2024: Cochrane Database of Systematic Reviews TBD.
4. Davies, A.L. and J.P.T. Higgins, *A complex meta-regression model to identify effective features of interventions from multi-arm, multi-follow-up trials*. 2024: arXiv. p. 1-22.
5. Plummer, M., *JAGS Version 4.3.0 User Manual*. 2017: Online: Available from https://martynplummer.wordpress.com/. Accessed January 2024.
6. Brooks, S.P. and A. Gelman, *General Methods for Monitoring Convergence of Iterative Simulations.* Journal of Computational and Graphical Statistics, 1998. **7**(4): p. 434-455.
7. Andrew, G. and B.R. Donald, *Inference from Iterative Simulation Using Multiple Sequences.* Statistical Science, 1992. **7**(4): p. 457-472.
8. Efthimiou, O., et al., *Bayesian models for aggregate and individual patient data component network meta-analysis.* Stat Med, 2022. **41**(14): p. 2586-2601.
9. Spiegelhalter, D.J., et al., *Bayesian Measures of Model Complexity and Fit.* Journal of the Royal Statistical Society Series B: Statistical Methodology, 2002. **64**(4): p. 583-639.
